# Different Doses of Pharmacological Treatments for Mild to Moderate Alzheimer’s Disease: A Bayesian Network Meta-Analysis

**DOI:** 10.3389/fphar.2020.00778

**Published:** 2020-05-26

**Authors:** Tingting Zhang, Nanyang Liu, Hongfu Cao, Wei Wei, Lina Ma, Hao Li

**Affiliations:** ^1^College of First Clinical Medicine, Shandong University of Traditional Chinese Medicine, Jinan, China; ^2^Department of Geratology, Xiyuan Hospital, China Academy of Chinese Medical Science, Beijing, China; ^3^Institute of Basic Theory for Chinese Medicine, China Academy of Chinese Medical Science, Beijing, China

**Keywords:** Alzheimer’s disease, donepezil, network meta-analysis, pharmacological treatment, randomized controlled trial

## Abstract

**Background:**

Pharmacological treatments play a significant role in treating mild to moderate Alzheimer’s disease (AD), but the optimal doses of various drugs used for these treatments are unknown. Our study compared the efficacy, acceptability, and safety of different doses of pharmacological treatments for mild to moderate AD.

**Methods:**

Randomized controlled trials (RCTs) were identified by searching the PubMed, EMBASE, and Cochrane Library databases (all RCTs published from the date of inception of the databases until September 19, 2019). Trials comparing the efficacy, acceptability, and safety of pharmacological interventions involving donepezil, galantamine, rivastigmine, memantine, huperzine A, and *Ginkgo biloba* extract EGb761, alone or in combination, were identified. The primary outcomes were efficacy, acceptability, and safety.

**Results:**

Our meta-analysis included 37 studies involving 14,705 participants. In terms of improving cognitive function, galantamine 32 mg, galantamine 24 mg, donepezil 5 mg, and donepezil 10 mg were more effective than other interventions, with the surface under the cumulative ranking curve (SUCRA) values of 93.2, 75.5, 73.3, and 65.6%, respectively. According to the SUCRA values, EGb761 240 mg was considered to be the optimal intervention in terms of both acceptability and safety. With regard to clinical global impression, rivastigmine 12 mg had the highest probability of being ranked first (83.7%). The rivastigmine 15 cm^2^ patch (SUCRA = 93.7%) may be the best choice for daily living. However, there were no interventions that could significantly improve neuropsychiatric symptoms, compared with the placebo.

**Conclusions:**

Different doses of the tested pharmacological interventions yielded benefits with regard to cognition, acceptability, safety, function, and clinical global impressions, but not effective behaviors.

## Background

There were an estimated 50 million dementia patients worldwide in 2018. Although this disease currently represents an enormous public health problem, the number of dementia patients is predicted to rise to 152 million by 2050 (Alzheimer’s Disease International, 2018). Alzheimer’s disease (AD) is an irreversible neurodegenerative disease that manifests as progressive memory loss and cognitive dysfunction, and is the leading cause of dementia, accounting for 50–75% of all cases globally (International., Alzheimer’s disease, 2019). There is currently no cure for AD; the typical pharmacological therapeutic goals are to delay disease progression and to improve the patients’ quality of life. Pharmacological treatments approved by the US Food and Drug Administration are mainly grouped into two classes by their differing mechanisms of action: acetylcholinesterase inhibitors (AChEIs), such as donepezil, galantamine, and rivastigmine, which are widely used treatments for mild to moderate disease stages (NICE; Corbett et al., 2012); and N-methyl-D-aspartate receptor antagonists, typically memantine, for moderate to severe disease stages (Kishi et al., 2017).

Donepezil is the primary treatment for mild to moderate AD; it is well tolerated and results in cognitive improvement (Rogers et al., 1998b; Rogers et al., 1998a; Burns et al., 1999). Moreover, evidence suggests that donepezil has dose-dependent effects: with increasing doses, its efficacy improves, although more adverse events also occur. Increased improvements in cognition are indicated for donepezil 10 mg but not donepezil 5 mg, especially at 18 and 24 weeks, based on the meta-analysis of Whitehead et al., which included 10 clinical trials (Whitehead et al., 2004). In routine practice, the variety of different drug preparations and dosages poses a challenge for physicians responsible for decision-making with regard to treatment options for AD.

EGb761, extracted from *Ginkgo biloba*, is a common herbal treatment for AD (Akram and Nawaz, 2017). A previous systematic review and meta-analysis demonstrated that compared with placebo, the *Ginkgo biloba* extract EGb761 appeared to have stronger cognitive effects (standard mean difference [SMD] = −0.58, 95% confidence interval [CI]: −1.14, −0.01) (Weinmann et al., 2010). Although the efficacy of the *Ginkgo biloba* extract EGb761 was confirmed, when compared with donepezil, the results were not conclusive (Mazza et al., 2006; Yancheva et al., 2009; Nasab et al., 2012). In addition, a Cochrane systematic review of six trials suggested that huperzine A, a reversible and selective AChEI, is likely beneficial to AD patients and resulted in no apparent serious adverse events (Li et al., 2008). To date, a direct comparison of huperzine A, EGb761, an AChEI, or memantine has not been conducted in the same study.

It should be noted that a previous network meta-analysis focused on the comparative effectiveness of different anti-dementia treatments by using direct or indirect evidence, but did not consider different drug doses (Thancharoen and Limwattananon, 2019) or include comprehensive pharmacological interventions (Dou et al., 2018). A network meta-analysis allows the summation of direct and indirect evidence from relevant randomized controlled trials (RCTs) and the performance of an integrated analysis to determine the optimal pharmacological therapy for mild to moderate AD (Higgins and Whitehead, 1996). Therefore, this study aimed to comprehensively evaluate the efficacy (i.e., improvements in cognitive function), acceptability (i.e., completion of treatment), and safety (i.e., number of adverse events) of different doses of pharmacological agents used for treating mild to moderate AD, which can be used to inform clinical practice.

## Methods

### Search Strategy

This network meta-analysis was performed in accordance with the guidelines of the Preferred Reporting Items for Systematic Reviews and Meta-Analysis (PRISMA) extension for network meta-analysis (Hutton et al., 2015). Relevant RCTs were identified in titles and abstracts in the PubMed, EMBASE, and the Cochrane Library databases. Results were restricted to English language publications from the date of the database inception to September 19, 2019. No restrictions were placed on publication dates or status. We adopted the MeSH and Emtree terms “Alzheimer’s disease,” “donepezil,” “galantamine,” “rivastigmine,” “memantine,” “huperzine A,” “*Ginkgo biloba* extract,” and “randomized controlled trials” combined with the corresponding free words adapted appropriately for each of the databases in the search algorithm. Additionally, we manually searched the references from the cited articles to identify meta-analyses and RCTs to avoid missing potentially eligible clinical trials. The details of the search strategies involving different databases are described in the **Additional file: Supplementary 1**.

### Selection Criteria

The selection criteria were based on the principle of the Population-Intervention-Comparator-Outcomes-Study design (PICOS) (Costantino et al., 2015). The eligible studies were RCTs and had to meet the following criteria: 1) participants were clinically diagnosed with AD in accordance with the criteria of the Diagnostic and Statistical Manual of Mental Disorders (DSM) or the National Institute of Neurological and Communicative Disorders and Stroke and the Alzheimer’s Disease and Related Disorders Association (NINCDS-ADRDA) (McKhann et al., 1984). Mild to moderate AD was classified by a score of 10–26 (inclusive) in the Mini-Mental State Examination (MMSE) (Folstein et al., 1975); 2) trials compared the effectiveness of pharmacological interventions using donepezil, galantamine, rivastigmine, memantine, huperzine A, or *Ginkgo biloba* extract alone or in combination, and drug dosages were not only within the therapeutic range but were also specific; 3) outcome measures covered at least one of the following outcomes: cognitive, global assessment, behavior, function, acceptability, or safety; and 4) the duration of follow-up was between 12 and 104 weeks. The following exclusion criteria were applied: 1) RCTs that recruited fewer than 10 participants in each group; 2) unavailability of the full text of the study, even with the support of expert librarians; and 3) participants diagnosed with other types of dementia or neurological disorders unrelated to AD, or outcome data for participants with AD that could not be independently assessed apart from data for participants diagnosed with other types of dementia.

### Data Extraction

Two investigators (LN and CH) independently extracted the relevant data from all eligible studies published in English using predefined standardized spreadsheets. All extracted data were based on intention-to-treat analysis. Any discrepancies were resolved to consensus by two investigators (LN and CH) or arbitrated by a third investigator (ZT). The following information was documented for every study: first author, publication year, detailed trial information, diagnostic criteria, patient characteristics (i.e., age, gender, race, and baseline MMSE scores), treatment (dose, frequency), sample size, outcomes of the change from baseline (cognitive, global assessment, behavior, function), number of treatment completion, incidences of adverse events, and the duration of follow-up. Finally, all extracted data were cross-checked by two investigators (LN and CH) to ensure accuracy.

### Quality Assessment

We evaluated the quality of the included trials using the Cochrane Collaboration’s risk of bias assessment tool (Higgins et al., 2011), and the trials were judged to have a low risk of bias, an unclear risk of bias, or a high risk of bias. Any discrepancies between the two authors’ evaluations (ZT and LN) were resolved by discussion.

### Outcome Measures

We considered the overall mean change in cognitive function from the baseline to the study endpoint, the number of patients who completed the trial during the treatment period, and the number of patients who experienced any adverse events for our primary outcomes, as these were the most consistently reported estimates of efficacy, acceptability, and safety of interventions for mild to moderate AD. Cognitive function was primarily appraised by the Alzheimer’s Disease Assessment Scale-cognition subscale (ADAS-cog), and the MMSE. For secondary outcome measures, we also estimated the changes from baseline to the endpoints of cognitive function, behavioral symptoms, and the clinical global impressions of patients, which were assessed by the Alzheimer’s Disease Cooperative Study-Activities of Daily Living (ADCS-ADL) scale, the Neuropsychiatric Inventory (NPI), and the Clinician’s Interview-Based Impression of Change plus Caregiver Input (CIBIC-Plus) scale, respectively.

### Statistical Analysis

First, we estimated the SMD for continuous outcomes and odds ratios (ORs) for dichotomous outcomes along with the corresponding 95% confidence interval (CI) by using a random-effects model, which served as the pooled effect sizes in conventional pair-wise meta-analysis. To assess the statistical heterogeneity of the direct comparison in the quantitative analysis, we used the *I*^2^ statistic and p values. Stata software version 14.0 (Stata Corporation, College Station, TX, USA) was used for all analyses.

Second, for all collected outcomes, we performed a Bayesian network meta-analysis combining direct and indirect comparisons based on a random-effect model considering the smaller deviance information criteria (DIC) value. The data analysis used OpenBUGS software (version 3.2.3), and the network diagram was produced using Stata software (version 14.0). We chose various initial values at random with the run of three Markov chains simultaneously. The total number of iterations was 30,000. The median of the calculated data served as pooled estimated effect sizes (SMD or OR), and the 2.5 and 97.5 percentiles served as the corresponding 95% credible interval (CrI). The statistical significance was evaluated in line with whether the CrI included 0 or 1. Moreover, we also calculated the surface under the cumulative ranking curve (SUCRA) to rank the interventions for each outcome in which the SUCRA value was closely related to the rank of each intervention. In addition, if the network of interventions had closed loops, the node-splitting method and loop-specific method were performed to evaluate the statistical inconsistency (Salanti et al., 2008; Dias et al., 2010; Veroniki et al., 2013). The determination of whether the loop consistency was significant depended on the CI of the inconsistency factor (IF) value containing 0. Finally, for the small-sample effect assessment of intervention networks, we constructed a comparison-adjusted funnel plot and performed a visual assessment.

## Results

### Literature Search Results

**Figure 1:**
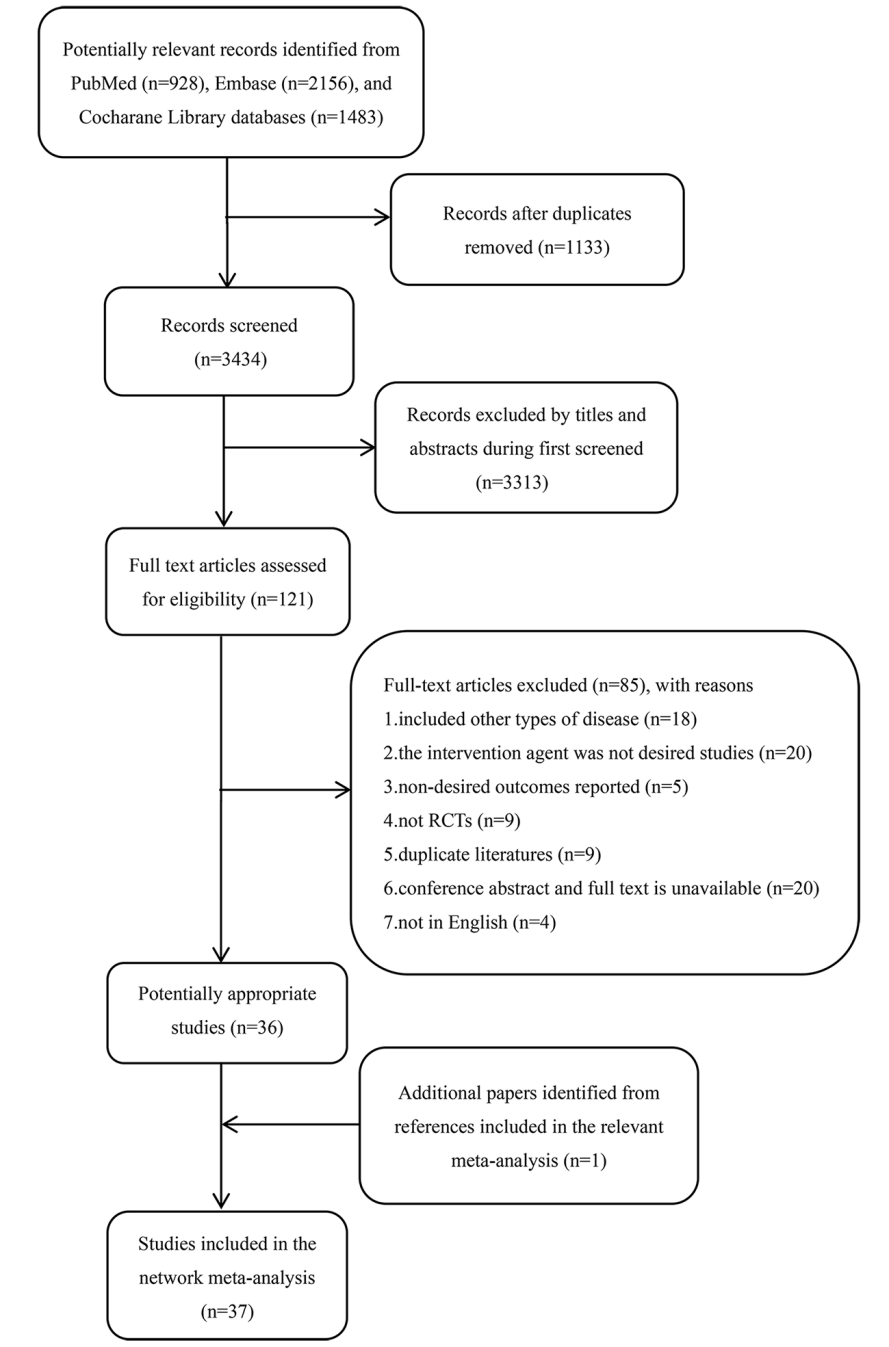
The PRISMA flowchart for detailed search results and selection. RCT, randomized controlled trial.

In total, 4,567 citations were identified by searching the PubMed, EMBASE, and the Cochrane Library databases. After 1,133 duplicate citations were removed using Endnote X7 software, the titles and abstracts for 3,434 citations were retrieved. Subsequently, the full text of 121 potentially eligible studies were reviewed further. From these, 85 publications were excluded primarily because they included other diseases (n = 18), did not report the desired intervention agents (n = 20), reported undesired outcomes (n = 5), were not RCTs (n = 9), were duplicate studies (n = 9), were not in English (n = 4), or were conference abstracts without available full texts (n = 20). Finally, 36 eligible studies met the inclusion criteria. In addition, we identified an additional publication from the references. Overall, 37 studies (Rogers et al., 1998a; Rogers et al., 1998b; Burns et al., 1999; Rosler et al., 1999; Homma et al., 2000; Raskind et al., 2000; Tariot et al., 2000; Wilcock et al., 2000; Wilkinson and Murray, 2001; Winblad et al., 2001; Jones et al., 2004; Seltzer et al., 2004; Brodaty et al., 2005; Karaman et al., 2005; Schneider et al., 2005; Johannsen et al., 2006; Mazza et al., 2006; Peskind et al., 2006; Rockwood et al., 2006; Feldman and Lane, 2007; Winblad et al., 2007; Bakchine and Loft, 2008; Yancheva et al., 2009; Choi et al., 2011; Frolich et al., 2011; Maher-Edwards et al., 2011; Nakamura et al., 2011; Rafii et al., 2011; Cummings et al., 2012; Ihl et al., 2012; Zhang et al., 2012; Hager et al., 2014; Haig et al., 2014; Marek et al., 2014; Gault et al., 2015; Zhang et al., 2015; Zhang et al., 2016) were available for inclusion in the network meta-analysis. The PRISMA flowchart detailing the literature search process is shown in **Figure 1**.

### Characteristics of the Eligible Studies

The characteristics of the included studies and details of the patients are shown in **Table 1**. The 37 studies involving 14,705 participants contributed to the network meta-analysis. Across all trials, the year of RCT publication ranged from 1998 to 2016. The mean study sample size was 175 participants in each group, with a range between 20 and 1,024 patients. The mean (SD) age of participants was between 64.2 (8.4) and 78.1 (8.35) years of age. The minimum percentage of females was 45.6%, and the maximum percentage was 84.4%. Most trials (35 [94.6%] of 37) adopted the NINCDS-ADRDA diagnostic criteria. Follow-up data was available for all patients for a minimum of 12 weeks and a maximum of 104 weeks.

**Table 1:** Description of included studies and patient characteristics.

Study	Treatment	N	Age Mean (SD)	Gender (% female)	Baseline MMSE Mean (SD)	Criteria	Duration (weeks)
Zhang et al., 2016	Rivastigmine patch 10 cm^2^	248	70.4 (8.02)	56.5	16.0 (3.46)	NINCDS-ADRDA	24
	Rivastigmine 12 mg	253	69.8 (8.20)	54.9	16.6 (3.08)		
Zhang et al., 2015	Memantine 20 mg	80	69.75 (8.06)	61.25	15.88 (4.43)	NINCDS-ADRDA	24
	Donepezil 10 mg	87	70.13 (7.99)	59.77	15.53 (4.22)		
Hager et al., 2014	Galantamine 24 mg	1,024	73.0 (8.9)	65.5	19.0 (4.12)	NINCDS-ADRDA	104
	Placebo	1,021	73.0 (8.7)	64.1	19.0 (4.04)		
Zhang et al., 2012	Galantamine 24 mg	116	73.3 (8.5)	51	18.8 (3.8)	NINCDS-ADRDA	16
	Donepezil 10 mg	117	74.0 (8.4)	55	17.9 (4.1)		
Ihl et al., 2012	EGb761 240 mg	163	64.9 (9.5)	66.9	NA	NINCDS-ADRDA	24
	Placebo	170	64.2 (8.7)	65.3	NA		
Rafii et al., 2011	Huperzine A 400 µg	68	77.57 (8.79)	60.29	19.00 (4.26)	NINCDS-ADRDA	16
	Huperzine A 200 µg	69	78.06 (6.91)	68.12	19.25 (4.20)		
	Placebo	73	78.1 (8.35)	64.38	19.12 (4.00)		
Choi et al., 2011	Rivastigmine patch 10 cm^2^ + Memantine 20 mg	88	75.0 (7.3)	75	16.8(4.3)	NINCDS-ADRDA	24
	Rivastigmine patch 10 cm^2^	84	74.7 (7.7)	84.34	16.4(4.7)		
Yancheva et al., 2009	EGb761 240 mg	31	69.0 (8.0)	54.8	NA	NINCDS-ADRDA	22
	Donepezil 10 mg	33	66.0 (8.0)	84.4	NA		
	EGb761 240 mg + Donepezil 10 mg	32	68.0 (9.0)	67.7	NA		
Winblad et al., 2007	Rivastigmine patch 10 cm^2^	293	73.6 (7.9)	68	16.6 (3.1)	DSM-IV NINCDS-ADRDA	24
	Rivastigmine 12 mg	297	72.8 (8.2)	65.6	16.4 (3.1)		
	Placebo	302	73.9 (7.3)	66.6	16.4 (3.0)		
Winblad et al., 2001	Donepezil 10 mg	142	72.1 (8.6)	69.7	19.37 (4.37)	DSM-IV NINCDS-ADRDA	52
	Placebo	144	72.9 (8.0)	59	19.26 (4.54)		
Wilkinson and Murray, 2001	Galantamine 24 mg	56	72.9 (8.2)	59	18.2 (3.0)	DSM-III-R NINCDS-ADRDA	12
	Placebo	87	74.2 (8.4)	59	18.7 (2.8)		
Wilcock et al., 2000	Galantamine 24 mg	220	71.9 (8.3)	63.18	19.5 (3.4)	NINCDS-ADRDA	24
	Galantamine 32 mg	218	72.1 (8.6)	63.3	19.0 (3.8)		
	Placebo	215	72.7 (7.6)	61.4	19.3 (3.5)		
Tariot et al., 2000	Galantamine 24 mg	273	77.7 (6.6)	67.03	17.7 (3.3)	NINCDS-ADRDA	20
	Placebo	286	77.1 (8.5)	62.24	17.7 (3.4)		
Seltzer et al., 2004	Donepezil 10 mg	96	73.3 (9.6)	50	24.1 (1.7)	DSM-IV NINCDS-ADRDA	24
	Placebo	57	75.1 (8.8)	60	24.3 (1.3)		
Schneider et al., 2005	EGb761 240 mg	170	78.1 (7.0)	56.0	17.9 (4.0)	DSM-IV NINCDS-ADRDA	26
	Placebo	174	77.5 (7.4)	52.0	18.2 (4.1)		
Rosler et al., 1999	Rivastigmine 12 mg	243	72.0	59.0	19.9	DSM-IV NINCDS-ADRDA	26
	Placebo	239					
Raskind et al., 2000	Galantamine 24 mg	212	75.9 (7.3)	65.57	19.5 (4.4)	NINCDS-ADRDA	24
	Galantamine 32 mg	211	75.0 (8.7)	58.77	19.1 (4.4)		
	Placebo	213	75.3 (8.8)	61.5	19.2 (4.4)		
Peskind et al., 2006	Memantine 20 mg	201	78.0 (7.3)	60.2	17.4 (3.7)	NINCDS-ADRDA	24
	Placebo	202	77.0 (8.2)	57.43	17.2 (3.4)		
Nakamura et al., 2011	Rivastigmine patch 5 cm^2^	282	74.3 (7.5)	68.8	16.8 (2.9)	DSM-IV NINCDS-ADRDA	24
	Rivastigmine patch 10 cm^2^	287	75.1 (6.9)	67.9	16.5 (3.1)		
	Placebo	286	74.5 (7.4)	68.2	16.6 (2.9)		
Mazza et al., 2006	EGb761 160 mg	25	66.2 (6.0)	52.0	18.80 (3.62)	DSM-IV	24
	Donepezil 5 mg	25	64.5 (6.0)	48.0	18.55 (3.47)		
	Placebo	26	69.8 (3.0)	61.0	18.80 (3.63)		
Rockwood et al., 2006	Galantamine 24 mg	64	77.0 (8.0)	64.0	20.8 (3.3)	NINCDS-ADRDA	16
	Placebo	66	78.0 (8.0)	62.0	19.9 (4.2)		
Karaman et al., 2005	Rivastigmine 12 mg	24	74.11 (4.3)	54.17	11.40 (1.0)	DSM-IV NINCDS-ADRDA	52
	Placebo	20	73.40 (4.0)	55	13.20 (0.9)		
Jones et al., 2004	Donepezil 10 mg	64	73.8 (7.4)	51.6	18.3 (3.3)	DSM-IV NINCDS-ADRDA	12
	Galantamine 24 mg	56	75.1 (7.7)	71.4	18.4 (3.7)		
Bakchine and Loft, 2008	Memantine 20 mg	318	74.0 (7.4)	65.0	18.6 (3.3)	DSM-IV NINCDS-ADRDA	24
	Placebo	152	73.3 (6.9)	60.0	18.9 (3.2)		
Brodaty et al., 2005	Galantamine 24 mg	327	76.5 (7.77)	64.0	17.80 (4.14)	NINCDS-ADRDA	26
	Placebo	324	76.3 (8.03)	64.0	18.08 (4.08)		
Cummings et al., 2012	Rivastigmine patch 15 cm^2^	280	75.6 (7.4)	66.1	14.1 (4.8)	DSM-IV NINCDS-ADRDA	48
	Rivastigmine patch 10 cm^2^	287	75.9 (6.8)	63.4	14.2 (4.6)		
Gault et al., 2015	Donepezil 10 mg	68	72.4 (8.42)	45.6	19.6 (3.82)	NINCDS-ADRDA	12
	Placebo	68	73.6 (8.23)	61.8	19.7 (3.95)		
Haig et al., 2014	Donepezil 10 mg	60	70.5 (8.31)	60.0	18.1 (4.1)	NINCDS-ADRDA	12
	Placebo	63	70.3 (7.84)	61.9	18.2 (3.9)		
Marek et al., 2014	Donepezil 10 mg	66	71.8 (8.4)	53.0	19.3 (3.7)	NINCDS-ADRDA	12
	Placebo	66	71.7 (9.0)	60.6	19.4 (3.7)		
Rogers et al., 1998	Donepezil 5 mg	154	72.9 (7.5)	63	19.0 (5.0)	DSM-III-R NINCDS-ADRDA	24
	Donepezil 10 mg	157	74. 6 (7.5)	62	18.9 (5.0)		
	Placebo	162	72.6 (7.6)	61	19.2 (5.1)		
Johannsen et al., 2006	Donepezil 10 mg	99	74.1 (7.6)	59.6	18.8 (4.8)	NINCDS-ADRDA	12
	Placebo	103	71.4 (9.3)	63.1	18.5 (4.8)		
Frolich et al., 2011	Donepezil 10 mg	161	73.9 (6.48)	65.8	NA	NINCDS-ADRDA	12
	Placebo	164	73.5 (6.42)	55.2	NA		
Feldman and Lane, 2007	Rivastigmine 12 mg	227	71.4 (7.9)	60	18.3 (4.5)	DSM-IV NINCDS-ADRDA	26
	Placebo	222	71.7 (8.7)	60	18.7 (4.6)		
Rogers et al., 1998	Donepezil 5 mg	157	73.8 (8.4)	69	19.4 (4.9)	DSM-III-R NINCDS-ADRDA	12
	Donepezil 10 mg	158	73.4 (8.2)	61	19.4 (5.0)		
	Placebo	153	74.0 (8.0)	61	19.8 (4.3)		
Burns et al., 1999	Donepezil 5 mg	271	72.0 (8.2)	61	20.0 (4.9)	DSM-III-R NINCDS-ADRDA	24
	Donepezil 10 mg	273	72.0 (8.3)	57	20.0 (3.3)		
	Placebo	274	71.0 (8.3)	55	20.0 (5.0)		
Maher-Edwards et al., 2011	Donepezil 10 mg	67	71.1 (8.39)	63	19.2(3.20)	DSM-IV NINCDS-ADRDA	24
	Placebo	63	71.6 (6.72)	70	18.3(3.36)		
Homma et al., 2000	Donepezil 5 mg	116	70.1 (7.6)	68	17.8 (3.9)	DSM-IV	24
	Placebo	112	69.4 (8.8)	66	16.6 (3.9)		

DSM-IV, Diagnostic and Statistical Manual of Mental Disorders, Fourth Edition; DSM-III-R, Diagnostic and Statistical Manual of Mental Disorders third edition, revision; NINCDS-ADRDA, The National Institute of Neurological and Communicative Disorders Association and Stroke-AD and Related Disorders Association; MMSE, Mini-mental State Examination; NA, Not available.

### Quality of the Assessment

**Figure 2:**
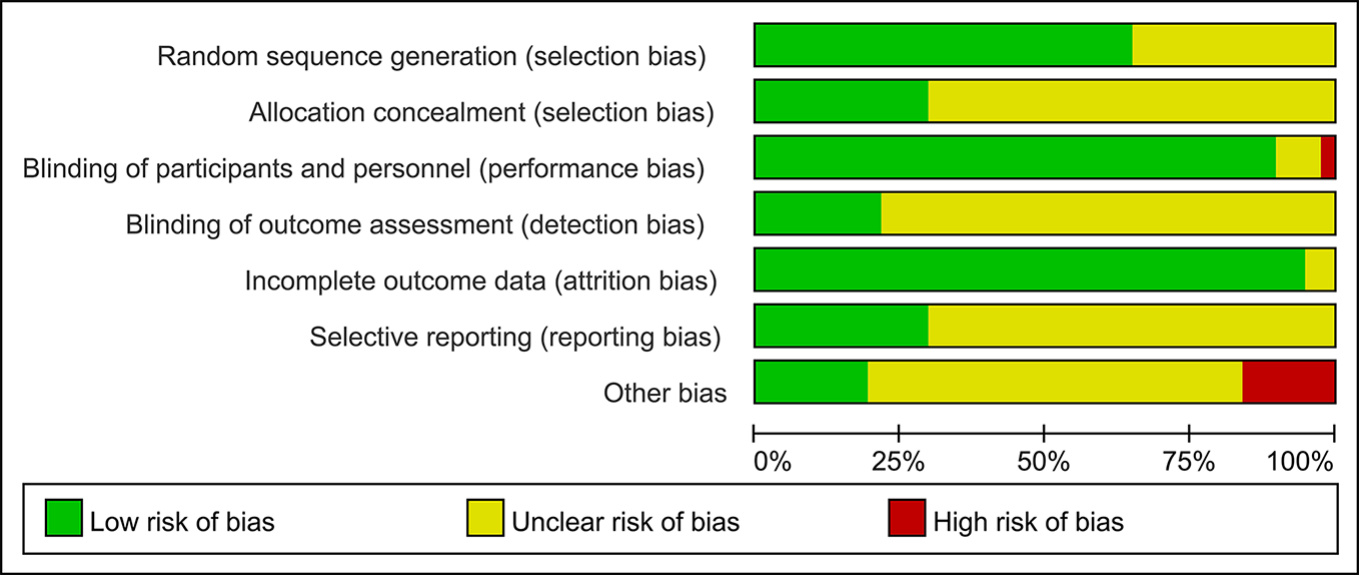
Risk of bias graph presented as percentage across all studies (green represents low risk of bias; red represents high risk of bias; and yellow represents an unclear risk of bias).

Detailed information regarding the risk of bias in all 37 studies is presented in **Figure 2** and **Additional file: Supplementary 2**. It was difficult to assess the risk of selection bias in most studies, owing to the absence of adequate details recorded for randomization and allocation concealment. We identified one study with a high risk of bias associated with the blinding of participants and personnel. As for the blinding of the outcome assessment, 29 trials were rated as having an unclear risk of bias, and only eight studies had evidence indicating a low risk of bias. Most studies (36 of 37) had a low risk of bias for incomplete outcome data. The percentage of studies with unclear bias was 70.3. In addition, a high risk of bias was noted in six studies. In total, the overall quality of the studies was judged to be good.

### Pair-Wise Meta-Analysis

The tested interventions, except for rivastigmine 12 mg, the rivastigmine 5 cm^2^ patch, huperzine A 400 µg, and huperzine A 200 µg, showed statistically significant differences with regard to the ADCS-cog assessment for mild to moderate AD when compared with the placebo. However, in the MMSE, donepezil 10 mg, donepezil 5 mg, rivastigmine 10 cm^2^, galantamine 24 mg, huperzine A 400 µg, and huperzine A 200 µg was superior to the placebo. In terms of acceptability, well-tolerated interventions included rivastigmine 12 mg, rivastigmine 10 cm^2^ patch, rivastigmine 5 cm^2^ patch, galantamine 24 mg, and galantamine 32 mg compared with the placebo. For all interventions, except for donepezil 5 mg, rivastigmine 10 cm^2^ patch, memantine 20 mg, and EGb761 240 mg, adverse events occurred more often than that with the placebo. For secondary outcomes, in terms of daily living, either the rivastigmine 10 cm^2^ patch or galantamine 24 mg was superior to placebo. Compared with placebo, donepezil 10 mg, donepezil 5 mg, rivastigmine 12 mg, the rivastigmine 10 cm^2^ patch, and the rivastigmine 5 cm^2^ patch showed statistically significant differences with regard to the clinical global assessment in patients with mild to moderate AD. Compared with the placebo, only galantamine 24 mg and EGb761 240 mg improved behavioral symptoms. Heterogeneity was found only in the direct comparisons of memantine 20 mg vs. placebo (*I*^2^ = 83.1%), galantamine 24 mg *vs.* placebo (*I*^2^ = 78.0%), and rivastigmine 12 mg *vs.* placebo (*I*^2^ = 76.9%), with *I*^2^ values greater than 70%. These results of the pair-wise meta-analyses are outlined in detail in **Additional file: Supplementary 3**.

### Network Meta-Analysis—Primary Outcomes

**Figure 3:**
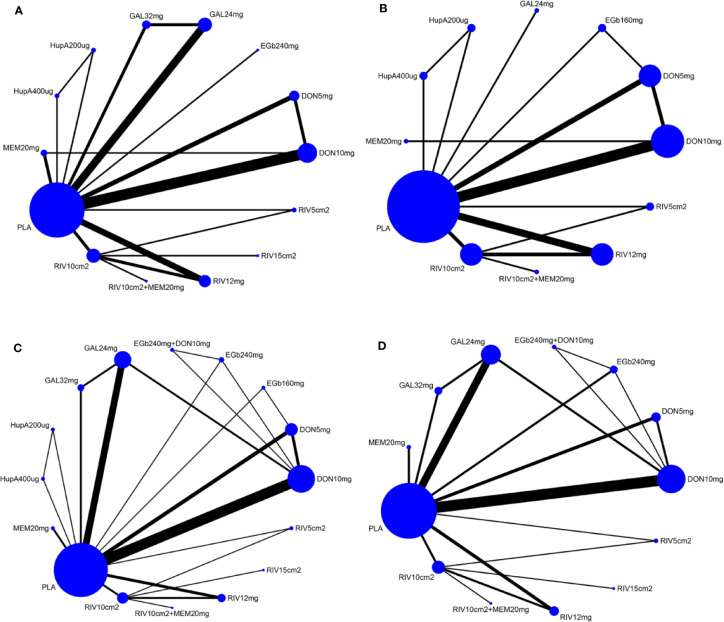
Network of eligible comparisons for all pharmacological treatments included in the analyses [**(A)** according to ADAS-cog scale, **(B)** MMSE results, **(C)** acceptability, **(D)** safety]. Treatments with direct comparisons are linked with a black line; its width is proportional to the number of trials evaluating every pair of the comparison. Blue Nodes represent different treatments. Node size is proportional to the total number of patients for each treatment in the network. MMSE, Mini-Mental State Examination; ADAS-cog, Alzheimer’s Disease Assessment Scale-cognition subscale; PLA, Placebo; RIV10cm2, Rivastigmine patch 10 cm^2^; RIV10cm2+MEM20mg, Rivastigmine patch 10 cm^2^ + Memantine 20 mg; RIV12mg, Rivastigmine 12 mg; RIV15cm2, Rivastigmine patch 15 cm^2^; RIV5cm2, Rivastigmine patch 5 cm^2^; DON10mg, Donepezil 10 mg; DON5mg, Donepezil 5 mg; EGb240mg, EGb761 240 mg; GAL24mg, Galantamine 24 mg; GAL32mg, Galantamine 32 mg; HupA200µg, Huperzine A 200 µg; HupA400µg, Huperzine A 400 µg; MEM20mg, Memantine 20 mg; EGb160mg, EGb761 160 mg; EGb240mg+DON10mg, EGb761 240 mg + Donepezil 10 mg.

**Figure 4:**
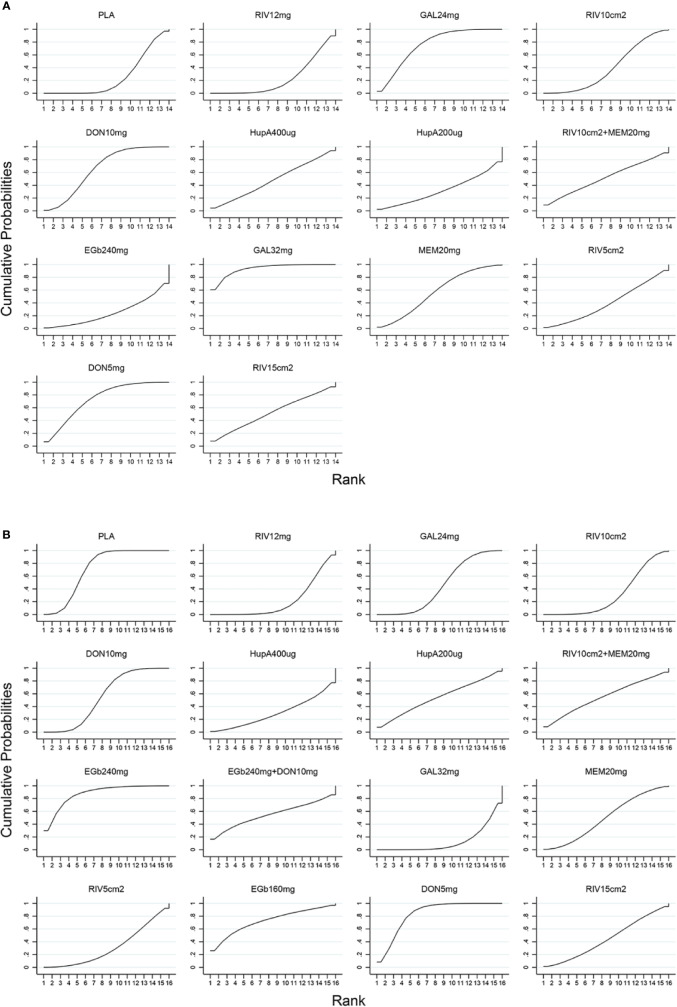
SUCRA for cognitive function based on ADAS-cog scale **(A)** and acceptability **(B)**. The larger the SUCRA, the higher the ranking. ADAS-cog, Alzheimer’s Disease Assessment Scale-cognition subscale; SUCRA, surface under the cumulative ranking curve; PLA, Placebo; RIV12mg, Rivastigmine 12 mg; GAL24mg, Galantamine 24 mg; RIV10cm2, Rivastigmine patch 10 cm^2^; DON10mg, Donepezil 10 mg; HupA400µg, Huperzine A 400 µg; HupA200µg, Huperzine A 200 µg; RIV10cm2+MEM20mg, Rivastigmine patch 10 cm^2^ + Memantine 20 mg; EGb240mg, EGb761 240 mg; GAL32mg, Galantamine 32 mg; MEM20mg, Memantine 20 mg; RIV5cm2, Rivastigmine patch 5 cm^2^; DON5mg, Donepezil 5 mg; RIV15cm2, Rivastigmine patch 15 cm^2^; EGb240mg+DON10mg, EGb761 240 mg + Donepezil 10 mg; EGb160mg, EGb761 160 mg.

**Figure 5:**
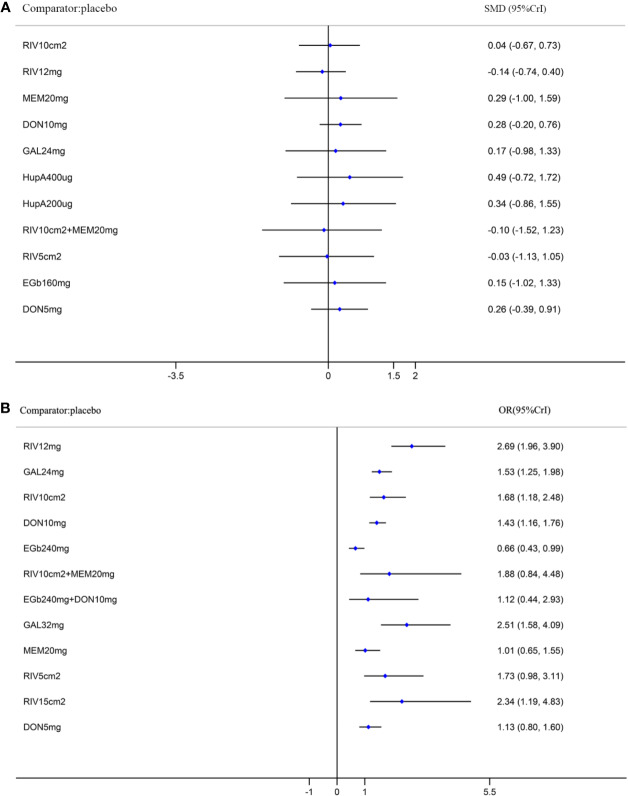
Forest plots of the results of network meta-analysis for function in the MMSE **(A)** and for safety **(B)** compared with placebo. SMD, standardized mean difference; OR, odds ratio; CrI, credible interval; MMSE, Mini-Mental State Examination; RIV10cm2, Rivastigmine patch 10 cm^2^; RIV12mg, Rivastigmine 12 mg; MEM20mg, Memantine 20 mg; DON10mg, Donepezil 10 mg; GAL24mg, Galantamine 24 mg; HupA400µg, Huperzine A 400 µg; HupA200µg, Huperzine A 200 µg; RIV10cm2+MEM20mg, Rivastigmine patch 10 cm^2^ + Memantine 20 mg; RIV5cm2, Rivastigmine patch 5 cm^2^; EGb160mg, EGb761 160 mg; DON5mg, Donepezil 5 mg; EGb240mg, EGb761 240 mg; EGb240mg+DON10mg, EGb761 240 mg + Donepezil 10 mg; GAL32mg, Galantamine 32 mg; RIV15cm2, Rivastigmine patch 15 cm^2^.

A network diagram of all the eligible comparisons involving 24 trials of cognitive function based on the ADAS-cog scale is presented in **Figure 3A**. As outlined in **Figure 3A**, the placebo was the most common comparator in all interventions comparisons; only the rivastigmine 15 cm^2^ patch and the combination of rivastigmine 10 cm^2^ and memantine 20 mg were not directly compared with the placebo. Six closed loops existed across all comparisons. Based on the inconsistency factors (IFs) and 95% CIs, we concluded that the direct and indirect evidence was consistent. The relevant inconsistency results and the figures are shown in **Additional file: Supplementary 4**. In terms of improving cognitive function, galantamine 24 mg, galantamine 32 mg, donepezil 10 mg, and donepezil 5 mg were more effective than placebo, with SMDs of −0.39 (95% CrI: [−0.65, −0.12]) for galantamine 24 mg, −0.62 (−1.01, −0.24) for galantamine 32 mg, −0.30 (−0.52, −0.07) for donepezil 10 mg, and −0.37 (−0.69, −0.04) for donepezil 5 mg. Galantamine 32 mg was superior to rivastigmine 12 mg (SMD = −0.65, 95% CrI: [−0.17, −0.20]) and the rivastigmine 10 cm^2^ patch (SMD = −0.52, 95% CrI: [−1.06, −0.02]). However, for other interventions, there were no statistically significant differences. In addition, when compared with rivastigmine 12 mg, galantamine 24 mg was more efficacious (SMD = −0.41, 95% CrI: [−0.85, −0.05]). The informative results for mild to moderate AD are shown in **Table 2** (in the top right corner). As shown in **Figure 4A** and **Additional file: Supplementary 5**, the five most efficient interventions were ranked as galantamine 32 mg (SUCRA = 93.2%), galantamine 24 mg (SUCRA = 75.5%), donepezil 5 mg (SUCRA = 73.3%), donepezil 10 mg (SUCRA = 65.6%), and memantine 20 mg (SUCRA = 57.0%). Furthermore, we also assessed cognitive function using the MMSE. The network plot, including a total of 17 studies, is presented in **Figure 3B**. We noted consistent results in both direct and indirect comparisons. In the network meta-analysis, no interventions were associated with statistically significant differences compared with placebo (**Figure 5A**). Furthermore, rivastigmine 12 mg had the highest probability of being ranked first according to SUCRA (72.9%), followed closely by the combination of the rivastigmine 10 cm^2^ patch and memantine 20 mg (SUCRA = 63.1%) and the rivastigmine 5 cm^2^ patch (SUCRA = 60.7%) (**Additional file: Supplementary 5**).

**Table 2:** Network meta-analysis comparison of 16 pharmacological treatments for mild to moderate Alzheimer’s disease.

PLA	0.03 (−0.24,0.36)	**−0.39 (−0.65,-0.12)**	−0.10 (−0.43,0.27)	**−0.30 (−0.52,-0.07)**	−0.16 (−0.74,0.42)	0.00 (−0.68,0.67)	−0.20 (−0.90,0.54)	0.07 (−0.51,0.66)	…	**−0.62 (−1.01,-0.24)**	−0.24 (−0.60,0.12)	−0.09 (−0.61,0.44)	…	**−0.37 (−0.69,-0.04)**	−0.20 (−0.85,0.49)
**0.52 (0.34,0.79)**	RIV12mg	**−0.41 (−0.85,-0.05)**	−0.13 (−0.49,0.21)	−0.33 (−0.72,0.01)	−0.19 (−0.88,0.43)	−0.03 (−0.80,0.68)	−0.22 (−0.97,0.48)	0.04 (−0.64,0.67)	…	**−0.65 (−0.17,-0.20)**	−0.26 (−0.76,0.17)	−0.12 (−0.70,0.42)	…	−0.40 (−0.87,0.01)	−0.23 (−0.92,0.42)
**0.72 (0.53,0.95)**	1.39 (0.82,2.28)	GAL24mg	0.29 (−0.14,0.74)	0.09 (−0.26,0.44)	0.22 (−0.42,0.87)	0.39 (−0.34,1.11)	0.19 (−0.57,0.97)	0.46 (−0.19,1.10)	…	−0.24 (−0.61,0.15)	0.15 (−0.29,0.59)	0.30 (−0.29,0.89)	…	0.02 (−0.40,0.44)	0.18 (−0.52,0.93)
**0.60 (0.37,0.95)**	1.15 (0.72,1.83)	0.83 (0.48,1.45)	RIV10cm^2^	−0.20 (−0.63,0.19)	−0.06 (−0.76,0.60)	0.09 (−0.68,0.85)	−0.10 (−0.73,0.52)	0.17 (−0.53,0.84)	…	**−0.52 (−1.06,-0.02)**	−0.14 (−0.66,0.35)	0.01 (−0.53,0.52)	…	−0.27 (−0.76,0.20)	−0.10 (−0.68,0.47)
0.82 (0.63,1.07)	1.59 (0.97,2.60)	1.14 (0.80,1.68)	1.38 (0.80,2.39)	DON10mg	0.14 (−0.49,0.76)	0.30 (−0.41,1.01)	0.10 (−0.63,0.87)	0.37 (−0.26,0.99)	…	−0.32 (−0.77,0.12)	0.06 (−0.32,0.44)	0.21 (−0.35,0.78)	…	−0.07 (−0.41,0.28)	0.10 (−0.59,0.83)
0.54 (0.17,1.62)	1.04 (0.31,3.40)	0.75 (0.23,2.35)	0.90 (0.26,3.03)	0.65 (0.20,2.04)	HupA400µg	0.16 (−0.52,0.83)	−0.04 (−0.95,0.91)	0.23 (−0.59,1.06)	…	−0.46 (−1.16,0.24)	−0.08 (−0.76,0.61)	0.07 (−0.71,0.86)	…	−0.21 (−0.88,0.47)	−0.04 (−0.91,0.87)
0.82 (0.24,2.66)	1.58 (0.44,5.41)	1.14 (0.33,3.85)	1.38 (0.38,4.94)	1.00 (0.29,3.35)	1.52 (0.52,4.75)	HupA200µg	−0.20 (−1.16,0.81)	0.07 (−0.82,0.97)	…	−0.62 (−1.40,0.15)	−0.24 (−1.00,0.52)	−0.09 (−0.95,0.77)	…	−0.37 (−1.12,0.38)	−0.20 (−1.14,0.76)
0.84 (0.26,2.81)	1.63 (0.51,5.37)	1.17 (0.35,4.05)	1.42 (0.49,4.22)	1.03 (0.31,3.53)	1.58 (0.31,8.34)	1.03 (0.20,5.74)	RIV10cm^2^+ MEM20mg	0.27 (−0.68,1.19)	…	−0.43 (−1.25,0.38)	−0.04 (−0.86,0.75)	0.11 (−0.72,0.92)	…	−0.17 (−0.97,0.61)	−0.01 (−0.87,0.84)
1.53 (0.71,3.35)	**2.95 (1.24,7.30)**	2.12 (0.95,5.00)	**2.57 (1.07,6.50)**	1.87 (0.85,4.19)	2.86 (0.74,11.84)	1.87 (0.46,8.11)	1.81 (0.45,7.45)	EGb240mg	…	−0.69 (−1.40,0.01)	−0.31 (−1.00,0.38)	−0.16 (−0.95,0.63)	…	−0.44 (−1.11,0.23)	−0.27 (−1.15,0.64)
0.87 (0.17,5.50)	1.68 (0.31,11.08)	1.22 (0.23,7.77)	1.47 (0.27,9.86)	1.06 (0.21,6.66)	1.63 (0.22,15.03)	1.07 (0.14,10.07)	1.05 (0.13,9.39)	0.57 (0.10,3.72)	EGb240mg+ DON10mg	…	…	…	…	…	…
**0.44 (0.27,0.71)**	0.84 (0.43-1.59)	**0.61 (0.37,0.99)**	0.74 (0.37,1.45)	0.53 (0.31,0.92)	0.82 (0.24,2.82)	0.53 (0.15,1.94)	0.51 (0.14,1.83)	**0.29 (0.11,0.70)**	0.50 (0.07,2.83)	GAL32mg	0.38 (−0.14,0.91)	0.53 (−0.11,1.19)	…	0.25 (−0.25,0.75)	0.42 (−0.33,1.21)
0.76 (0.42,1.38)	1.48 (0.70,3.05)	1.06 (0.55,2.06)	1.29 (0.60,2.77)	0.93 (0.48,1.77)	1.42 (0.41,5.17)	0.93 (0.25,3.52)	0.90 (0.24,3.36)	0.50 (0.18,1.31)	0.88 (0.12,5.05)	1.75 (0.81,3.75)	MEM20mg	0.15 (−0.48,0.79)	…	−0.13 (−0.59,0.34)	0.03 (−0.72,0.82)
0.58 (0.29,1.18)	1.13 (0.53,2.40)	0.81 (0.39,1.75)	0.98 (0.49,1.97)	0.71 (0.34,1.50)	1.10 (0.30,4.13)	0.71 (0.18,2.85)	0.70 (0.19,2.49)	0.38 (0.13,1.07)	0.66 (0.09,4.05)	1.34 (0.58,3.16)	0.77 (0.31,1.92)	RIV5cm^2^	…	−0.28 (−0.90,0.33)	−0.11 (−0.89,0.68)
1.26 (0.34,5.35)	2.44 (0.61,10.99)	1.75 (0.46,7.66)	2.12 (0.53,9.76)	1.53 (0.41,6.60)	2.35 (0.43,14.79)	1.53 (0.26,10.43)	1.48 (0.25,9.82)	0.82 (0.18,4.15)	1.42 (0.15,13.33)	2.87 (0.71,13.38)	1.65 (0.39,7.90)	2.15 (0.49,10.78)	EGb160mg	…	…
1.26 (0.83,1.96)	**2.44 (1.34,4.47)**	**1.75 (1.06,3.00)**	**2.12 (1.13,4.07)**	**1.54 (1.01,2.37)**	2.36 (0.72,7.94)	1.54 (0.44,5.59)	1.50 (0.42,5.16)	0.82 (0.34,2.00)	1.44 (0.22,7.88)	**2.89 (1.53,5.62)**	1.65 (0.81,3.54)	2.16 (0.96,4.91)	1.01 (0.24,3.73)	DON5mg	0.16 (−0.57,0.93)
0.70 (0.29,1.67)	1.35 (0.56,3.20)	0.97 (0.38,2.45)	1.17 (0.55,2.48)	0.85 (0.33,2.11)	1.31 (0.31,5.54)	0.85 (0.19,3.85)	0.83 (0.22,3.04)	0.46 (0.14,1.43)	0.80 (0.10,5.08)	1.60 (0.57,4.35)	0.91 (0.32,2.67)	1.20 (0.43,3.29)	0.56 (0.10,2.69)	0.55 (0.20,1.45)	RIV15cm^2^

The results are presented as the OR and 95% CrI for acceptability (lower left quarter) and as the SMD and 95% CrI for cognitive function in ADAS-cog (upper right quarter). For acceptability, ORs higher than 1 favor the column-defining treatment. For cognitive function, SMDs lower than 1 favor the row-defining treatment. OR, odds ratio; CrI, credible interval; SMD, standard mean difference; ADAS-cog, Alzheimer’s Disease Assessment Scale-cognition subscale; PLA, placebo; RIV12mg, Rivastigmine 12 mg; GAL24mg, Galantamine 24 mg; RIV10cm2, Rivastigmine patch 10 cm^2^; DON10mg, Donepezil 10 mg; HupA400µg, Huperzine A 400 µg; HupA200µg, Huperzine A 200 µg; RIV10cm2+MEM20mg, Rivastigmine patch 10 cm^2^ + Memantine 20 mg; EGb240mg, EGb761 240 mg; EGb240mg+DON10mg, EGb761 240 mg + Donepezil 10 mg; GAL32mg, Galantamine 32 mg; MEM20mg, Memantine 20 mg; RIV5cm2, Rivastigmine patch 5 cm^2^; EGb160mg, EGb761 160 mg; DON5mg, Donepezil 5 mg; RIV15cm2, Rivastigmine patch 15 cm^2^.

Bold texts in the table mean ORs and SMDs are statistically significant. Light blue in the lower left corner represented the odds ratio (OR) and 95% credible interval (CrI) for acceptability, orange in the upper right corner represented the standard mean difference (SMD) and 95% CrI for cognitive function in ADAS-cog, and dark blue represented different interventions.

The network of eligible comparisons for the assessment of acceptability is shown in **Figure 3C**. In total, 33 trials and 16 treatments were included; most treatments were monotherapies, except for the combinations of EGb761 240 mg and donepezil 10 mg and the rivastigmine 10 cm^2^ patch and memantine 20 mg. We found no evidence indicating an inconsistency between direct and indirect evidence *via* the IF and 95% CIs of nine closed loops (**Additional file: Supplementary 4**). Our analysis showed that the interventions of rivastigmine 12 mg (OR = 0.52, 95% CrI: [0.34, 0.79]), galantamine 24 mg (OR = 0.72, 95% CrI: [0.53, 0.95]), rivastigmine 10 cm^2^ patch (OR = 0.60, 95% CrI: [0.37, 0.95]), and galantamine 32 mg (OR = 0.44, 95% CrI: [0.27, 0.71]) were associated with a significantly increased probability of treatment completion compared with placebo. In addition, EGb761 240 mg was superior to the rivastigmine 10 cm^2^ patch (OR = 2.57, 95% CrI: [1.07, 6.50]) and rivastigmine 12 mg (OR = 2.95, 95% CrI: [1.24, 7.30]). Moreover, galantamine 32 mg was inferior to EGb761 240 mg (OR = 0.29, 95% CrI: [0.11,0.70]) (see the left corner of **Table 2**). We also ranked all treatments and found that EGb761 240 mg (SUCRA = 87.5%), donepezil 5 mg (SUCRA = 83.4%), and EGb761 160 mg (SUCRA = 72.5%) were most likely to be ranked first (**Figure 4B**).

A total of 32 trials with 13 interventions presented data on adverse events. The network diagram is presented in **Figure 3D**. The direct and indirect evidence was consistent (**Additional file: Supplementary 4**). Our network meta-analysis demonstrated that only EGb761 240 mg was better tolerated than placebo for safety (OR = 0.66, 95% CrI: [0.43, 0.99]). Rivastigmine 12 mg, galantamine 24 mg, the rivastigmine 10 cm^2^ patch, donepezil 10 mg, galantamine 32 mg, and the rivastigmine 15 cm^2^ patch were associated with a significantly increased risk of adverse events compared with placebo (OR = 2.69, 95% CrI: [1.96, 3.90], OR = 1.53, 95% CrI: [1.25, 1.98], OR = 1.68, 95% CrI: [1.18, 2.48], OR = 1.43, 95% CrI: [1.16, 1.76], OR = 2.51, 95% CrI: [1.58, 4.09], OR = 2.34, 95% CrI: [1.19, 4.83], respectively; **Figure 5B**). Other drugs, such as donepezil 5 mg as a monotherapy, and the combinations of the rivastigmine 10 cm^2^ patch with memantine 20 mg as well as EGb761 240 mg with donepezil 10 mg showed no statistical differences when compared with placebo. Based on SUCRA values, the optimal acceptable intervention was likely to be EGb761 240 mg (SUCRA = 97.8%). Memantine 20 mg and donepezil 5 mg followed closely behind as the second (SUCRA = 78.9%) and third (SUCRA = 71.7%) most acceptable interventions (**Additional file: Supplementary 5**).

### Network Meta-Analysis—Secondary Outcomes

Networks of eligible comparisons of the secondary outcomes are presented in **Additional file: Supplementary 6**, demonstrating predominantly head-to-head comparisons of drugs with active drugs or placebo. Regardless of whether the CIBIC-plus scale, ADCS-ADL, or NPI scales were used, the direct and indirect evidence indicated consistent results. (**Additional file: Supplementary 4**). For the assessment of clinical global impressions *via* the CIBIC-plus scale, memantine 20 mg, donepezil 10 mg, rivastigmine 12 mg, and donepezil 5 mg were significantly superior to placebo (SMD = −0.27, 95% CrI: [−0.48, −0.07]; SMD = −0.34, 95% CrI: [−0.50, −0.17]; SMD = −0.40, 95% CrI: [−0.62, −0.18]; SMD = −0.29, 95% CrI: [−0.53, −0.06]) (**Additional file: Supplementary 7**). The SUCRAs ranged from 83.7% for the highest-ranked treatment strategy (rivastigmine 12 mg) to 40.3% for the lowest-ranked agent (rivastigmine 5 cm^2^) (**Additional file: Supplementary 5**). In the assessment for improvements daily living using the ADCS-ADL scale, donepezil 10 mg, galantamine 24 mg, and the rivastigmine 15 cm^2^ patch were statistically more efficacious than placebo, with SMDs and 95% CrIs of 0.21 (0.02, 0.40) for donepezil 10 mg, 0.22 (0.06, 0.37) for galantamine 24 mg, and 0.51 (0.17, 0.81) for the rivastigmine 15 cm^2^ patch (**Additional file: Supplementary 7**). As shown in **Additional file: Supplementary 5**, the rank of the three most efficient interventions was the rivastigmine 15 cm^2^ (SUCRA = 93.7%), the combination of rivastigmine 10 cm^2^ and memantine 20 mg (SUCRA = 71.1%), followed by galantamine 24 mg (SUCRA = 60.3%). Twelve studies assessed neuropsychiatric symptoms using the NPI scale for nine different treatment interventions and placebo. However, in our network meta-analysis, there were no interventions that significantly improved neuropsychiatric symptoms compared with placebo.

### Publication Bias

We produced comparison-adjusted funnel plots, with different colors representing different comparisons. Through a visual inspection, we found that the funnel plots presented an essentially symmetrical distribution, indicating that there were no small-sample effects for any outcomes (**Additional file: Supplementary 8**).

## Discussion

This comprehensive network meta-analysis was based on 37 trials, which included 14,705 patients with mild to moderate AD randomly assigned to currently available active agents or placebo, and compared the efficacy, acceptability, and safety of various regimens. The magnitude of intervention ranking varied enormously across different cognitive enhancers and doses, especially in different assessment outcomes. The results suggested that for patients with mild to moderate AD, galantamine 32 mg, galantamine 24 mg, donepezil 5 mg, donepezil 10 mg, and memantine 20 mg were more efficacious for cognitive improvements than other pharmacotherapies. The EGb761 240 mg treatment appeared to be the most optimal in terms of both acceptability and safety. Moreover, of the current treatment therapies, rivastigmine 12 mg offered a more favorable profile with benefits in the clinical global impression. The rivastigmine 15 cm^2^ patch, another rivastigmine dosage form, had the highest probability of functional improvement. However, we did not find any effective interventions resulting in behavioral improvements. This project extends a previous network meta-analysis that addressed ten interventions with data for direct and indirect comparisons (Dou et al., 2018). Our study can assist in the provision of relevant options for clinical pharmacotherapies for patients with mild to moderate AD.

Galantamine is a reversible and competitive AChEI (Bores et al., 1996). A previous meta-analysis concluded that galantamine was an effective therapeutic agent and was a preferred treatment for AD compared with donepezil, memantine, and rivastigmine (Li et al., 2019). Galantamine 32 mg was associated with a significant improvement in cognitive function; however, owing to poor acceptability and adverse events, its practical use may be limited. Based on the overall evidence, galantamine 24 mg may therefore, be the optimal treatment option for patients with mild to moderate AD. In addition, the major therapeutic effect of EGb761 240 mg is based on its acceptability and fewer associated adverse events. Although some studies have shown that EGb761 was favorable for cognitive, behavioral, and functional improvements, and clinical global impressions (Yancheva et al., 2009; Ihl et al., 2012; Yang et al., 2016), their sample sizes were much smaller, and the results were mixed. Thus, we propose that EGb761 should be researched further in large-scale randomized controlled trials. It has been reported that huperzine A is a well-tolerated intervention leading to improvements in cognitive impairment; however, until now, the evidence from our network meta-analysis did not recommend its use (Xing et al., 2014). A secondary analysis showed that regardless of dosage form and dose, rivastigmine produced a relatively marked improvement in both clinical global impression and daily living. The rivastigmine patch is frequently used in patients with mild to moderate AD because the adverse events associated with the patch are greatly reduced compared with that of the capsule form (Winblad et al., 2007). It is a novel drug delivery method that allows continuous drug administration.

We carefully monitored quality between the included trials and found that the majority of trials were considered to be unclear with regard to selection bias, especially, allocation concealment. Additionally, open-label trials were included. Nevertheless, our analysis could still be powered to provide objective evaluations for unclear factors given the even distribution of patient characteristics and the objective method adopted in each treatment group. Through the node-splitting method and loop-specific method, we noticed no significant differences between consistency in terms of the concerned evaluated outcomes. To assess the bias of small-sample effects, we also produced a comparison-adjusted funnel plot, and the findings were reassuring.

We are aware of three studies associated with AD that also integrated direct and indirect comparisons simultaneously in one network meta-analysis (Dou et al., 2018; Thancharoen and Limwattananon, 2019; Tsoi et al., 2019). In contrast to these previous studies, our study included new interventions and integrated all available high-quality RCTs with regard to the effectiveness, acceptability, and safety of cognitive enhancers in treating mild to moderate AD in one analysis, while examining different doses of treatments as independent interventions.

As with any network meta-analysis, our study has some limitations. Although we tried our best to include all eligible literature through comprehensive and systematic review, the sample size was still small for some interventions in individual RCTs. Furthermore, not all studies reported data for each outcome measure. However, it is essential to include all eligible studies in a network meta-analysis to reduce potential biases. Finally, this study primarily compared the efficacy, acceptability, and safety of pharmacological treatments for mild to moderate AD but did not include an analysis of cost-effectiveness. It is known that AD poses an enormous economic burden, and it is necessary to consider the balance of the therapeutic effects and costs. However, there was a lack of primary data involving cost-effectiveness in the included studies.

## Conclusions

In summary, our network meta-analysis findings suggested that galantamine (32 mg and 24 mg) and donepezil (5 mg and 10 mg) were the most effective strategies for improving the cognitive symptoms of patients with mild to moderate AD. We posit our findings, which we believe can support clinical decision-making. When taking acceptability and safety into account, EGb761 240 mg may be the optimal therapeutic choice. Rivastigmine 12 mg achieved the highest level of clinical global impression, and in terms of function, rivastigmine 15 cm^2^ patch is likely to be the best intervention. Nevertheless, none of the interventions effectively improved behavior. We hope that our study contributes markedly to the process of making accurate and efficient clinical decisions with regard to AD treatment.

## Data Availability Statement

All datasets for this study are included in the **Supplementary Material**.

## Author Contributions

TZ and HL were involved in the concept and design of the study. TZ drafted the manuscript. All authors were involved in acquisition, analysis, and interpretation of the data, revised the manuscript, and approved the final version.

## Funding

This research was supported by the National Science and Technology Major Project for “Essential new drug research and development” (No. 2019ZX09301114), the National Natural Science Foundation of China (NO. 81873350) and the Beijing Natural Science Foundation (NO. 7202174).

## Conflict of Interest

The authors declare that the research was conducted in the absence of any commercial or financial relationships that could be construed as a potential conflict of interest.

## Abbreviations

AD, Alzheimer’s disease; AChEIs, acetylcholinesterase inhibitors; PRISRM, Preferred Reporting Items for Systematic Reviews and Meta-Analysis; RCTs, randomized controlled trials; DSM, Diagnostic and Statistical Manual of Mental Disorders; NINCDS-ADRDA, National Institute of Neurological and Communicative Disorders and Stroke and the Alzheimer’s Disease and Related Disorders Association; MMSE, minimental state examination; ADAS-cog, Alzheimer’s Disease Assessment Scale-cognition subscale; ADCS-ADL, Alzheimer’s Disease Cooperative Study-Activities of Daily Living; NPI, Neuropsychiatric Inventory; CIBIC-Plus, Clinician’s Interview-Based Impression of Change plus Caregiver Input; SMD, standard mean difference; ORs, odds ratios; DIC, Deviance Information Criteria; SUCRA, surface under the cumulative ranking curve.
