# Supplementary material for: Different Doses of Pharmacological Treatments for Mild to Moderate Alzheimer’s Disease: A Bayesian Network Meta-Analysis
Source: Front Pharmacol. 2020 May 26;11:778. doi: 10.3389/fphar.2020.00778 (PMC7264393; doi:10.3389/fphar.2020.00778)
Supplement: Supplementary file 1 [file DataSheet_1.doc]

Supplementary 1

Relevant databases retrieval record

| Source: PubMed (Searched on: 19 September 2019) | | |
| --- | --- | --- |
| Search | Query | Items found |
| #125 | Search #36 AND #58 AND #124 | 920 |
| #124 | Search #68 OR #83 OR #93 OR #105 OR #120 OR #123 | 8267 |
| #123 | Search #121 OR #122 | 393 |
| #122 | Search " huperzine A, (5alpha,9beta,11Z)-(-)-isomer"[Title/Abstract] | 0 |
| #121 | Search "huperzine A" [Supplementary Concept] | 393 |
| #120 | Search #106 OR #107 OR #108 OR #109 OR #110 OR #111 OR #112 OR #113 OR #114 OR #115 OR #116 OR #117 OR #118 OR #119 | 1196 |
| #119 | Search EGb761[Title/Abstract] | 207 |
| #118 | Search "EGb-761"[Title/Abstract] | 632 |
| #117 | Search "GBE 761 ONC"[Title/Abstract] | 3 |
| #116 | Search "EGb 761"[Title/Abstract] | 632 |
| #115 | Search Tebonin[Title/Abstract] | 40 |
| #114 | Search Tebokan[Title/Abstract] | 4 |
| #113 | Search Tebofortran[Title/Abstract] | 0 |
| #112 | Search "GBE-761"[Title/Abstract] | 7 |
| #111 | Search "Ginkgo biloba extract 761"[Title/Abstract] | 19 |
| #110 | Search "GBE 761"[Title/Abstract] | 7 |
| #109 | Search Tanakan[Title/Abstract] | 70 |
| #108 | Search Rokan[Title/Abstract] | 13 |
| #107 | Search "Ginkgo leaf extract"[Title/Abstract] | 26 |
| #106 | Search "Ginkgo biloba extract" [Supplementary Concept] | 944 |
| #105 | Search #94 OR #95 OR #96 OR #97 OR #98 OR #99 OR #100 OR #101 OR #102 OR #103 OR #104 | 2301 |
| #104 | Search D145[Title/Abstract] | 26 |
| #103 | Search "D 145"[Title/Abstract] | 53 |
| #102 | Search "D-145"[Title/Abstract] | 53 |
| #101 | Search Axura[Title/Abstract] | 9 |
| #100 | Search "Memantine Hydrochloride"[Title/Abstract] | 62 |
| #99 | Search Ebixa[Title/Abstract] | 22 |
| #98 | Search Namenda[Title/Abstract] | 30 |
| #97 | Search "1-Amino-3,5-dimethyladamantane"[Title/Abstract] | 38 |
| #96 | Search "1,3-Dimethyl-5-aminoadamantane"[Title/Abstract] | 19 |
| #95 | Search Memantin[Title/Abstract] | 19 |
| #94 | Search "Memantine"[Mesh] | 2187 |
| #93 | Search #84 OR #85 OR #86 OR #87 OR #88 OR #89 OR #90 OR #91 OR #92 | 1770 |
| #92 | Search "Galanthamine Hydrobromide"[Title/Abstract] | 35 |
| #91 | Search Reminyl[Title/Abstract] | 59 |
| #90 | Search Razadyne[Title/Abstract] | 11 |
| #89 | Search Nivalin[Title/Abstract] | 46 |
| #88 | Search Nivaline[Title/Abstract] | 64 |
| #87 | Search Galanthamine[Title/Abstract] | 617 |
| #86 | Search Galantamin[Title/Abstract] | 14 |
| #85 | Search Lycoremine[Title/Abstract] | 2 |
| #84 | Search "Galantamine"[Mesh] | 1514 |
| #83 | Search #69 OR #70 OR #71 OR #72 OR #73 OR #74 OR #75 OR #76 OR #77 OR #78 OR #79 OR #80 OR #81 OR #82 | 1125 |
| #82 | Search ENA713[Title/Abstract] | 5 |
| #81 | Search "ENA-713"[Title/Abstract] | 43 |
| #80 | Search "ENA 713, SDZ"[Title/Abstract] | 0 |
| #79 | Search "713, SDZ ENA"[Title/Abstract] | 0 |
| #78 | Search "SDZ ENA 713"[Title/Abstract] | 20 |
| #77 | Search "713, ENA"[Title/Abstract] | 0 |
| #76 | Search "ENA 713"[Title/Abstract] | 43 |
| #75 | Search Exelon[Title/Abstract] | 81 |
| #74 | Search "Tartrate, Rivastigmine Hydrogen"[Title/Abstract] | 0 |
| #73 | Search "Hydrogen Tartrate, Rivastigmine"[Title/Abstract] | 0 |
| #72 | Search "Rivastigmine Hydrogen Tartrate"[Title/Abstract] | 12 |
| #71 | Search "RivastigmineTartrate"[Title/Abstract] | 0 |
| #70 | Search "(S)-N-Ethyl-3-((1-dimethyl-amino)ethyl)-N-methylphenylcarbamate"[Title/Abstract] | 0 |
| #69 | Search "Rivastigmine"[Mesh] | 1096 |
| #68 | Search #59 OR #60 OR #61 OR #62 OR #63 OR #64 OR #65 OR #66 OR #67 | 2534 |
| #67 | Search Aricept[Title/Abstract] | 153 |
| #66 | Search "1-Benzyl-4-((5,6-dimethoxy-1-indanon)-2-yl)methylpiperidine hydrochloride"[Title/Abstract] | 3 |
| #65 | Search "Donepezil Hydrochloride"[Title/Abstract] | 187 |
| #64 | Search "E-2020"[Title/Abstract] | 8 |
| #63 | Search "E2020"[Title/Abstract] | 80 |
| #62 | Search "E 2020"[Title/Abstract] | 8 |
| #61 | Search "Donepezilium Oxalate Trihydrate"[Title/Abstract] | 1 |
| #60 | Search Eranz[Title/Abstract] | 0 |
| #59 | Search "Donepezil"[Mesh] | 2403 |
| #58 | Search #37 OR #38 OR #39 OR #40 OR #41 OR #42 OR #43 OR #44 OR #45 OR #46 OR #47 OR #48 OR #49 OR #50 OR #51 OR #52 OR #53 OR #54 OR #55 OR #56 OR #57 | 1303742 |
| #57 | Search tripleblind*[Title/Abstract] | 3 |
| #56 | Search trebleblind*[Title/Abstract] | 0 |
| #55 | Search doubleblind*[Title/Abstract] | 236 |
| #54 | Search singleblind*[Title/Abstract] | 16 |
| #53 | Search blind*[Title/Abstract] | 285786 |
| #52 | Search random*[Title/Abstract] | 1075621 |
| #51 | Search "Double-Blind Method"[Mesh] | 153412 |
| #50 | Search "Single-Blind Method"[Mesh] | 27339 |
| #49 | Search "Pragmatic Clinical Trials as Topic"[Publication Type] | 0 |
| #48 | Search "Randomized Controlled Trials"[Publication Type] | 0 |
| #47 | Search "Controlled Clinical Trials"[Publication Type] | 0 |
| #46 | Search "Clinical Trials, Phase IV"[Publication Type] | 0 |
| #45 | Search "Clinical Trials, Phase III"[Publication Type] | 0 |
| #44 | Search "Clinical Trials, Phase II"[Publication Type] | 0 |
| #43 | Search "Pragmatic Clinical Trials as Topic"[Mesh] | 342 |
| #42 | Search "Intention to Treat Analysis"[Mesh] | 2413 |
| #41 | Search "Randomized Controlled Trials as Topic"[Mesh] | 129418 |
| #40 | Search "Controlled Clinical Trials as Topic"[Mesh] | 134523 |
| #39 | Search "Clinical Trials, Phase IV as Topic"[Mesh] | 290 |
| #38 | Search "Clinical Trials, Phase III as Topic"[Mesh] | 8929 |
| #37 | Search "Clinical Trials, Phase II as Topic"[Mesh] | 7459 |
| #36 | Search #1 OR #2 OR #3 OR #4 OR #5 OR #6 OR #7 OR #8 OR #9 OR #10 OR #11 OR #12 OR #13 OR #14 OR #15 OR #16 OR #17 OR #18 OR #19 OR #20 OR #21 OR #22 OR #23 OR #24 OR #25 OR #26 OR #27 OR #28 OR #29 OR #30 OR #31 OR #32 OR #33 OR #34 OR #35 | 138930 |
| #35 | Search "Presenile Alzheimer Dementia"[Title/Abstract] | 3 |
| #34 | Search "Early Onset Alzheimer Disease"[Title/Abstract] | 149 |
| #33 | Search "Alzheimer Disease, Early Onset"[Title] | 0 |
| #32 | Search "Familial Alzheimer Diseases (FAD)"[Title/Abstract] | 0 |
| #31 | Search "Alzheimer Diseases, Familial (FAD)"[Title/Abstract] | 0 |
| #30 | Search "Alzheimer Disease, Familial (FAD)"[Title/Abstract] | 0 |
| #29 | Search "Familial Alzheimer Disease (FAD)"[Title/Abstract] | 78 |
| #28 | Search "Focal Onset Alzheimer's Disease"[Title/Abstract] | 0 |
| #27 | Search "Alzheimer's Disease, Focal Onset"[Title/Abstract] | 0 |
| #26 | Search "Late Onset Alzheimer Disease"[Title/Abstract] | 328 |
| #25 | Search "Alzheimer Disease, Late Onset"[Title/Abstract] | 0 |
| #24 | Search "Presenile Dementia"[Title/Abstract] | 573 |
| #23 | Search "Dementia, Presenile"[Title/Abstract] | 0 |
| #22 | Search "Senile Dementia, Acute Confusional"[Title/Abstract] | 0 |
| #21 | Search "Acute Confusional Senile Dementia"[Title/Abstract] | 0 |
| #20 | Search "Senile Dementia, Alzheimer Type"[Title/Abstract] | 0 |
| #19 | Search "Dementias, Alzheimer"[Title/Abstract] | 0 |
| #18 | Search "Dementia, Alzheimer"[Title/Abstract] | 1 |
| #17 | Search "Alzheimer Dementias"[Title/Abstract] | 50 |
| #16 | Search "Alzheimer Dementia"[Title/Abstract] | 617 |
| #15 | Search "Alzheimer Syndrome"[Title/Abstract] | 16 |
| #14 | Search "Sclerosis, Alzheimer"[Title/Abstract] | 1 |
| #13 | Search "Alzheimer Sclerosis"[Title/Abstract] | 0 |
| #12 | Search "Dementia, Primary Senile Degenerative"[Title/Abstract] | 0 |
| #11 | Search "Primary Senile Degenerative Dementia"[Title/Abstract] | 0 |
| #10 | Search "Alzheimer Type Senile Dementia"[Title/Abstract] | 21 |
| #9 | Search "Dementia, Alzheimer-Type (ATD)"[Title/Abstract] | 0 |
| #8 | Search "Alzheimer Type Dementia (ATD)"[Title/Abstract] | 117 |
| #7 | Search "Alzheimer-Type Dementia (ATD)"[Title/Abstract] | 117 |
| #6 | Search "Alzheimer Type Dementia"[Title/Abstract] | 715 |
| #5 | Search "Dementia, Alzheimer Type"[Title/Abstract] | 0 |
| #4 | Search "Senile Dementia"[Title/Abstract] | 2839 |
| #3 | Search "Dementia, Senile"[Title/Abstract] | 1 |
| #2 | Search "Alzheimer's Disease"[Title/Abstract] | 111097 |
| #1 | Search "Alzheimer Disease"[Mesh] | 89263 |

| Source: EMBASE (Searched on: 19 September 2019) | | |
| --- | --- | --- |
| No. | Query | Results |
| #176 | #20 AND #160 AND #175 | 2156 |
| #175 | #161 OR #162 OR #163 OR #164 OR #165 OR #166 OR #167 OR #168 OR #169 OR #170 OR #171 OR #172 OR #173 OR #174 | 1787027 |
| #174 | tripleblind*:ab,ti | 7 |
| #173 | trebleblind*:ab,ti | 0 |
| #172 | doubleblind*:ab,ti | 3124 |
| #171 | singleblind*:ab,ti | 245 |
| #170 | blind*:ab,ti | 404258 |
| #169 | random*:ab,ti | 1446483 |
| #168 | 'double blind procedure'/exp | 164893 |
| #167 | 'single blind procedure'/exp | 36310 |
| #166 | 'randomized controlled trial (topic)'/exp | 166275 |
| #165 | 'controlled clinical trial (topic)'/exp | 173063 |
| #164 | 'phase 4 clinical trial (topic)'/exp | 1532 |
| #163 | 'phase 3 clinical trial (topic)'/exp | 34989 |
| #162 | 'phase 2 clinical trial (topic)'/exp | 33622 |
| #161 | 'multicenter study (topic)'/exp | 29067 |
| #160 | #42 OR #63 OR #77 OR #139 OR #142 OR #159 | 30501 |
| #159 | #143 OR #144 OR #145 OR #146 OR #147 OR #148 OR #149 OR #150 OR #151 OR #152 OR #153 OR #154 OR #155 OR #156 OR #157 OR #158 | 7277 |
| #158 | tebonin:ab,ti | 57 |
| #157 | tanakene:ab,ti | 0 |
| #156 | tanakan:ab,ti | 88 |
| #155 | superginkgo:ab,ti | 0 |
| #154 | rokan:ab,ti | 18 |
| #153 | 'li 1370':ab,ti | 20 |
| #152 | 'kaveri forte':ab,ti | 1 |
| #151 | kaveri:ab,ti | 28 |
| #150 | ginkor:ab,ti | 16 |
| #149 | ginkobene:ab,ti | 1 |
| #148 | ginkgopower:ab,ti | 0 |
| #147 | ginkgold:ab,ti | 4 |
| #146 | 'gingko biloba extract':ab,ti | 98 |
| #145 | egb761:ab,ti | 310 |
| #144 | 'egb 761':ab,ti | 793 |
| #143 | 'ginkgo biloba extract'/exp | 7042 |
| #142 | #140 OR #141 | 1639 |
| #141 | cerebra:ab,ti | 395 |
| #140 | 'huperzine a'/exp | 1305 |
| #139 | #78 OR #79 OR #80 OR #81 OR #82 OR #83 OR #84 OR #85 OR #86 OR #87 OR #88 OR #89 OR #90 OR #91 OR #92 OR #93 OR #94 OR #95 OR #96 OR #97 OR #98 OR #99 OR #100 OR #101 OR #102 OR #103 OR #104 OR #105 OR #106 OR #107 OR #108 OR #109 OR #110 OR #111 OR #112 OR #113 OR #114 OR #115 OR #116 OR #117 OR #118 OR #119 OR #120 OR #121 OR #122 OR #123 OR #124 OR #125 OR #126 OR #127 OR #128 OR #129 OR #130 OR #131 OR #132 OR #133 OR #134 OR #135 OR #136 OR #137 OR #138 | 7436 |
| #138 | zoroflog:ab,ti | 0 |
| #137 | zentan:ab,ti | 0 |
| #136 | 'vertusal sr':ab,ti | 0 |
| #135 | vertusal:ab,ti | 0 |
| #134 | 'spegal sr':ab,ti | 0 |
| #133 | spegal:ab,ti | 0 |
| #132 | 'reminyl xl':ab,ti | 0 |
| #131 | reminyl:ab,ti | 92 |
| #130 | 'razadyne er':ab,ti | 5 |
| #129 | razadyne:ab,ti | 21 |
| #128 | nivaline:ab,ti | 62 |
| #127 | 'nivalin p':ab,ti | 8 |
| #126 | nivalin:ab,ti | 69 |
| #125 | 'natagal sr':ab,ti | 0 |
| #124 | natagal:ab,ti | 0 |
| #123 | micol:ab,ti AND galantamine:ab,ti | 0 |
| #122 | 'memoton life':ab,ti | 0 |
| #121 | 'memoton life':ab,ti | 0 |
| #120 | memo:ab,ti AND drug:ab,ti | 44 |
| #119 | 'masparen retard':ab,ti | 0 |
| #118 | masparen:ab,ti | 0 |
| #117 | margal:ab,ti | 2 |
| #116 | lycoremine:ab,ti | 2 |
| #115 | lycoremin:ab,ti | 0 |
| #114 | 'luventa xl':ab,ti | 0 |
| #113 | luventa:ab,ti | 0 |
| #112 | loxifren:ab,ti | 0 |
| #111 | 'lotprosin xl':ab,ti | 0 |
| #110 | lotprosin:ab,ti | 0 |
| #109 | jilkon:ab,ti | 0 |
| #108 | girlamen:ab,ti | 0 |
| #107 | gazylan:ab,ti | 0 |
| #106 | 'gatalin xl':ab,ti | 0 |
| #105 | gatalin:ab,ti | 0 |
| #104 | gamyl:ab,ti | 0 |
| #103 | 'galsya sr':ab,ti | 0 |
| #102 | galsya:ab,ti | 0 |
| #101 | galnora:ab,ti | 0 |
| #100 | 'galema sr':ab,ti | 0 |
| #99 | galema:ab,ti | 1 |
| #98 | galatamina:ab,ti | 0 |
| #97 | galatamin:ab,ti | 0 |
| #96 | galanyl:ab,ti | 0 |
| #95 | galanthen:ab,ti | 0 |
| #94 | 'galanthamine hydrobromide':ab,ti | 46 |
| #93 | galanthamine:ab,ti | 737 |
| #92 | galantex:ab,ti | 0 |
| #91 | 'galantamine hydrobromide':ab,ti | 78 |
| #90 | 'elmino xl':ab,ti | 0 |
| #89 | elmino:ab,ti | 0 |
| #88 | 'consion xl':ab,ti | 0 |
| #87 | consion:ab,ti | 0 |
| #86 | 'bergal sr':ab,ti | 0 |
| #85 | bergal:ab,ti | 0 |
| #84 | aneprosil:ab,ti | 0 |
| #83 | alenzo:ab,ti | 0 |
| #82 | 'acumor xl':ab,ti | 0 |
| #81 | acumor:ab,ti | 0 |
| #80 | '4a, 5, 9, 10, 11, 12 hexahydro 3 methoxy 11 methyl 6h benzofuro [3a, 3, 2 ef] [2] benzazepin 6 ol':ab,ti | 2 |
| #79 | '1, 2, 3, 4, 6, 7, 7a, 11c octahydro 9 methoxy 2 methylbenzofuro':ab,ti AND '4, 3, 2 e, f, g':ab,ti AND 2:ab,ti AND 'benzazocin 2 ol':ab,ti | 0 |
| #78 | 'galantamine'/exp | 7250 |
| #77 | #64 OR #65 OR #66 OR #67 OR #68 OR #69 OR #70 OR #71 OR #72 OR #73 OR #74 OR #75 OR #76 | 12410 |
| #76 | memorit:ab,ti | 1 |
| #75 | memac:ab,ti | 4 |
| #74 | eranz:ab,ti | 3 |
| #73 | e2020:ab,ti | 92 |
| #72 | 'e 2020':ab,ti | 19 |
| #71 | 'donepezil hydrochloride':ab,ti | 309 |
| #70 | 'doneliquid geriasan':ab,ti | 0 |
| #69 | asenta:ab,ti | 0 |
| #68 | 'aricept odt':ab,ti | 1 |
| #67 | 'aricept evess':ab,ti | 0 |
| #66 | aricept:ab,ti | 241 |
| #65 | 2:ab,ti AND '1 benzyl 4 piperidylmethyl':ab,ti AND '5, 6 dimethoxy 1 indanone':ab,ti | 0 |
| #64 | 'donepezil'/exp | 12361 |
| #63 | #43 OR #44 OR #45 OR #46 OR #47 OR #48 OR #49 OR #50 OR #51 OR #52 OR #53 OR #54 OR #55 OR #56 OR #57 OR #58 OR #59 OR #60 OR #61 OR #62 | 10390 |
| #62 | sdz212713:ab,ti | 0 |
| #61 | 'sdz212 713':ab,ti | 0 |
| #60 | 'sdz212 713':ab,ti | 0 |
| #59 | 'sdz ena713':ab,ti | 0 |
| #58 | 'sdz ena 713':ab,ti | 31 |
| #57 | 'sdz 212713':ab,ti | 0 |
| #56 | 'sdz 212-713':ab,ti | 3 |
| #55 | 'sdz 212 713':ab,ti | 3 |
| #54 | 'rivastigmine tartrate':ab,ti | 33 |
| #53 | 'rivastigmine hydrogen tartrate':ab,ti | 23 |
| #52 | rivastigmin:ab,ti | 36 |
| #51 | prometax:ab,ti | 3 |
| #50 | nimvastid:ab,ti | 0 |
| #49 | 'n ethyl n methylcarbamic acid [3':ab,ti AND '1 dimethylaminoethyl':ab,ti AND 'phenyl] ester':ab,ti | 0 |
| #48 | 'n ethyl 3 [1':ab,ti AND dimethylamino:ab,ti AND 'ethyl] n methylphenylcarbamate hydrogen tartrate':ab,ti | 1 |
| #47 | 'exelon patch':ab,ti | 23 |
| #46 | exelon:ab,ti | 160 |
| #45 | ena713:ab,ti | 6 |
| #44 | 'ena 713':ab,ti | 56 |
| #43 | 'memantine'/exp | 10142 |
| #42 | #21 OR #22 OR #23 OR #24 OR #25 OR #26 OR #27 OR #28 OR #29 OR #30 OR #31 OR #32 OR #33 OR #34 OR #35 OR #36 OR #37 OR #38 OR #39 OR #40 OR #41 | 10229 |
| #41 | namenda:ab,ti | 49 |
| #40 | 'memantine hydrochloride':ab,ti | 118 |
| #39 | 'memantine hcl':ab,ti | 19 |
| #38 | maruxa:ab,ti | 0 |
| #37 | marixino:ab,ti | 0 |
| #36 | ebixza:ab,ti | 0 |
| #35 | ebixa:ab,ti | 35 |
| #34 | ebix:ab,ti | 5 |
| #33 | d145:ab,ti | 28 |
| #32 | 'd 145':ab,ti | 79 |
| #31 | axura:ab,ti | 13 |
| #30 | akatinol:ab,ti | 27 |
| #29 | 'adamantane, 5 amino 1, 3 dimethyl':ab,ti | 0 |
| #28 | '5 amino 1, 3 dimethyladamantane':ab,ti | 0 |
| #27 | '3, 5 dimethylaminoadamantane':ab,ti | 2 |
| #26 | '3, 5 dimethyl 1 adamantanamine':ab,ti | 8 |
| #25 | '3, 5 dimethyl 1 adamantamine':ab,ti | 0 |
| #24 | '1, 3 dimethyl 5 aminoadamantane':ab,ti | 21 |
| #23 | '1, 3 dimethyl 5 adamantanamine':ab,ti | 2 |
| #22 | '1 amino 3, 5 dimethyladamantane':ab,ti | 45 |
| #21 | 'memantine'/exp | 10142 |
| #20 | #1 OR #2 OR #3 OR #4 OR #5 OR #6 OR #7 OR #8 OR #9 OR #10 OR #11 OR #12 OR #13 OR #14 OR #15 OR #16 OR #17 OR #18 OR #19 | 188167 |
| #19 | 'late onset alzheimer disease':ab,ti | 444 |
| #18 | 'diffuse cortical sclerosis':ab,ti | 0 |
| #17 | 'dementia, alzheimer':ab,ti | 1043 |
| #16 | 'cortical sclerosis, diffuse':ab,ti | 0 |
| #15 | 'alzheimers disease':ab,ti | 1406 |
| #14 | 'alzheimers disease':ab,ti | 1406 |
| #13 | 'alzheimer syndrome':ab,ti | 20 |
| #12 | 'alzheimer sclerosis':ab,ti | 0 |
| #11 | 'alzheimer perusini disease':ab,ti | 9 |
| #10 | 'alzheimer neuron degeneration':ab,ti | 0 |
| #9 | 'alzheimer neurofibrillary degeneration':ab,ti | 23 |
| #8 | 'alzheimer neurofibrillary change':ab,ti | 4 |
| #7 | 'alzheimer fibrillary lesion':ab,ti | 0 |
| #6 | 'alzheimer fibrillary change':ab,ti | 0 |
| #5 | 'alzheimer dementia':ab,ti | 851 |
| #4 | 'alzeimers disease':ab,ti | 1 |
| #3 | 'alzeimers disease':ab,ti | 1 |
| #2 | 'alzeimer disease':ab,ti | 4 |
| #1 | 'alzheimer disease'/exp | 187601 |

| Source: Cochrane Library (Searched on: 19 September 2019) | |
| --- | --- |
| ID | Search |
| #1 | MeSH descriptor: [Alzheimer Disease] explode all trees |
| #2 | ("Senile Dementia, Acute Confusional"):ti,ab,kw (Word variations have been searched) |
| #3 | ("Acute Confusional Senile Dementia"):ti,ab,kw (Word variations have been searched) |
| #4 | ("Alzheimer Type Dementia (ATD)"):ti,ab,kw (Word variations have been searched) |
| #5 | ("Alzheimer Dementias"):ti,ab,kw (Word variations have been searched) |
| #6 | ("Dementia, Senile"):ti,ab,kw (Word variations have been searched) |
| #7 | ("Alzheimer Type Dementia"):ti,ab,kw (Word variations have been searched) |
| #8 | ("Primary Senile Degenerative Dementia"):ti,ab,kw (Word variations have been searched) |
| #9 | ("Dementia, Primary Senile Degenerative"):ti,ab,kw (Word variations have been searched) |
| #10 | ("Alzheimer Dementia"):ti,ab,kw (Word variations have been searched) |
| #11 | ("Alzheimer Sclerosis"):ti,ab,kw (Word variations have been searched) |
| #12 | ("Alzheimer's Disease"):ti,ab,kw (Word variations have been searched) |
| #13 | ("Dementias, Alzheimer"):ti,ab,kw (Word variations have been searched) |
| #14 | ("Alzheimer Syndrome"):ti,ab,kw (Word variations have been searched) |
| #15 | ("Alzheimer Type Senile Dementia"):ti,ab,kw (Word variations have been searched) |
| #16 | ("Sclerosis, Alzheimer"):ti,ab,kw (Word variations have been searched) |
| #17 | ("Dementia, Alzheimer"):ti,ab,kw (Word variations have been searched) |
| #18 | ("Dementia, Alzheimer-Type (ATD)"):ti,ab,kw (Word variations have been searched) |
| #19 | ("Alzheimer-Type Dementia (ATD)"):ti,ab,kw (Word variations have been searched) |
| #20 | ("Senile Dementia"):ti,ab,kw (Word variations have been searched) |
| #21 | ("Dementia, Alzheimer Type"):ti,ab,kw (Word variations have been searched) |
| #22 | ("Senile Dementia, Alzheimer Type"):ti,ab,kw (Word variations have been searched) |
| #23 | ("Presenile Alzheimer Dementia"):ti,ab,kw (Word variations have been searched) |
| #24 | ("Early Onset Alzheimer Disease"):ti,ab,kw (Word variations have been searched) |
| #25 | ("Alzheimer Disease, Early Onset"):ti,ab,kw (Word variations have been searched) |
| #26 | ("Presenile Dementia"):ti,ab,kw (Word variations have been searched) |
| #27 | ("Dementia, Presenile"):ti,ab,kw (Word variations have been searched) |
| #28 | ("Alzheimer Disease, Late Onset"):ti,ab,kw (Word variations have been searched) |
| #29 | ("Late Onset Alzheimer Disease"):ti,ab,kw (Word variations have been searched) |
| #30 | ("Alzheimer Disease, Familial (FAD)"):ti,ab,kw (Word variations have been searched) |
| #31 | ("Familial Alzheimer Disease (FAD)"):ti,ab,kw (Word variations have been searched) |
| #32 | ("Familial Alzheimer Diseases (FAD)"):ti,ab,kw (Word variations have been searched) |
| #33 | ("Alzheimer's Disease, Focal Onset"):ti,ab,kw (Word variations have been searched) |
| #34 | ("Focal Onset Alzheimer's Disease"):ti,ab,kw (Word variations have been searched) |
| #35 | ("Alzheimer Diseases, Familial (FAD)"):ti,ab,kw (Word variations have been searched) |
| #36 | #1 OR #2 OR #3 OR #4 OR #5 OR #6 OR #7 OR #8 OR #9 OR #10 OR #11 OR #12 OR #13 OR #14 OR #15 OR #16 OR #17 OR #18 OR #19 OR #20 OR #21 OR #22 OR #23 OR #24 OR #25 OR #26 OR #27 OR #28 OR #29 OR #30 OR #31 OR #32 OR #33 OR #34 OR #35 |
| #37 | MeSH descriptor: [Donepezil] explode all trees |
| #38 | ("E 2020"):ti,ab,kw (Word variations have been searched) |
| #39 | ("E-2020"):ti,ab,kw (Word variations have been searched) |
| #40 | ("E2020"):ti,ab,kw (Word variations have been searched) |
| #41 | ("Donepezilium Oxalate Trihydrate"):ti,ab,kw (Word variations have been searched) |
| #42 | ("Eranz"):ti,ab,kw (Word variations have been searched) |
| #43 | ("1-Benzyl-4-((5,6-dimethoxy-1-indanon)-2-yl)methylpiperidine hydrochloride"):ti,ab,kw (Word variations have been searched) |
| #44 | ("Donepezil Hydrochloride"):ti,ab,kw (Word variations have been searched) |
| #45 | ("Aricept"):ti,ab,kw (Word variations have been searched) |
| #46 | #37 OR #38 OR #39 OR #40 OR #41 OR #42 OR #43 OR #44 OR #45 |
| #47 | MeSH descriptor: [Galantamine] explode all trees |
| #48 | ("Nivaline"):ti,ab,kw (Word variations have been searched) |
| #49 | ("Nivalin"):ti,ab,kw (Word variations have been searched) |
| #50 | ("Galanthamine Hydrobromide"):ti,ab,kw (Word variations have been searched) |
| #51 | ("Reminyl"):ti,ab,kw (Word variations have been searched) |
| #52 | ("Razadyne"):ti,ab,kw (Word variations have been searched) |
| #53 | ("Lycoremine"):ti,ab,kw (Word variations have been searched) |
| #54 | ("Galantamin"):ti,ab,kw (Word variations have been searched) |
| #55 | ("Galanthamine"):ti,ab,kw (Word variations have been searched) |
| #56 | #47 OR #48 OR #49 OR #50 OR #51 OR #52 OR #53 OR #54 OR #55 |
| #57 | MeSH descriptor: [Rivastigmine] explode all trees |
| #58 | ("ENA-713"):ti,ab,kw (Word variations have been searched) |
| #59 | ("713, SDZ ENA"):ti,ab,kw (Word variations have been searched) |
| #60 | ("ENA 713, SDZ"):ti,ab,kw (Word variations have been searched) |
| #61 | ("ENA713"):ti,ab,kw (Word variations have been searched) |
| #62 | ("SDZ ENA 713"):ti,ab,kw (Word variations have been searched) |
| #63 | ("ENA 713"):ti,ab,kw (Word variations have been searched) |
| #64 | ("713, ENA"):ti,ab,kw (Word variations have been searched) |
| #65 | ("(S)-N-Ethyl-3-((1-dimethyl-amino)ethyl)-N-methylphenylcarbamate"):ti,ab,kw (Word variations have been searched) |
| #66 | ("Rivastigmine Hydrogen Tartrate"):ti,ab,kw (Word variations have been searched) |
| #67 | ("Tartrate, Rivastigmine Hydrogen"):ti,ab,kw (Word variations have been searched) |
| #68 | ("RivastigmineTartrate"):ti,ab,kw (Word variations have been searched) |
| #69 | ("Hydrogen Tartrate, Rivastigmine"):ti,ab,kw (Word variations have been searched) |
| #70 | ("Exelon"):ti,ab,kw (Word variations have been searched) |
| #71 | #57 OR #58 OR #59 OR #60 OR #61 OR #62 OR #63 OR #64 OR #65 OR #66 OR #67 OR #68 OR #69 OR #70 |
| #72 | MeSH descriptor: [Memantine] explode all trees |
| #73 | ("Memantine Hydrochloride"):ti,ab,kw (Word variations have been searched) |
| #74 | ("D 145"):ti,ab,kw (Word variations have been searched) |
| #75 | ("D-145"):ti,ab,kw (Word variations have been searched) |
| #76 | ("D145"):ti,ab,kw (Word variations have been searched) |
| #77 | ("Ebixa"):ti,ab,kw (Word variations have been searched) |
| #78 | ("1-Amino-3,5-dimethyladamantane"):ti,ab,kw (Word variations have been searched) |
| #79 | ("Memantin"):ti,ab,kw (Word variations have been searched) |
| #80 | ("1,3-Dimethyl-5-aminoadamantane"):ti,ab,kw (Word variations have been searched) |
| #81 | ("Axura"):ti,ab,kw (Word variations have been searched) |
| #82 | ("Namenda"):ti,ab,kw (Word variations have been searched) |
| #83 | #72 OR #73 OR #74 OR #75 OR #76 OR #77 OR #78 OR #79 OR #80 OR #81 OR #82 |
| #84 | ("huperzine A"):ti,ab,kw (Word variations have been searched) |
| #85 | ("huperzine A, (5alpha,9beta,11Z)-(-)-isomer"):ti,ab,kw (Word variations have been searched) |
| #86 | #84 OR #85 |
| #87 | ("Ginkgo biloba extract"):ti,ab,kw (Word variations have been searched) |
| #88 | ("Ginkgo leaf extract"):ti,ab,kw (Word variations have been searched) |
| #89 | ("Rokan"):ti,ab,kw (Word variations have been searched) |
| #90 | ("Tanakan"):ti,ab,kw (Word variations have been searched) |
| #91 | ("GBE 761"):ti,ab,kw (Word variations have been searched) |
| #92 | ("Ginkgo biloba extract 761"):ti,ab,kw (Word variations have been searched) |
| #93 | ("GBE-761"):ti,ab,kw (Word variations have been searched) |
| #94 | ("Tebofortran"):ti,ab,kw (Word variations have been searched) |
| #95 | ("Tebokan"):ti,ab,kw (Word variations have been searched) |
| #96 | ("Tebonin"):ti,ab,kw (Word variations have been searched) |
| #97 | ("EGb 761"):ti,ab,kw (Word variations have been searched) |
| #98 | ("GBE 761 ONC"):ti,ab,kw (Word variations have been searched) |
| #99 | ("EGb-761"):ti,ab,kw (Word variations have been searched) |
| #100 | ("EGb761"):ti,ab,kw (Word variations have been searched) |
| #101 | #87 OR #88 OR #89 OR #90 OR #91 OR #92 OR #93 OR #94 OR #95 OR #96 OR #97 OR #98 OR #99 OR #100 |
| #102 | #46 OR #56 OR #71 OR #83 OR #86 OR #101 |
| #103 | #36 AND #102 |

Supplementary 2

Risk of bias summary


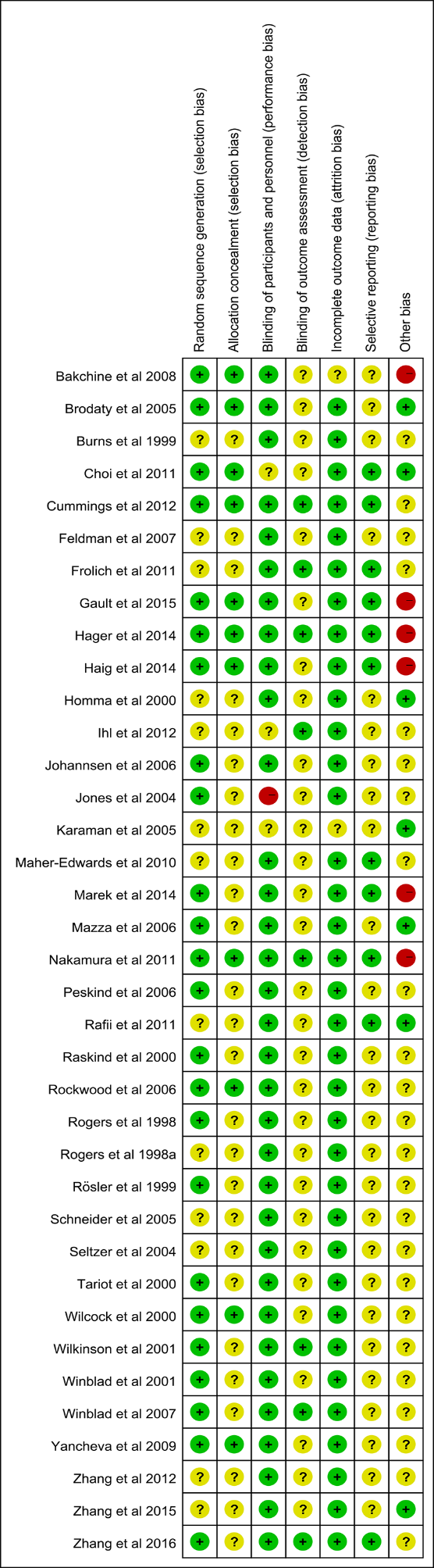


Supplementary 3

Pair-wise meta-analysis, Heterogeneity test, *I*² and heterogeneity estimate

| **Primary outcomes** | | | | | |
| --- | --- | --- | --- | --- | --- |
| **Efficacy on cognition (ADAS-cog) for mild to moderate AD** | | | | | |
| **Direct comparisons** | **No. of studies** | **SMD (95% CI)** | **P-value** | ***I*²** | **τ²** |
| DON10mg vs PLA | 7 | **-0.306 (-0.469, -0.142)** | 0.027 | 57.9% | 0.0273 |
| DON10mg vs MEM20mg | 1 | 0.011 (-0.294, 0.315) | - | - | - |
| DON5mg vs PLA | 3 | **-0.453 (-0.589, -0.317)** | 0.856 | 0.0% | 0.0000 |
| DON5mg vs DON10mg | 2 | 0.087 (-0.071, 0.246) | 0.758 | 0.0% | 0.0000 |
| RIV12mg vs PLA | 4 | 0.164 (-0.344, 0.673) | 0.000 | 94.9% | 0.2386 |
| RIV12mg vs RIV10cm^2^ | 2 | -0.016 (-0.142, 0.110) | 0.799 | 0.0% | 0.0000 |
| RIV10cm^2^ vs PLA | 2 | **-0.240 (-0.360, -0.119)** | 0.971 | 0.0% | 0.0000 |
| RIV10cm^2^ vs RIV5cm^2^ | 1 | -0.080 (-0.248, 0.088) | - | - | - |
| RIV10cm^2^ vs RIV15cm^2^ | 1 | -0.105 (-0.275, 0.065) | - | - | - |
| RIV10cm^2^ vs RIV10cm^2^+MEM20mg | 1 | 0.097 (-0.216, 0.409) | - | - | - |
| RIV5cm^2^ vs PLA | 1 | -0.160 (-0.329, 0.010) | - | - | - |
| GAL24mg vs PLA | 5 | **-0.387 (-0.577, -0.197)** | 0.001 | 78.0% | 0.0354 |
| GAL24mg vs GAL32mg | 2 | 0.315 (-0.207, 0.836) | 0.000 | 93.1% | 0.1318 |
| GAL32mg vs PLA | 2 | **-0.527 (-0.665, -0.389)** | 0.894 | 0.0% | 0.0000 |
| MEM20mg vs PLA | 2 | **-0.196 (-0.337, -0.056)** | 0.521 | 0.0% | 0.0000 |
| HupA400μg vs PLA | 1 | -0.302 (-0.634, 0.030) | - | - | - |
| HupA400μg vs HupA200μg | 1 | -0.139 (-0.474, 0.197) | - | - | - |
| HupA200μg vs PLA | 1 | 0.002 (-0.327, 0.331) | - | - | - |
| EGb240mg vs PLA | 1 | 0.072 (-0.139, 0.283) | - | - | - |
| **Efficacy on cognition (MMSE) for mild to moderate AD** | | | | | |
| **Direct comparisons** | **No. of studies** | **SMD (95% CI)** | **P-value** | ***I*²** | **τ²** |
| DON10mg vs PLA | 6 | **0.314 (0.206, 0.423)** | 0.710 | 0.0% | 0.0000 |
| DON10mg vs MEM20mg | 1 | -0.004 (-0.309, 0.300) | - | - | - |
| DON5mg vs PLA | 3 | **0.320 (0.167, 0.474)** | 0.944 | 0.0% | 0.0000 |
| DON5mg vs DON10mg | 2 | -0.070(-0.229, 0.088) | 0.729 | 0.0% | 0.0000 |
| RIV12mg vs PLA | 4 | -0.067(-0.513, 0.379) | 0.000 | 93.9% | 0.1753 |
| RIV12mg vs RIV10cm^2^ | 2 | -0.048(-0.173, 0.078) | 0.470 | 0.0% | 0.0000 |
| RIV10cm^2^ vs PLA | 2 | **0.215(0.002, 0.428)** | 0.083 | 66.7% | 0.0158 |
| RIV10cm^2^ vs RIV5cm^2^ | 1 | 0.101(-0.077,0.280) | - | - | - |
| RIV10cm^2^ vs RIV10cm^2^+MEM20mg | 1 | 0.140 (-0.173,0.453) | - | - | - |
| RIV5cm^2^ vs PLA | 1 | 0.000 (-0.178,0.178) | - | - | - |
| GAL24mg vs PLA | 1 | **0.174 (0.087,0.261)** | - | - | - |
| HupA400μg vs PLA | 1 | **0.485 (0.150,0.820)** | - | - | - |
| HupA400μg vs HupA200μg | 1 | 0.158 (-0.177,0.494) | - | - | - |
| HupA200μg vs PLA | 1 | **0.339 (0.008,0.671)** | - | - | - |
| EGb160mg vs PLA | 1 | 0.144 (-0.406,0.694) | - | - | - |
| EGb160mg vs DON5mg | 1 | -0.103 (-0.658,0.451) | - | - | - |
| **Acceptability for mild to moderate AD** | | | | | |
| **Direct comparisons** | **No. of studies** | **OR (95% CI)** | **P-value** | ***I*²** | **τ²** |
| DON10mg vs PLA | 11 | 1.198 (0.871, 1.648) | 0.006 | 59.7% | 0.1619 |
| DON10mg vs EGb240mg+DON10mg | 1 | 1.333 (0.271, 6.492) | - | - | - |
| DON10mg vs EGb240mg | 1 | 4.138 (0.436, 39.258) | - | - | - |
| DON5mg vs PLA | 4 | 0.991 (0.708, 1.386) | 0.338 | 11.0% | 0.0145 |
| DON5mg vs DON10mg | 3 | **0.552 (0.343, 0.891)** | 0.085 | 59.5% | 0.1056 |
| RIV12mg vs PLA | 3 | **1.964 (1.114, 3.463)** | 0.013 | 76.9% | 0.1928 |
| RIV12mg vs RIV10cm^2^ | 2 | 1.067 (0.800, 1.423) | 0.454 | 0.0% | 0.0000 |
| RIV10cm^2^ vs PLA | 2 | **1.668 (1.109, 2.509)** | 0.185 | 43.1% | 0.0374 |
| RIV10cm^2^ vs RIV5cm^2^ | 1 | 0.890 (0.597, 1.326) | - | - | - |
| RIV10cm^2^ vs RIV15cm^2^ | 1 | 1.173 (0.812, 1.696) | - | - | - |
| RIV10cm^2^ vs RIV10cm^2^+MEM20mg | 1 | 1.400 (0.596, 3.287) | - | - | - |
| RIV5cm^2^ vs PLA | 1 | **1.530 (1.005, 2.331)** | - | - | - |
| GAL24mg vs PLA | 7 | **1.386 (1.057, 1.818)** | 0.016 | 61.5% | 0.0723 |
| GAL24mg vs DON10mg | 2 | 1.089 (0.571, 2.080) | 0.373 | 0.0% | 0.0000 |
| GAL24mg vs GAL32mg | 2 | 0.687 (0.510, 0.925) | 0.659 | 0.0% | 0.0000 |
| GAL32mg vs PLA | 2 | **2.628 (1.876, 3.682)** | 0.304 | 5.2% | 0.0031 |
| MEM20mg vs PLA | 2 | 1.288 (0.795, 2.084) | 0.231 | 30.2% | 0.0372 |
| HupA400μg vs PLA | 1 | 1.844 (0.740, 4.591) | - | - | - |
| HupA400μg vs HupA200μg | 1 | 1.530 (0.627, 3.730) | - | - | - |
| HupA200μg vs PLA | 1 | 1.205 (0.458, 3.172) | - | - | - |
| EGb160mg vs PLA | 1 | 0.679 (0.183, 2.510) | - | - | - |
| EGb160mg vs DON5mg | 1 | 1.313 (0.308, 5.598) | - | - | - |
| EGb240mg vs PLA | 1 | 0.742 (0.436, 1.262) | - | - | - |
| EGb240mg vs EGb240mg+DON10mg | 1 | 0.322 (0.032, 3.279) | - | - | - |
| **Safety for mild to moderate AD** | | | | | |
| **Direct comparisons** | **No. of studies** | **OR (95% CI)** | **P-value** | ***I*²** | **τ²** |
| DON10mg vs PLA | 10 | **0.749 (0.624, 0.900)** | 0.528 | 0.0% | 0.0000 |
| DON10mg vs EGb240mg+DON10mg | 1 | 0.482 (0.171, 1.360) | - | - | - |
| DON10mg vs EGb240mg | 1 | **0.179 (0.061, 0.523)** | - | - | - |
| DON5mg vs PLA | 3 | 0.791 (0.529, 1.183) | 0.108 | 55.2% | 0.0696 |
| DON5mg vs DON10mg | 2 | **1.694 (1.210, 2.372)** | 0.856 | 0.0% | 0.0000 |
| RIV12mg vs PLA | 3 | **0.345 (0.217, 0.551)** | 0.063 | 63.8% | 0.1077 |
| RIV12mg vs RIV10cm^2^ | 2 | **0.675 (0.514, 0.887)** | 0.262 | 20.4% | 0.0079 |
| RIV10cm^2^ vs PLA | 2 | 0.693 (0.458, 1.049) | 0.124 | 57.7% | 0.0524 |
| RIV10cm^2^ vs RIV5cm^2^ | 1 | 0.980 (0.608, 1.580) | - | - | - |
| RIV10cm^2^ vs RIV15cm^2^ | 1 | 1.399 (0.968, 2.022) | - | - | - |
| RIV10cm^2^ vs RIV10cm^2^+MEM20mg | 1 | 1.119 (0.614, 2.040) | - | - | - |
| RIV5cm^2^ vs PLA | 1 | **0.557 (0.359, 0.862)** | - | - | - |
| GAL24mg vs PLA | 7 | **0.630 (0.488, 0.815)** | 0.018 | 60.8% | 0.0637 |
| GAL24mg vs DON10mg | 2 | 0.976 (0.633, 1.503) | 0.433 | 0.0% | 0.0000 |
| GAL24mg vs GAL32mg | 2 | 1.418 (0.914, 2.200) | 0.313 | 1.8% | 0.0020 |
| GAL32mg vs PLA | 2 | **0.361 (0.242, 0.537)** | 0.484 | 0.0% | 0.0000 |
| MEM20mg vs PLA | 2 | 0.982 (0.735, 1.313) | 0.372 | 0.0% | 0.0000 |
| EGb240mg vs PLA | 2 | 1.305 (0.944, 1.804) | 0.924 | 0.0% | 0.0000 |
| EGb240mg vs EGb240mg+DON10mg | 1 | 2.700 (0.967, 7.541) | - | - | - |
| **Secondary outcomes** | | | | | |
| **Efficacy on clinical global impression (CIBIC+)** **mild to moderate AD** | | | | | |
| **Direct comparisons** | **No. of studies** | **SMD (95% CI)** | **P-value** | ***I*²** | **τ²** |
| DON10mg vs PLA | 5 | **-0.405 (-0.534, -0.275)** | 0.555 | 0.0% | 0.0000 |
| DON10mg vs MEM20mg | 1 | 0.188 (-0.116, 0.492) | - | - | - |
| DON5mg vs PLA | 2 | **-0.334 (-0.494, -0.173)** | 0.899 | 0.0% | 0.0000 |
| DON5mg vs DON10mg | 2 | 0.091 (-0.068, 0.251) | 0.904 | 0.0% | 0.0000 |
| RIV12mg vs PLA | 3 | **-0.399 (-0.544, -0.254)** | 0.336 | 8.4% | 0.0016 |
| RIV10cm^2^ vs PLA | 1 | **-0.211 (-0.380, -0.041)** | - | - | - |
| RIV10cm^2^ vs RIV5cm^2^ | 1 | 0.000 (-0.169, 0.169) | - | - | - |
| RIV5cm^2^ vs PLA | 1 | **-0.211 (-0.380, -0.041)** | - | - | - |
| MEM20mg vs PLA | 2 | -0.191 (-0.440, 0.057) | 0.084 | 66.6% | 0.0214 |
| **Efficacy on function (ADCS-ADL)** **mild to moderate AD** | | | | | |
| **Direct comparisons** | **No. of studies** | **SMD (95% CI)** | **P-value** | ***I*²** | **τ²** |
| DON10mg vs PLA | 2 | 0.230 (-0.022, 0.483) | 0.765 | 0.0% | 0.0000 |
| DON10mg vs EGb240mg+DON10mg | 1 | 0.129 (-0.365, 0.624) | - | - | - |
| DON10mg vs EGb240mg | 1 | 0.285 (-0.215, 0.786) | - | - | - |
| DON10mg vs MEM20mg | 1 | -0.014 (-0.319, 0.292) | - | - | - |
| RIV12mg vs PLA | 2 | 0.076 (-0.301, 0.453) | 0.179 | 44.6% | 0.0426 |
| RIV12mg vs RIV10cm^2^ | 2 | -0.031 (-0.157, 0.094) | 0.849 | 0.0% | 0.0000 |
| RIV10cm^2^ vs PLA | 1 | **0.238 (0.066, 0.409)** | - | - | - |
| RIV10cm^2^ vs RIV15cm^2^ | 1 | **-0.305 (-0.475, -0.134)** | - | - | - |
| RIV10cm^2^ vs RIV10cm^2^+MEM20mg | 1 | -0.122 (-0.435, 0.191) | - | - | - |
| GAL24mg vs PLA | 2 | **0.250 (0.127, 0.374)** | 0.853 | 0.0% | 0.0000 |
| GAL24mg vs DON10mg | 1 | -0.099 (-0.356, 0.158) | - | - | - |
| MEM20mg vs PLA | 2 | 0.010 (-0.133, 0.153) | 0.987 | 0.0% | 0.0000 |
| HupA400μg vs PLA | 1 | 0.216 (-0.115, 0.548) | - | - | - |
| HupA400μg vs HupA200μg | 1 | 0.039 (-0.296, 0.374) | - | - | - |
| HupA200μg vs PLA | 1 | 0.164 (-0.165, 0.494) | - | - | - |
| EGb240mg vs EGb240mg+DON10mg | 1 | -0.098 (-0.600, 0.405) | - | - | - |
| **Efficacy on behavior (NPI)** **mild to moderate AD** | | | | | |
| **Direct comparisons** | **No. of studies** | **SMD (95% CI)** | **P-value** | ***I*²** | **τ²** |
| DON10mg vs PLA | 3 | -0.071 (-0.357, 0.215) | 0.107 | 55.2% | 0.0353 |
| DON10mg vs EGb240mg+DON10mg | 1 | 0.072 (-0.422, 0.566) | - | - | - |
| DON10mg vs EGb240mg | 1 | -0.158 (-0.657, 0.341) | - | - | - |
| DON10mg vs MEM20mg | 1 | 0.206 (-0.099, 0.511) | - | - | - |
| RIV12mg vs PLA | 1 | -0.039 (-0.209, 0.131) | - | - | - |
| RIV12mg vs RIV10cm^2^ | 2 | -0.032 (-0.158, 0.093) | 0.870 | 0.0% | 0.0000 |
| RIV10cm^2^ vs PLA | 1 | 0.000 (-0.171, 0.171) | - | - | - |
| GAL24mg vs PLA | 2 | **-0.154 (-0.277, -0.030)** | 0.839 | 0.0% | 0.0000 |
| GAL24mg vs DON10mg | 1 | 0.012 (-0.245, 0.269) | - | - | - |
| MEM20mg vs PLA | 2 | -0.031 (-0.383, 0.320) | 0.015 | 83.1% | 0.0535 |
| HupA400μg vs PLA | 1 | 0.039 (-0.291, 0.370) | - | - | - |
| HupA400μg vs HupA200μg | 1 | 0.050 (-0.285, 0.385) | - | - | - |
| HupA200μg vs PLA | 1 | -0.019 (-0.348, 0.310) | - | - | - |
| EGb240mg vs PLA | 1 | **-0.508 (-0.727, -0.290)** | - | - | - |
| EGb240mg vs EGb240mg+DON10mg | 1 | 0.220 (-0.284, 0.723) | - | - | - |

Abbreviation: DON= donepezil, EGb= EGb761, GAL= galantamine, HupA= huperzine A, MEM= memantine, PLA= placebo, RIV= rivastigmine.

Supplementary 4

Assessment of the inconsistency results for each outcome

Efficacy on cognitive (ADAS-cog) for mild to moderate AD

| Loop | IF | seIF | z_value | p_value | 95%CI | τ² |
| --- | --- | --- | --- | --- | --- | --- |
| PLA-RIV10cm^2^-RIV12mg | 0.352 | 0.634 | 0.554 | 0.579 | (0.00,1.59) | 0.110 |
| DON10mg-DON5mg-PLA | 0.231 | 0.158 | 1.461 | 0.144 | (0.00,0.54) | 0.011 |
| GAL24mg-GAL32mg-PLA | 0.173 | 0.255 | 0.677 | 0.499 | (0.00,0.67) | 0.044 |
| HupA200ug-HupA400ug-PLA | 0.165 | 0.294 | 0.562 | 0.574 | (0.00,0.74) | 0.000 |
| DON10mg-MEM20mg-PLA | 0.122 | 0.252 | 0.483 | 0.629 | (0.00,0.62) | 0.019 |
| PLA-RIV10cm^2^-RIV5cm^2^ | 0.000 | 0.136 | 0.001 | 0.999 | (0.00,0.27) | 0.000 |


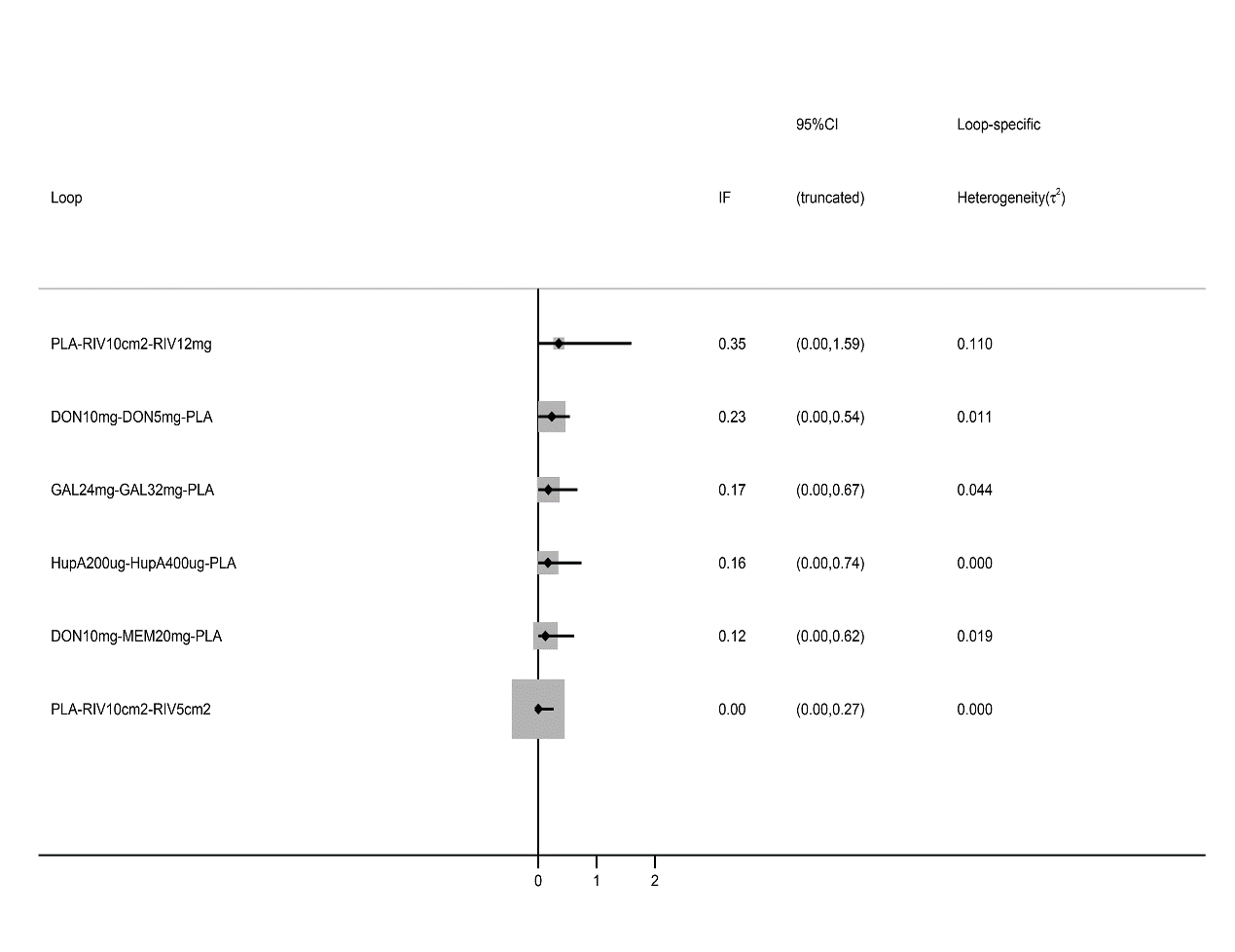


Efficacy on cognitive (MMSE) for mild to moderate AD

| Loop | IF | seIF | z_value | p_value | 95%CI | τ² |
| --- | --- | --- | --- | --- | --- | --- |
| PLA-RIV10cm^2^-RIV12mg | 0.137 | 0.768 | 0.179 | 0.858 | (0.00,1.64) | 0.089 |
| PLA-RIV10cm^2^-RIV5cm^2^ | 0.116 | 0.143 | 0.808 | 0.419 | (0.00,0.40) | 0.000 |
| DON10mg-DON5mg-PLA | 0.076 | 0.125 | 0.611 | 0.541 | (0.00,0.32) | 0.000 |
| DON5mg-EGb160mg-PLA | 0.073 | 0.406 | 0.180 | 0.857 | (0.00,0.87) | 0.000 |
| HupA200ug-HupA400ug-PLA | 0.012 | 0.295 | 0.042 | 0.967 | (0.00,0.59) | 0.000 |


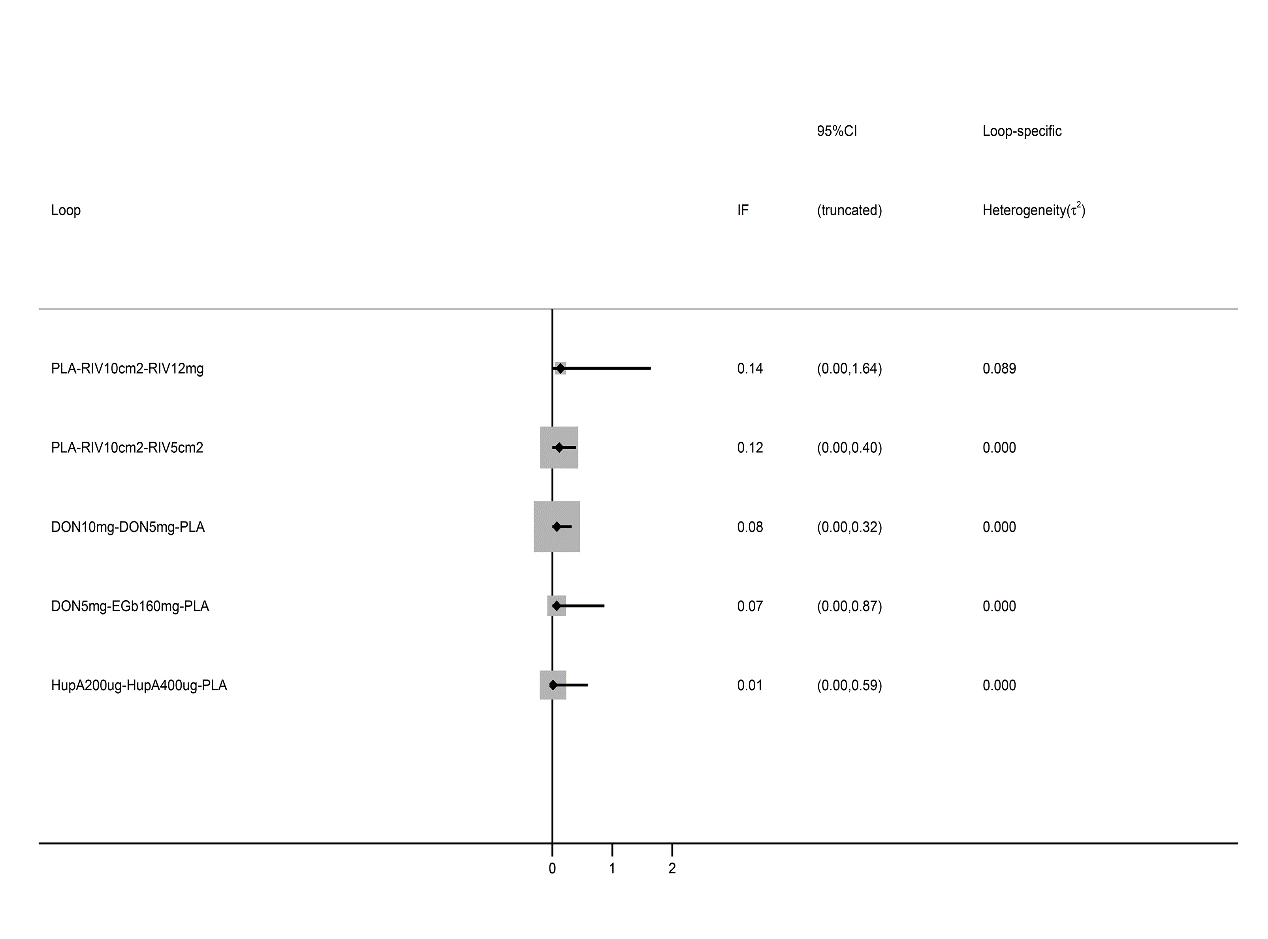


Acceptability for mild to moderate AD

| Loop | IF | seIF | z_value | p_value | 95%CI | τ² |
| --- | --- | --- | --- | --- | --- | --- |
| DON10mg-EGb240mg-PLA | 0.941 | 1.420 | 0.663 | 0.507 | (0.00,3.72) | 0.162 |
| DON5mg-EGb160mg-PLA | 0.650 | 1.027 | 0.633 | 0.527 | (0.00,2.66) | 0.014 |
| DON10mg-DON5mg-PLA | 0.375 | 0.410 | 0.915 | 0.360 | (0.00,1.18) | 0.125 |
| GAL24mg-GAL32mg-PLA | 0.271 | 0.345 | 0.785 | 0.432 | (0.00,0.95) | 0.052 |
| PLA-RIV10cm^2^-RIV5cm^2^ | 0.199 | 0.335 | 0.595 | 0.552 | (0.00,0.86) | 0.000 |
| PLA-RIV10cm^2^-RIV12mg | 0.097 | 0.448 | 0.216 | 0.829 | (0.00,0.97) | 0.090 |
| DON10mg-GAL24mg-PLA | 0.012 | 0.477 | 0.024 | 0.981 | (0.00,0.95) | 0.107 |
| DON10mg-EGb240mg-EGb240mg+DON10mg | 0.000 | 1.836 | 0.000 | 1.000 | (0.00,3.60) | 0.000 |
| HupA200ug-HupA400ug-PLA | 0.000 | 0.817 | 0.000 | 1.000 | (0.00,1.60) | 0.000 |

**
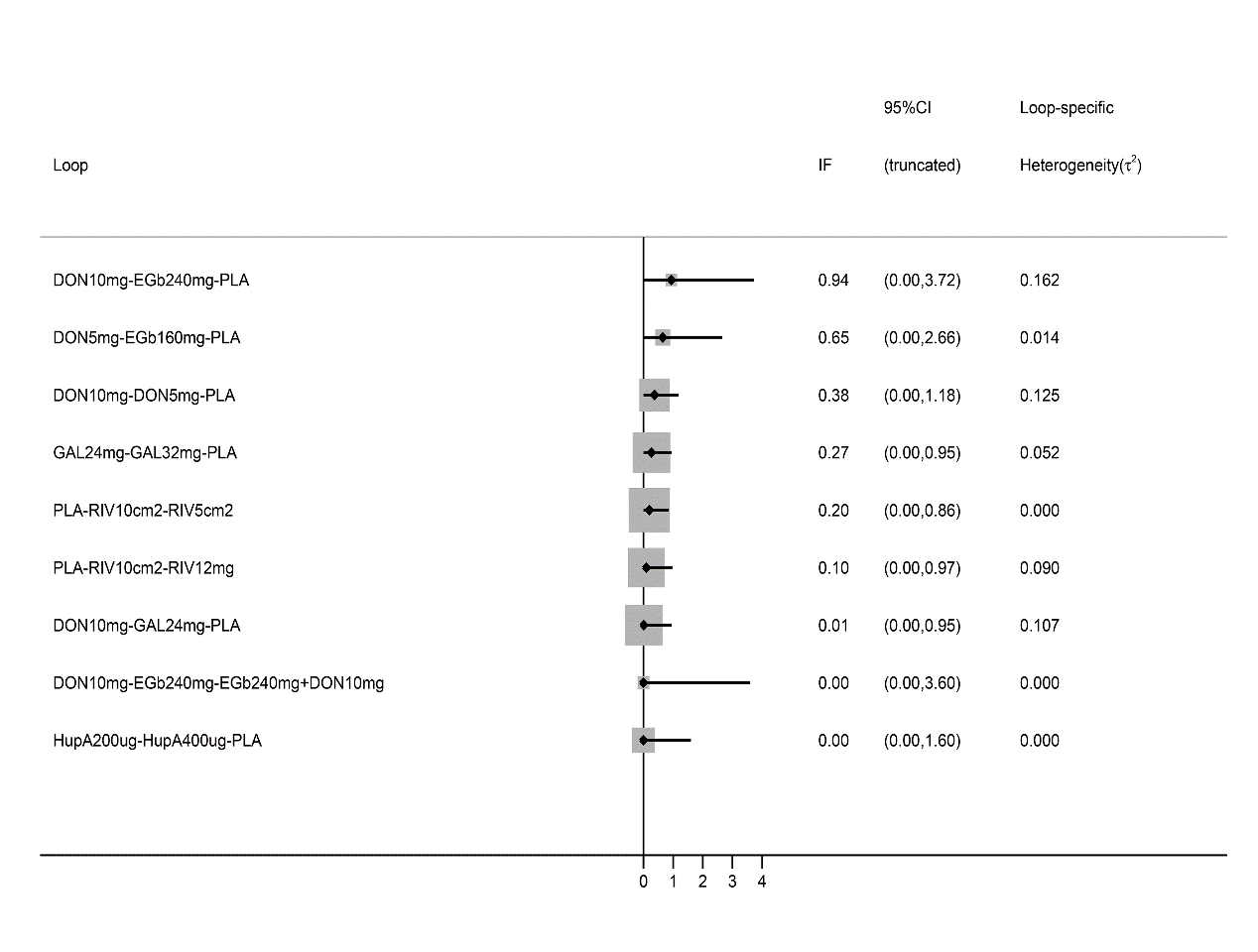
**

**Safety for mild to moderate AD**

| Loop | IF | seIF | z_value | p_value | 95%CI | τ² |
| --- | --- | --- | --- | --- | --- | --- |
| DON10mg-EGb240mg-PLA | 1.168 | 0.580 | 2.014 | 0.044 | (0.03,2.31) | 0.000 |
| DON10mg-DON5mg-PLA | 0.459 | 0.245 | 1.872 | 0.061 | (0.00,0.94) | 0.004 |
| PLA-RIV10cm^2^-RIV12mg | 0.282 | 0.359 | 0.785 | 0.432 | (0.00,0.99) | 0.056 |
| PLA-RIV10cm^2^-RIV5cm^2^ | 0.276 | 0.356 | 0.775 | 0.438 | (0.00,0.97) | 0.000 |
| GAL24mg-GAL32mg-PLA | 0.246 | 0.425 | 0.580 | 0.562 | (0.00,1.08) | 0.053 |
| DON10mg-GAL24mg-PLA | 0.100 | 0.300 | 0.332 | 0.740 | (0.00,0.69) | 0.031 |
| DON10mg-EGb240mg-EGb240mg+DON10mg | 0.000 | 0.925 | 0.000 | 1.000 | (0.00,1.81) | 0.000 |

**
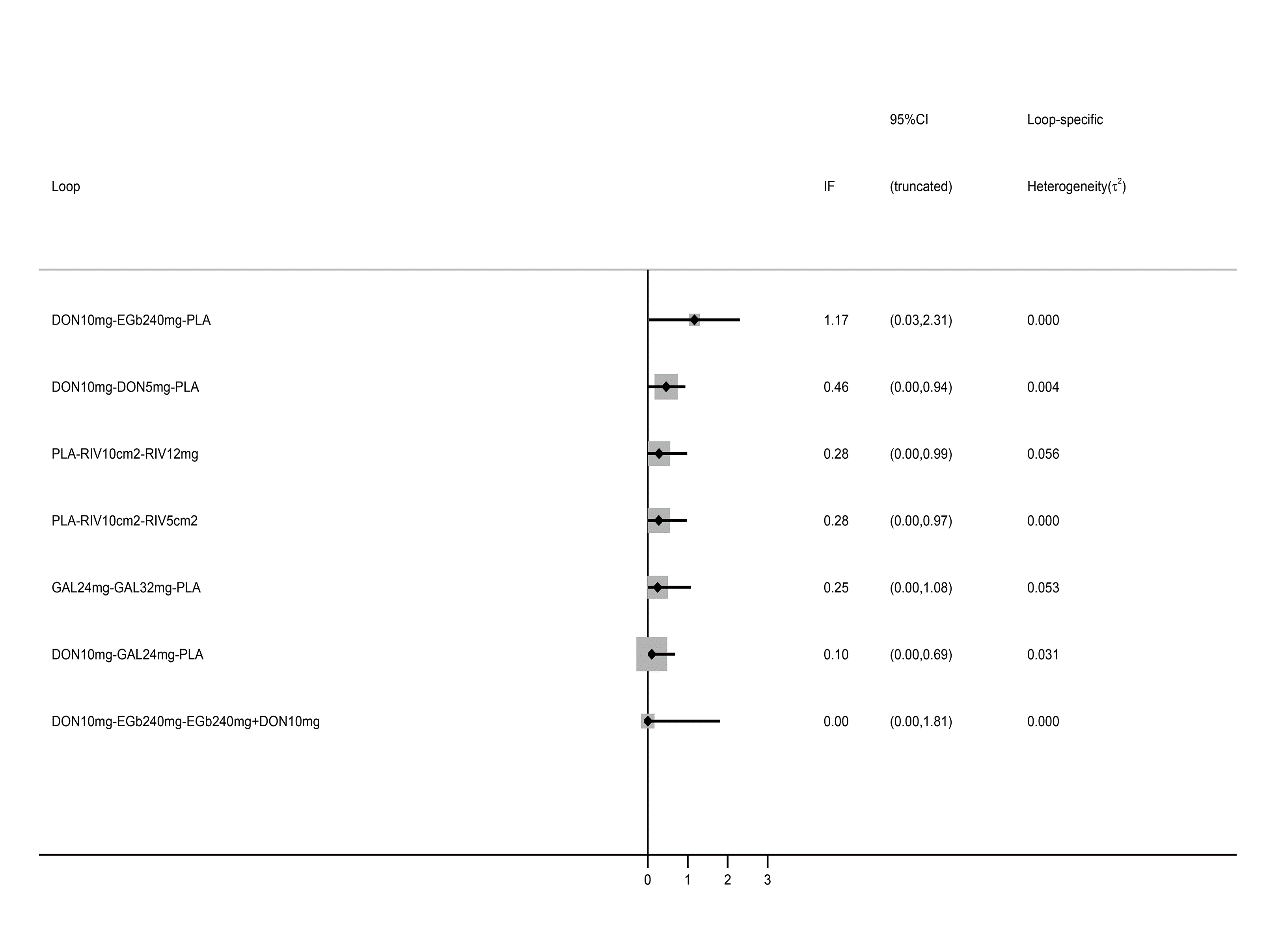
**

Efficacy on clinical global impression (CIBIC+) for mild to moderate AD

| Loop | IF | seIF | z_value | p_value | 95%CI | τ² |
| --- | --- | --- | --- | --- | --- | --- |
| DON10mg-MEM20mg-PLA | 0.276 | 0.197 | 1.402 | 0.161 | (0.00,0.66) | 0.000 |
| DON10mg-DON5mg-PLA | 0.020 | 0.133 | 0.153 | 0.878 | (0.00,0.28) | 0.000 |
| PLA-RIV10cm^2^-RIV5cm^2^ | 0.000 | 0.150 | 0.000 | 1.000 | (0.00,0.29) | 0.000 |

**
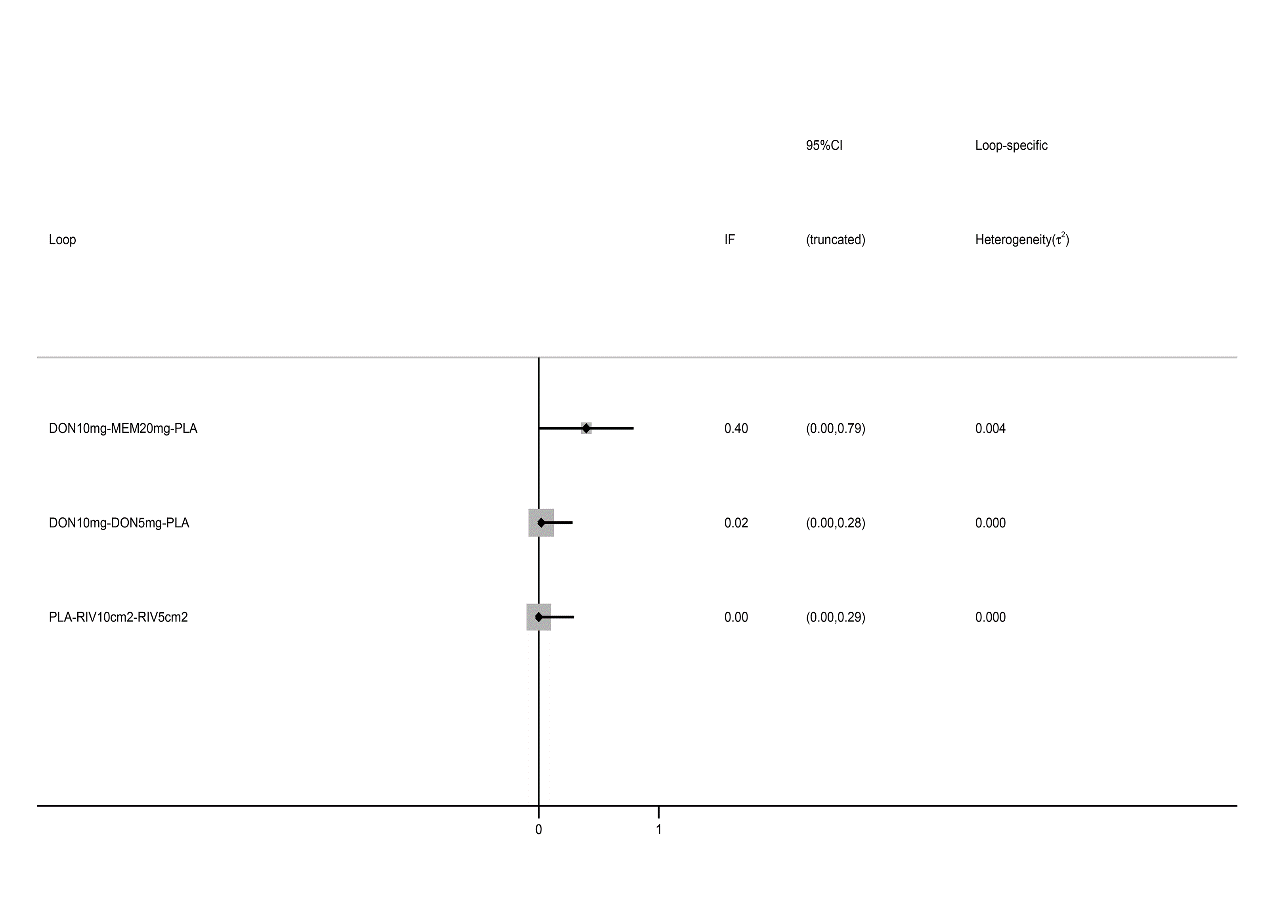
**

Efficacy on function (ADCS-ADL) for mild to moderate AD

| Loop | IF | seIF | z_value | p_value | 95%CI | τ² |
| --- | --- | --- | --- | --- | --- | --- |
| DON10mg-MEM20mg-PLA | 0.234 | 0.215 | 1.087 | 0.277 | (0.00,0.65) | 0.000 |
| DON10mg-GAL24mg-PLA | 0.119 | 0.194 | 0.612 | 0.540 | (0.00,0.50) | 0.000 |
| DON10mg-EGb240mg-EGb240mg+DON10mg | 0.058 | 0.441 | 0.132 | 0.895 | (0.00,0.92) | 0.000 |
| PLA-RIV10cm^2^-RIV12mg | 0.047 | 0.137 | 0.343 | 0.732 | (0.00,0.32) | 0.000 |
| HupA200ug-HupA400ug-PLA | 0.013 | 0.293 | 0.044 | 0.965 | (0.00,0.59) | 0.000 |

**
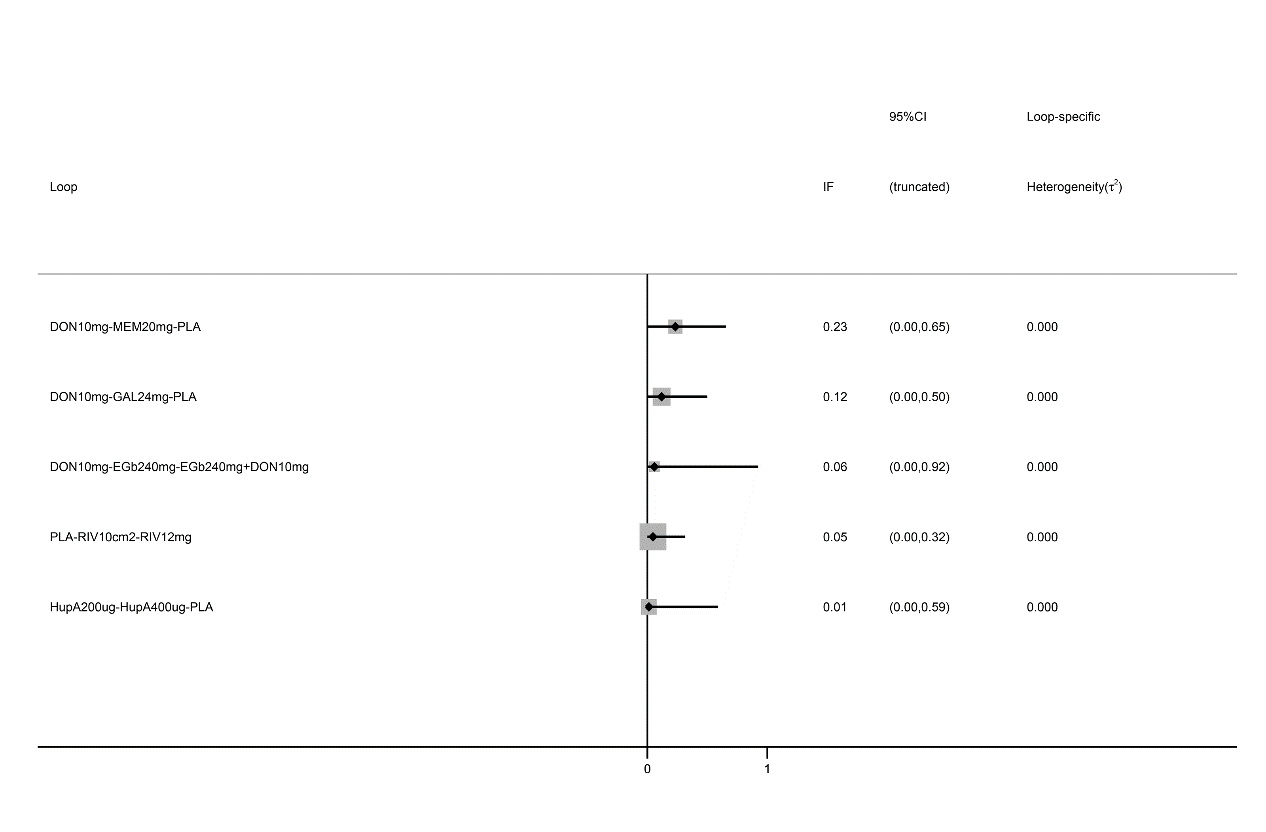
**

Efficacy on behavior (NPI) for mild to moderate AD

| Loop | IF | seIF | z_value | p_value | 95%CI | τ² |
| --- | --- | --- | --- | --- | --- | --- |
| DON10mg-EGb240mg-PLA | 0.595 | 0.411 | 1.447 | 0.148 | (0.00,1.40) | 0.035 |
| DON10mg-MEM20mg-PLA | 0.109 | 0.250 | 0.434 | 0.664 | (0.00,0.60) | 0.010 |
| DON10mg-GAL24mg-PLA | 0.081 | 0.226 | 0.359 | 0.719 | (0.00,0.52) | 0.008 |
| DON10mg-EGb240mg-EGb240mg+DON10mg | 0.010 | 0.441 | 0.022 | 0.982 | (0.00,0.87) | 0.000 |
| HupA200ug-HupA400ug-PLA | 0.008 | 0.293 | 0.027 | 0.978 | (0.00,0.58) | 0.000 |
| PLA-RIV10cm^2^-RIV12mg | 0.006 | 0.139 | 0.044 | 0.965 | (0.00,0.28) | 0.000 |

**
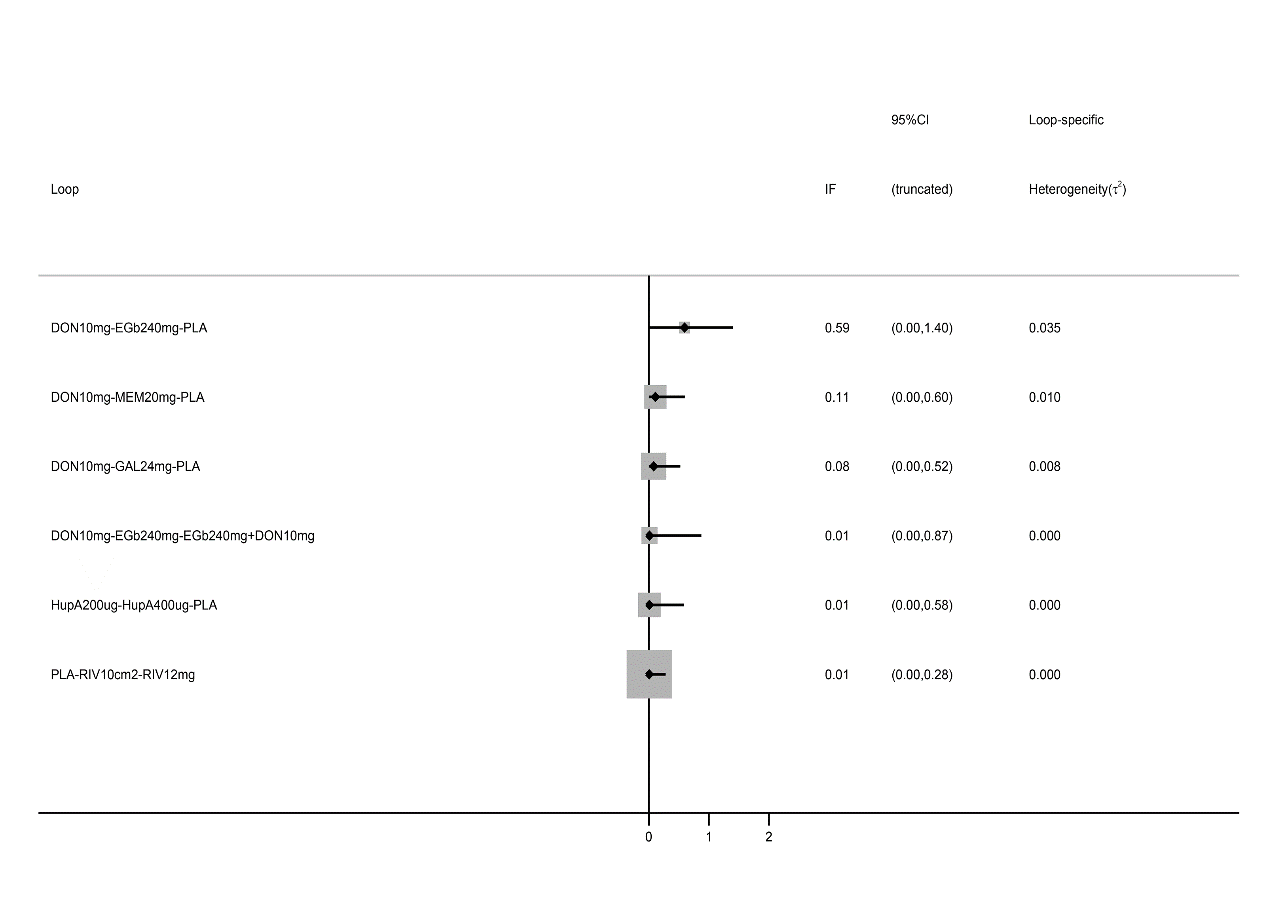
**

Supplementary 5

The surface under the cumulative ranking curve (SUCRA) of each outcome

Abbreviation: PLA=placebo, RIV=rivastigmine, GAL=galantamine, DON=donepezil, HupA=huperizine A, MEM=memantine, EGb=EGb761, NA=not available.

|  | ADAS-cog | MMSE | Acceptability | Safety | ADCS-ADL | CIBIC+ | NPI |
| --- | --- | --- | --- | --- | --- | --- | --- |
| PLA | 25.1 | 62.9 | 71.7 | 81.8 | 15.0 | 2.9 | 28.3 |
| RIV12mg | 22.5 | 72.9 | 21.1 | 8.8 | 46.1 | 83.7 | 40.9 |
| GAL24mg | 75.5 | 48.0 | 44.9 | 46.4 | 60.3 | NA | 59.7 |
| RIV10cm^2^ | 39.4 | 56.5 | 31.1 | 40.0 | 55.5 | 40.5 | 34.0 |
| DON10mg | 65.6 | 36.9 | 55.2 | 53.0 | 58.7 | 72.2 | 52.8 |
| HupA400μg | 48.0 | 29.5 | 29.9 | NA | 59.5 | NA | 30.2 |
| HupA200μg | 32.3 | 38.4 | 53.8 | NA | 50.9 | NA | 36.9 |
| RIV10cm^2^+MEM20mg | 51.6 | 63.1 | 55.3 | 34.0 | 71.1 | NA | NA |
| EGb240mg | 24.6 | NA | 87.5 | 97.8 | 25.1 | NA | 84.4 |
| EGb240mg+DON10mg | NA | NA | 54.9 | 66.9 | 38.0 | NA | 80.8 |
| GAL32mg | 93.2 | NA | 13.1 | 13.8 | NA | NA | NA |
| MEM20mg | 57.0 | 41.7 | 49.7 | 78.9 | 26.2 | 52.6 | 52.1 |
| RIV5cm^2^ | 39.5 | 60.7 | 31.5 | 38.3 | NA | 40.3 | NA |
| EGb160mg | NA | 49.7 | 72.5 | NA | NA | NA | NA |
| DON5mg | 73.3 | 39.7 | 83.4 | 71.7 | NA | 57.8 | NA |
| RIV15cm^2^ | 52.4 | NA | 44.4 | 18.9 | 93.7 | NA | NA |


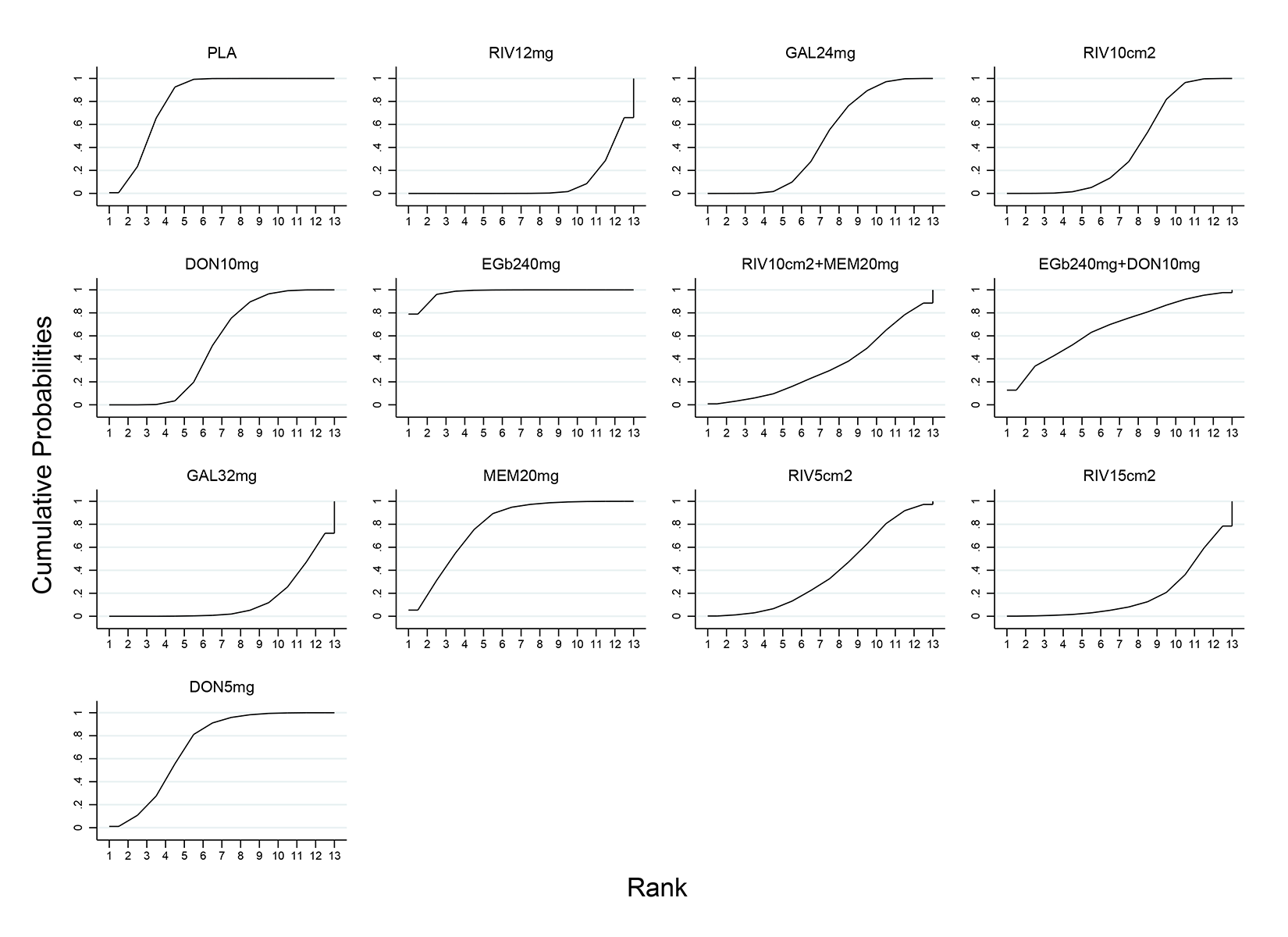


The SUCRA of safety


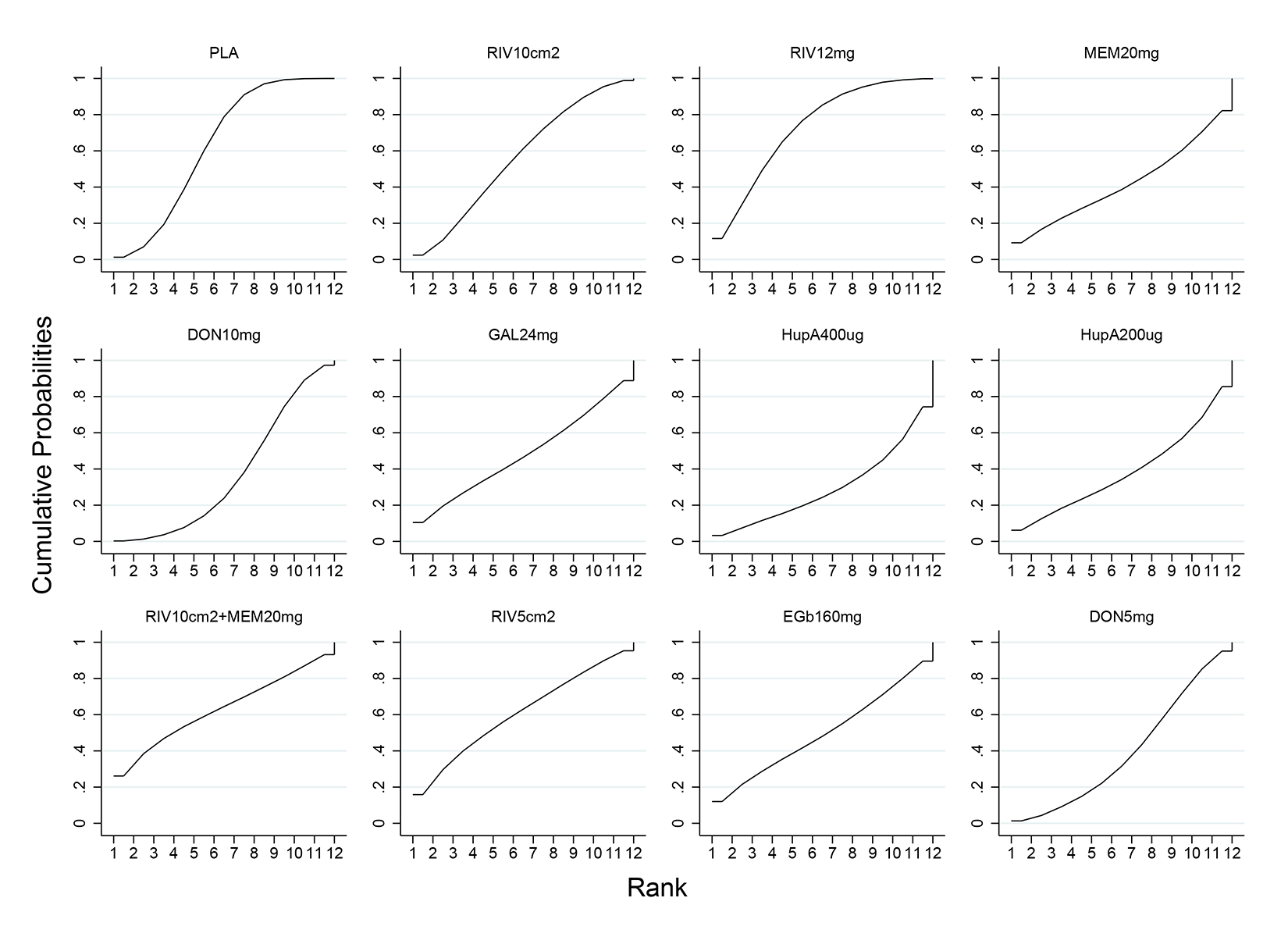


The SUCRA of efficacy on cognition (MMSE)


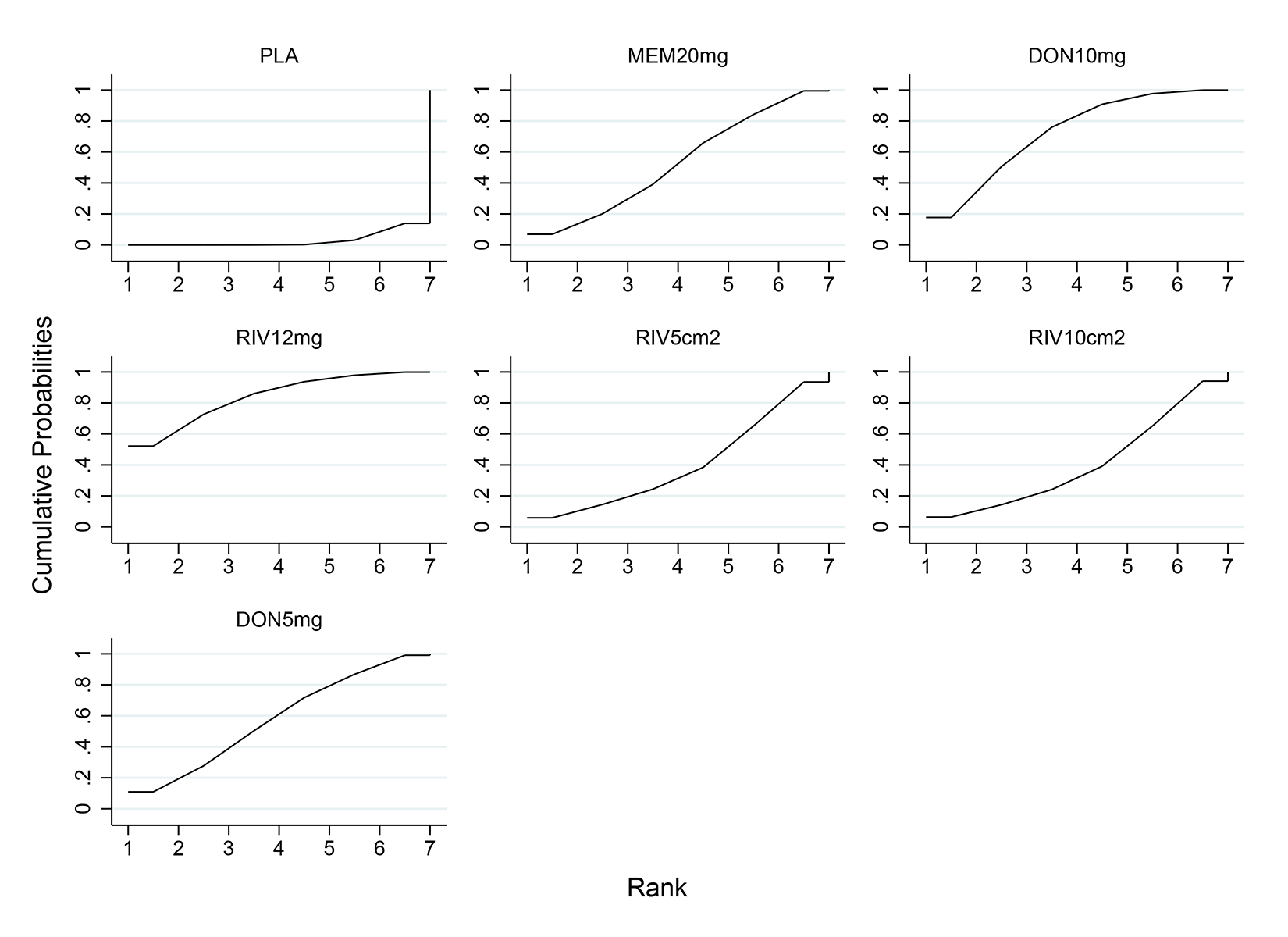


The SUCRA of efficacy on clinical global impression (CIBIC+)


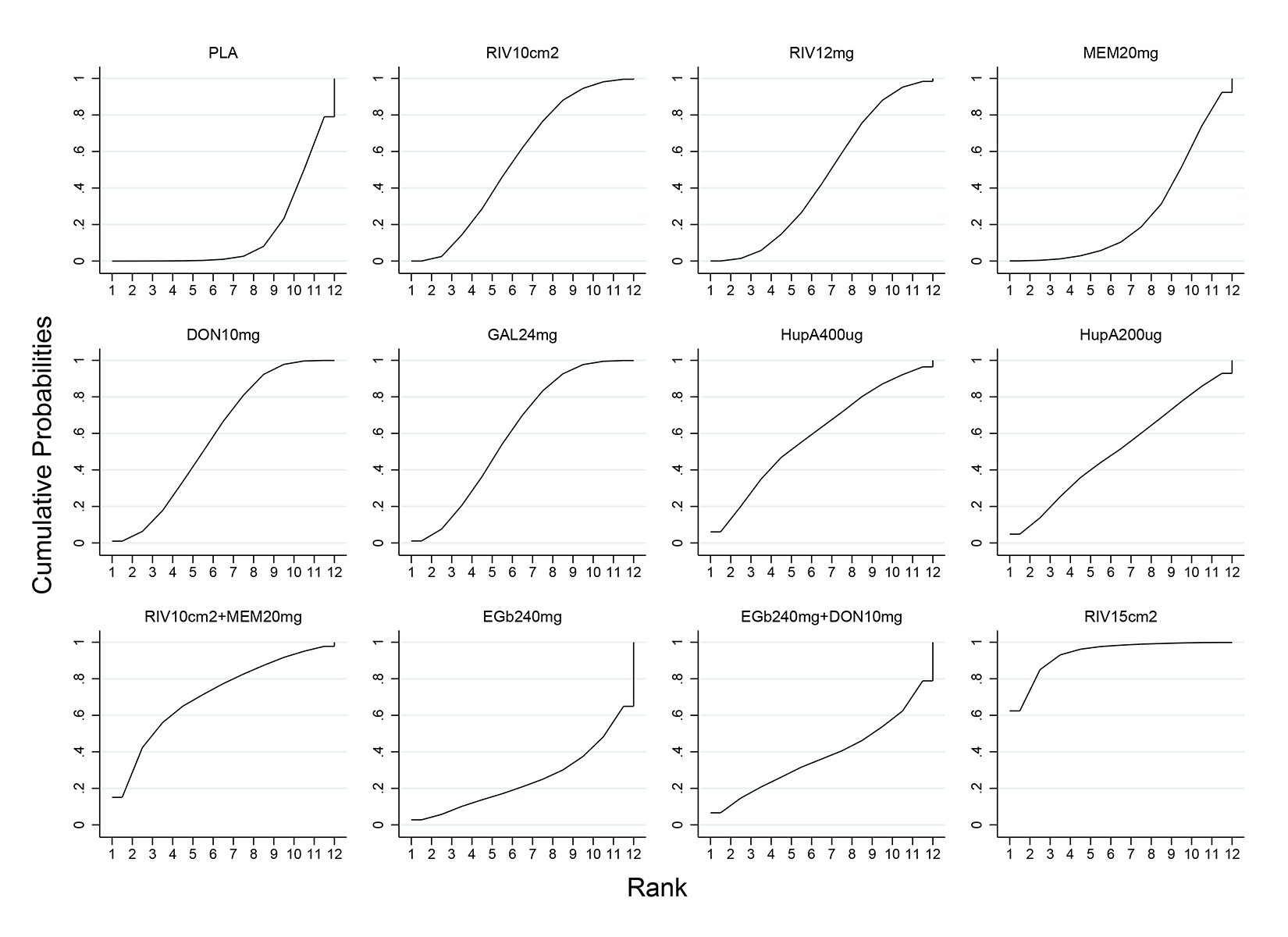


The SUCRA of efficacy on function (ADCS-ADL)


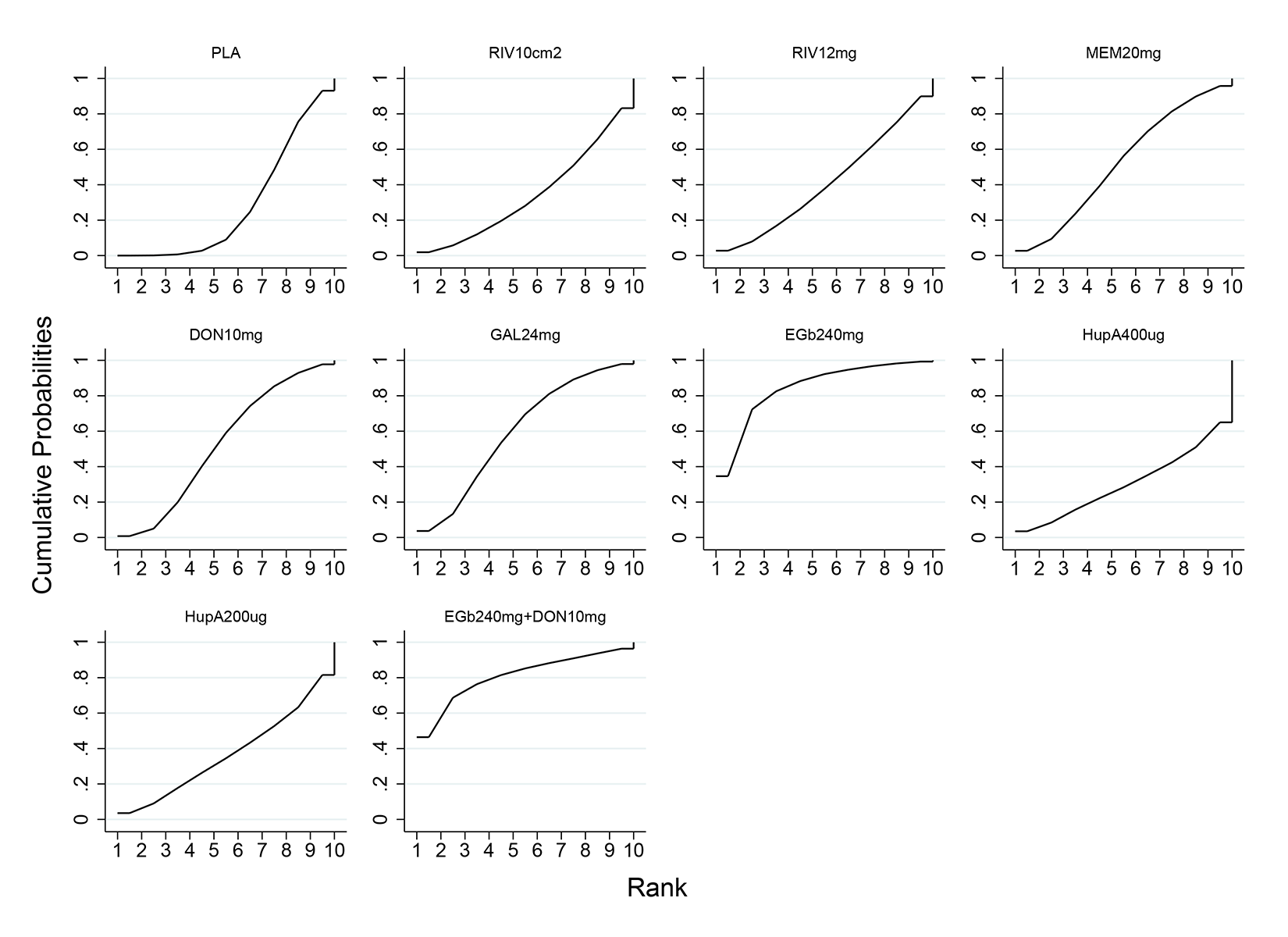


The SUCRA of efficacy on behavior (NPI)

Supplementary 6

Networkplot of secondary outcomes


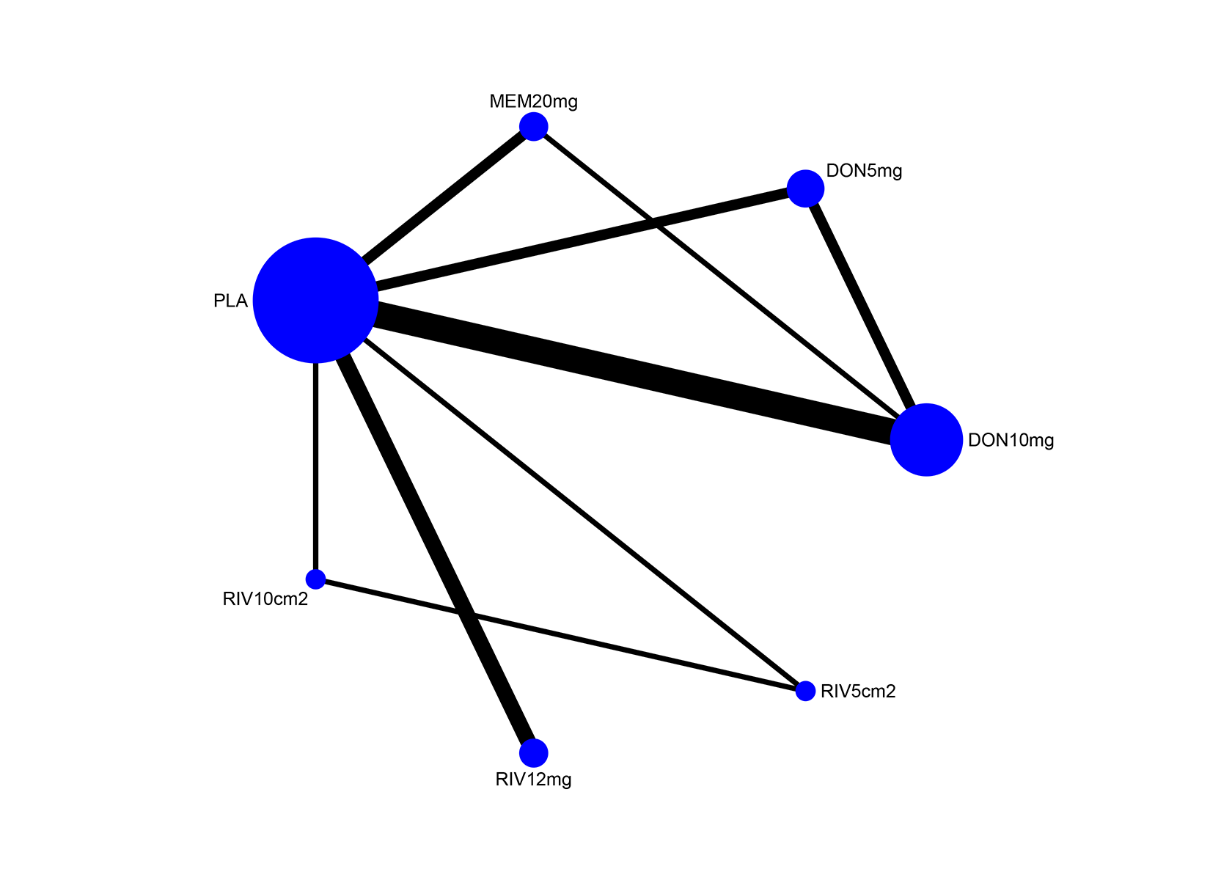


The networkplot of efficacy on clinical global impression (CIBIC+)


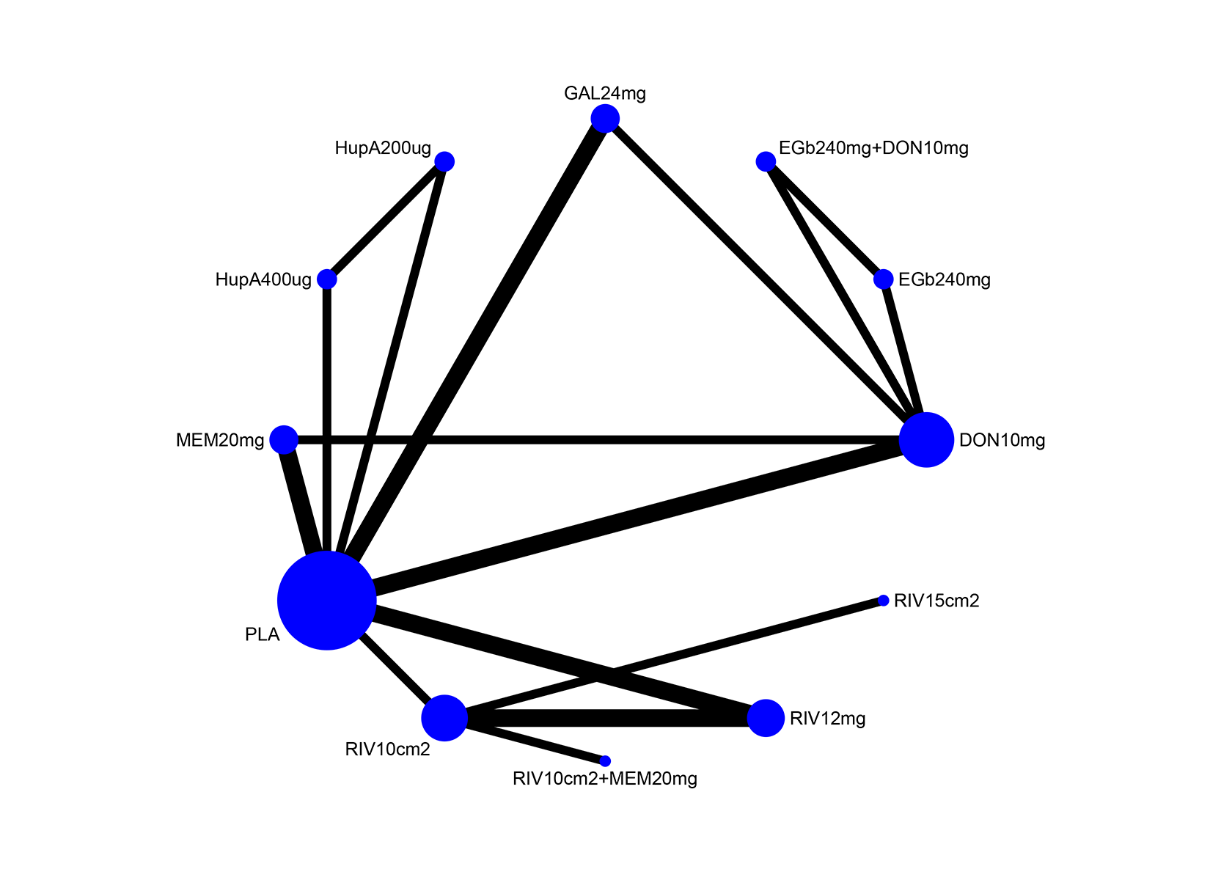


The networkplot of efficacy on function (ADCS-ADL)


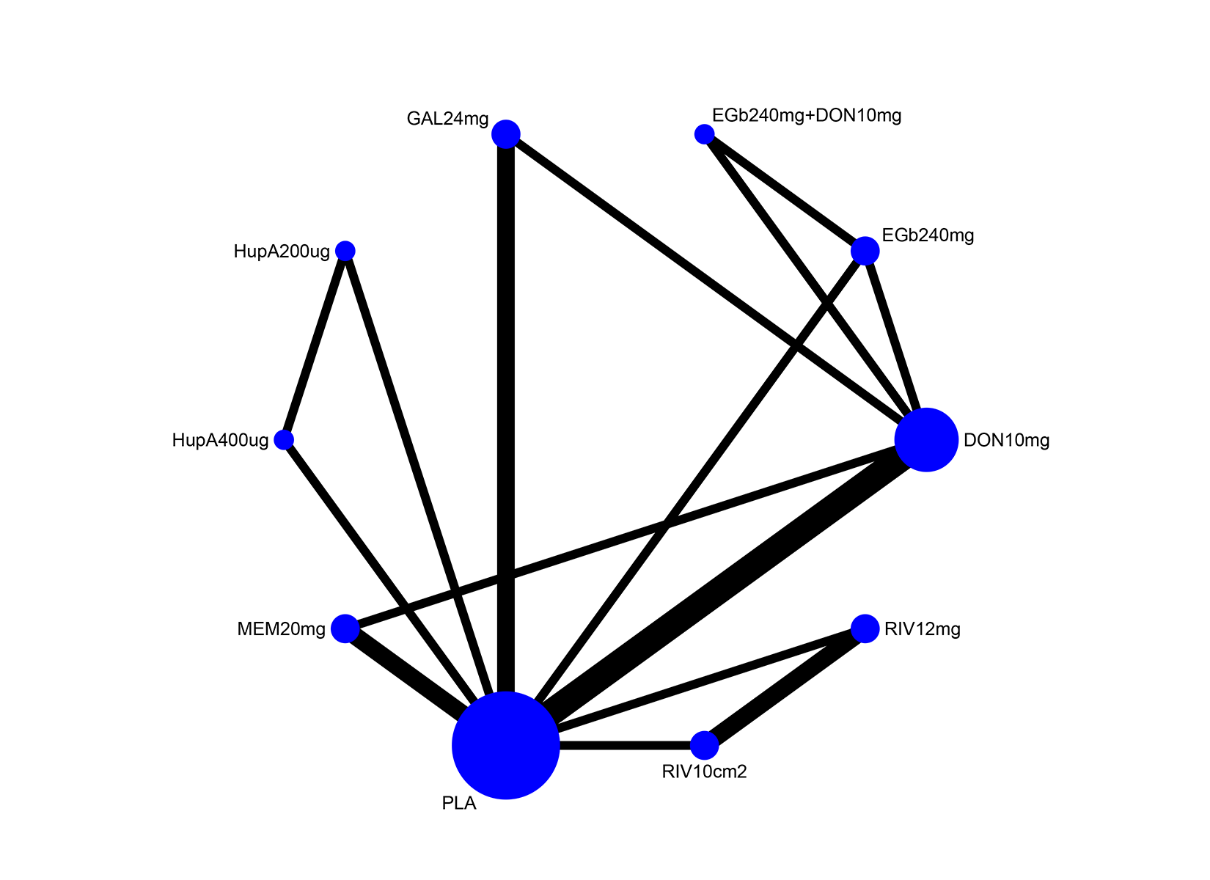


The networkplot of efficacy on behavior (NPI)

Supplementary 7

The Forest of secondary outcomes


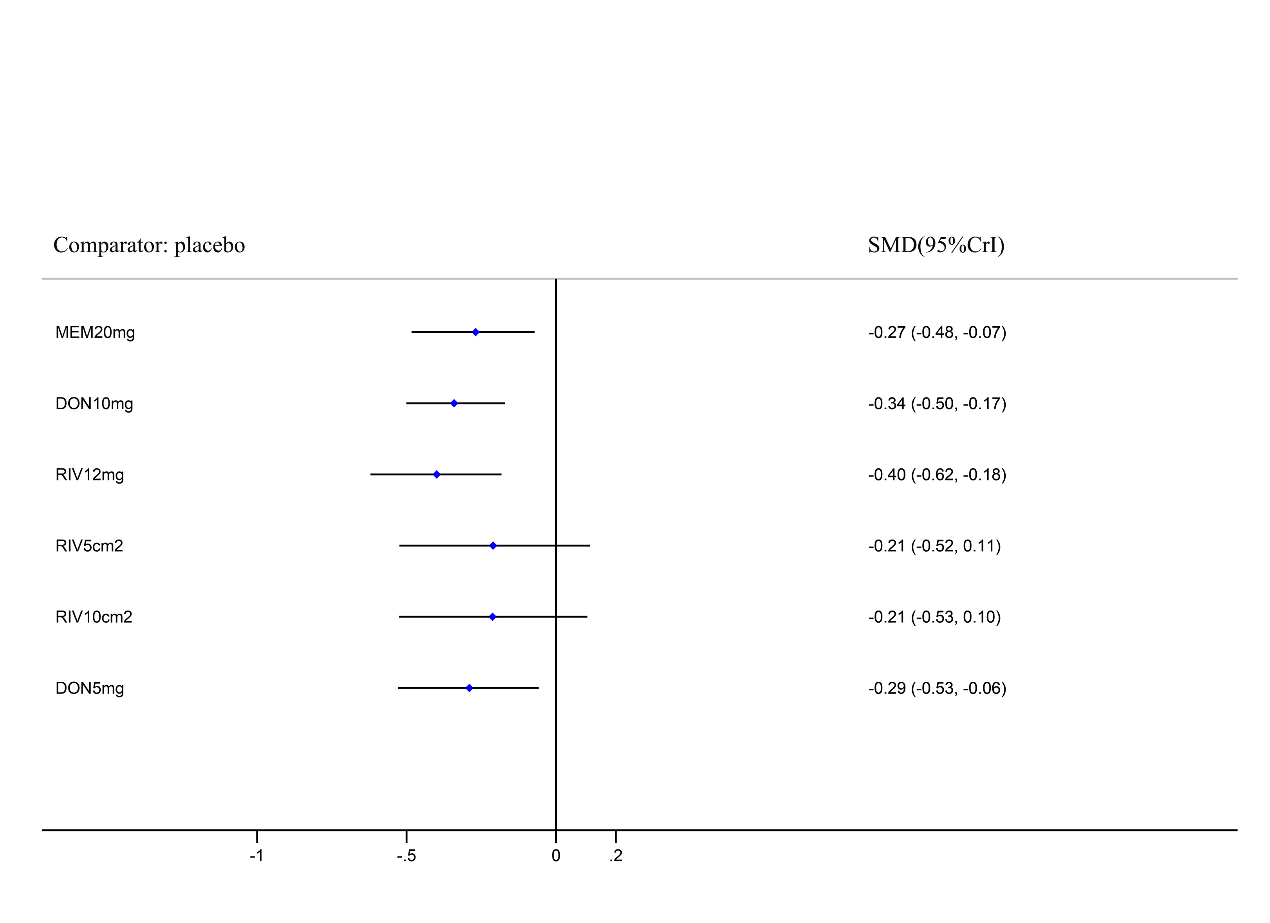


The Forest of efficacy on clinical global impression (CIBIC+)


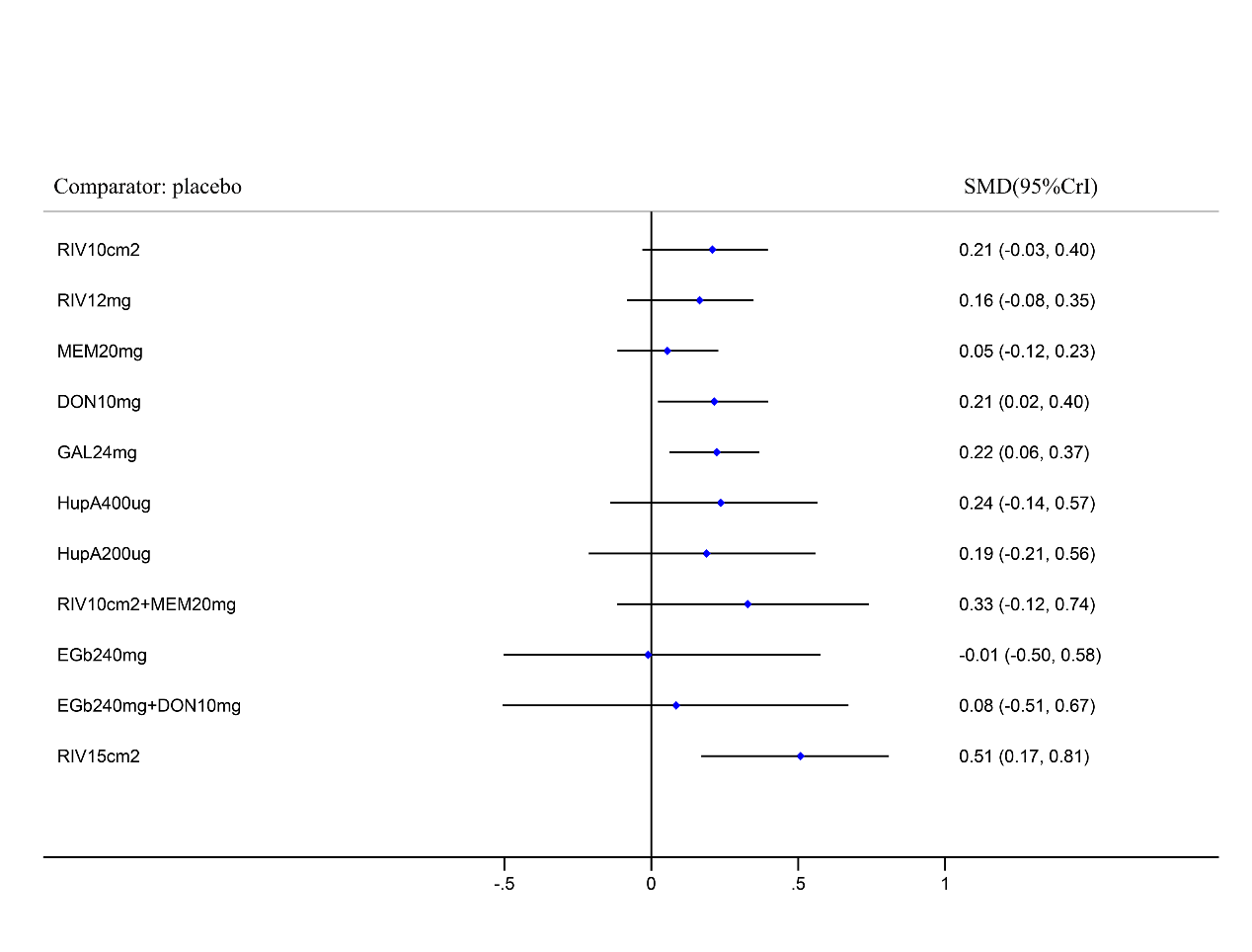


The Forest of efficacy on function (ADCS-ADL)


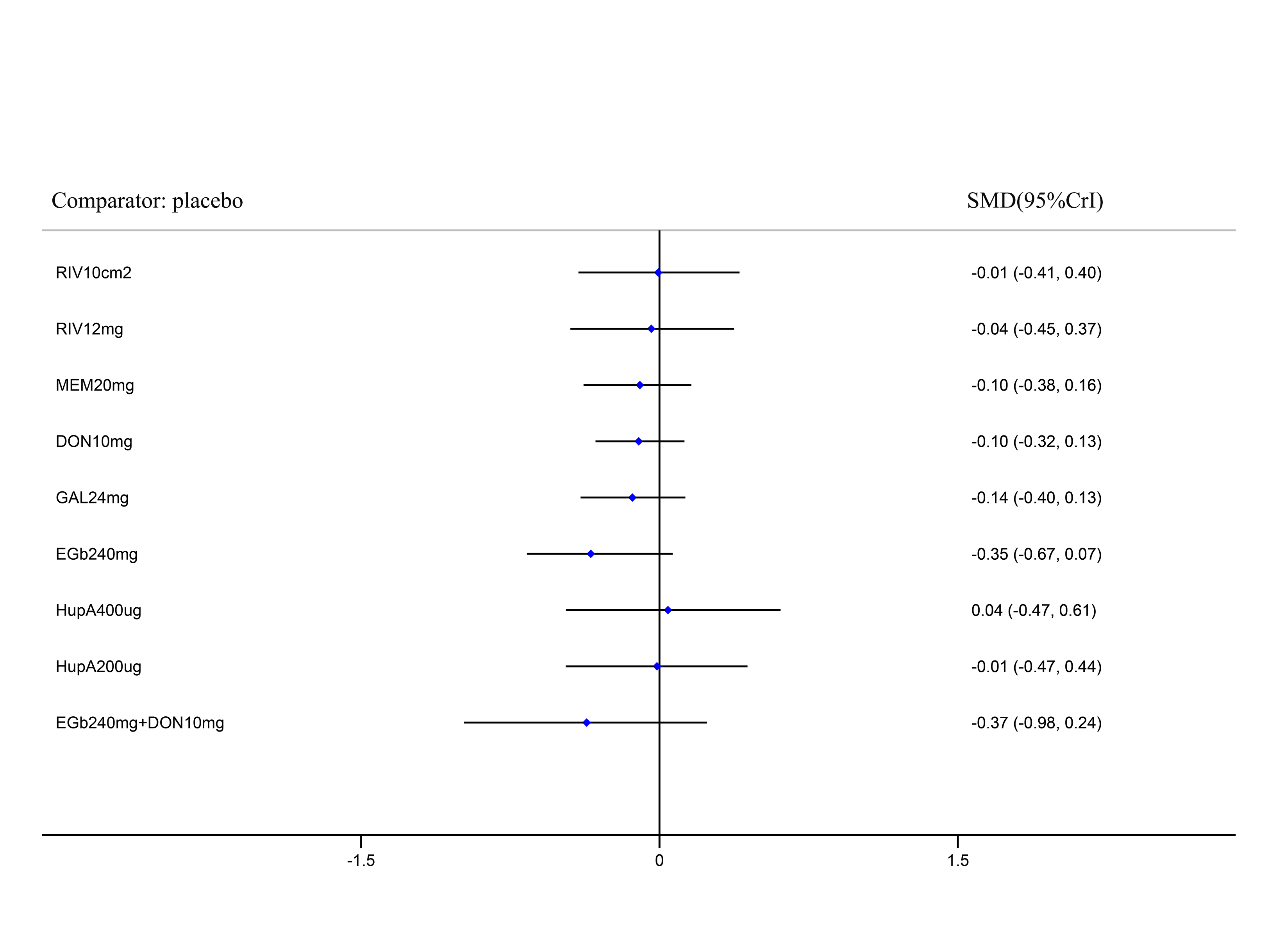


The Forest of efficacy on behavior (NPI)

Supplementary 8

comparison-adjusted funnel plot for each outcome from the network meta-analysis


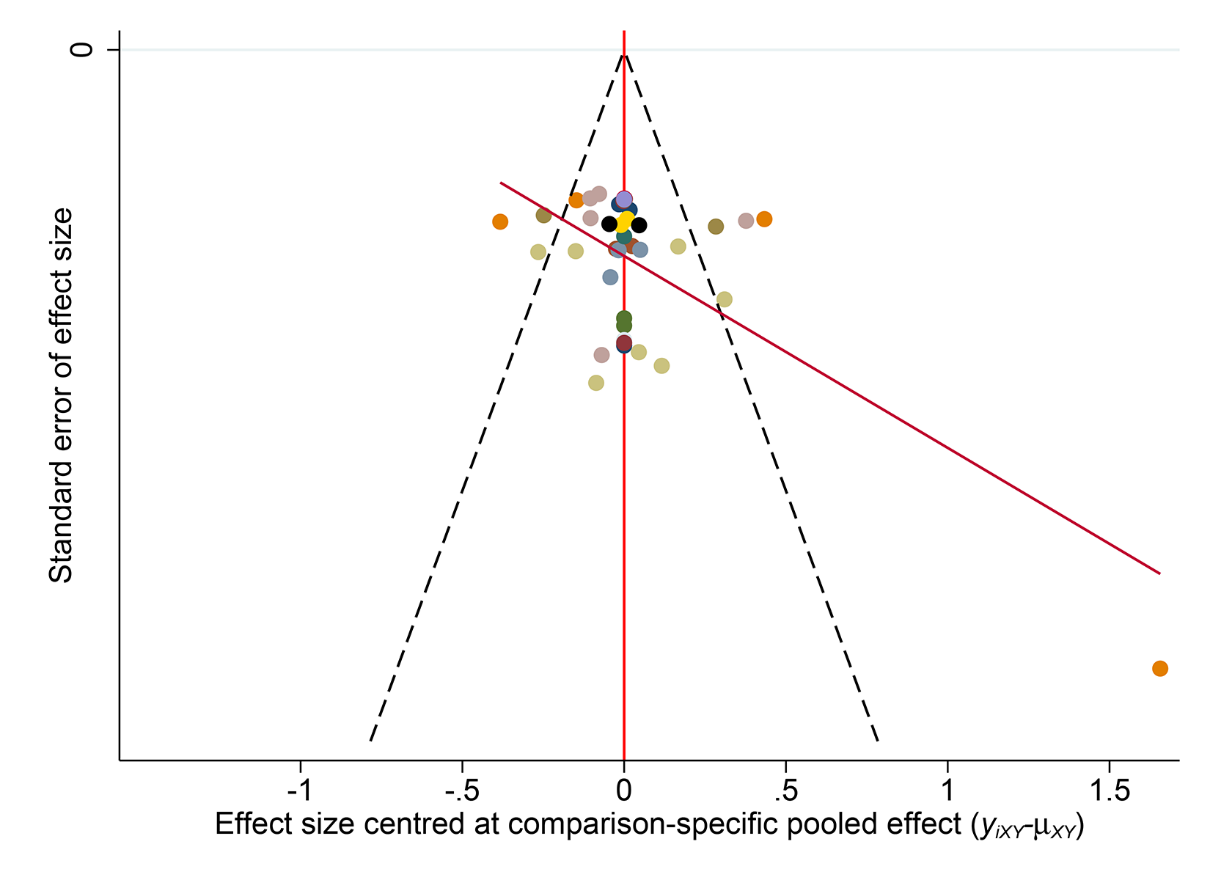


Comparison-adjusted funnel plot for efficacy on cognition (ADAS-cog)


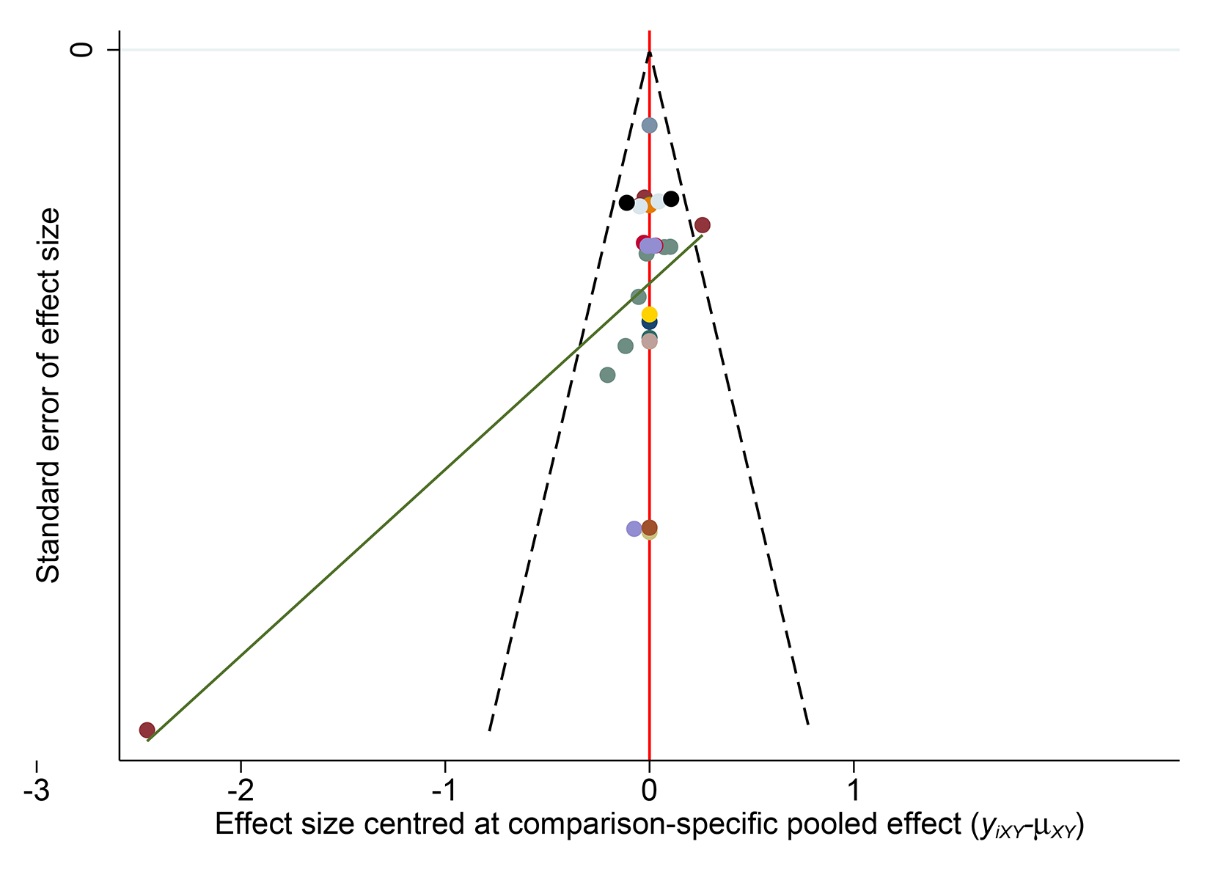


Comparison-adjusted funnel plot for efficacy on cognition (MMSE)


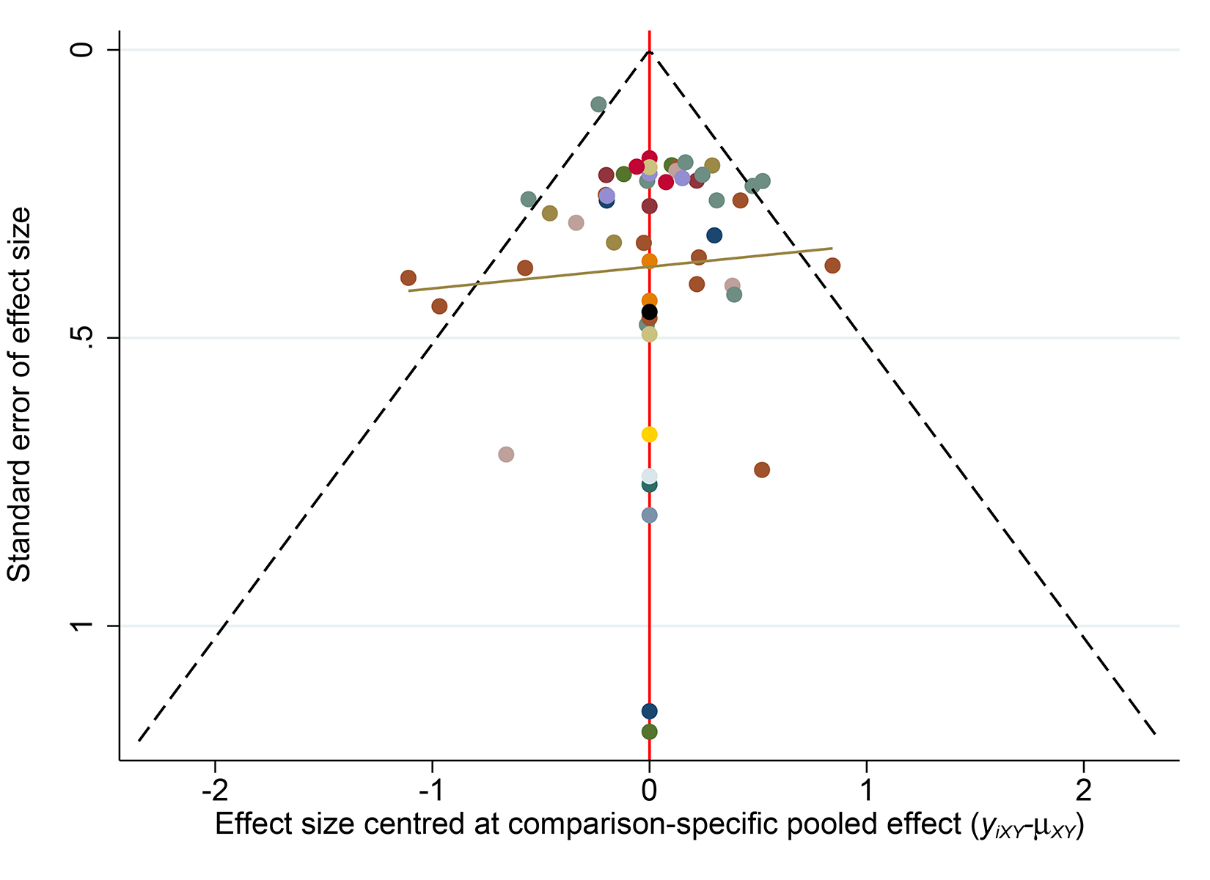


Comparison-adjusted funnel plot for acceptability


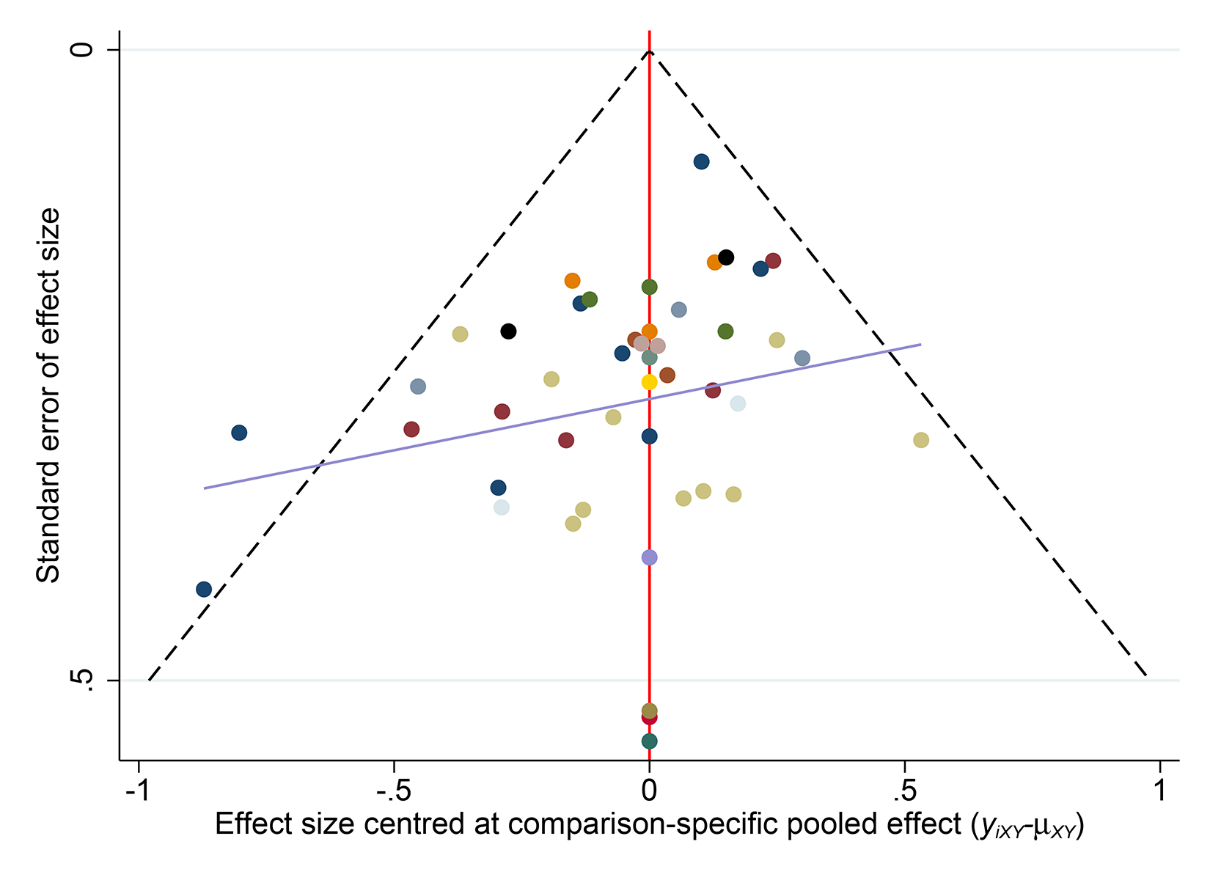


Comparison-adjusted funnel plot for safety


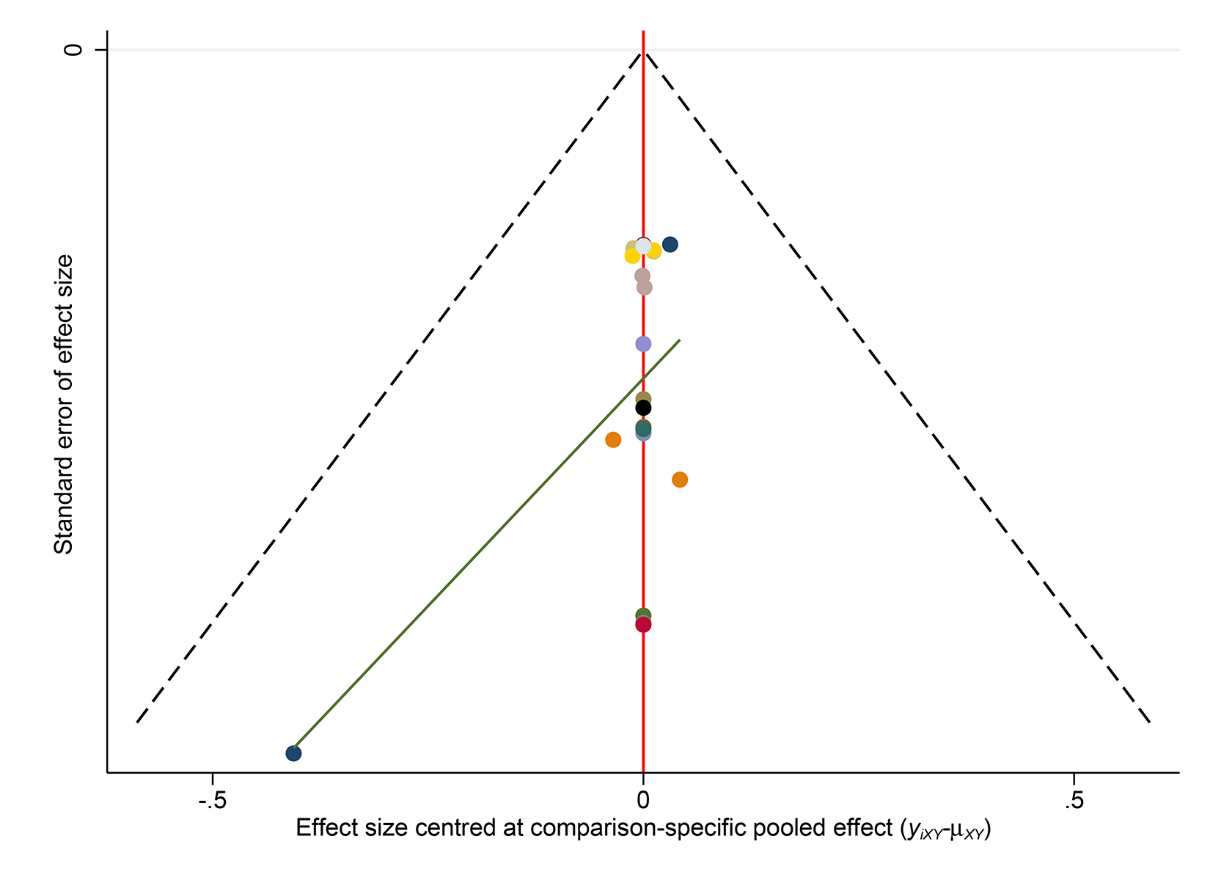


Comparison-adjusted funnel plot for efficacy on clinical global impression (CIBIC+)


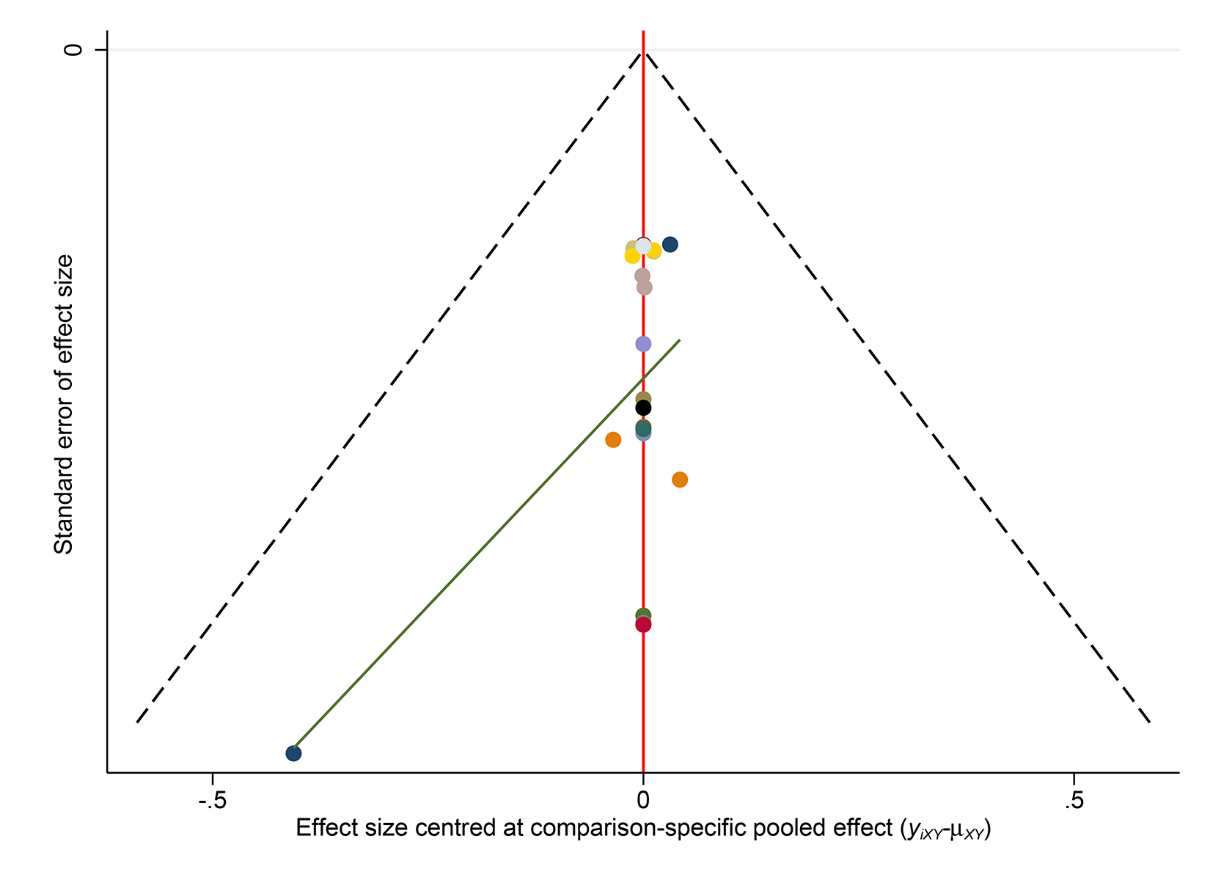


Comparison-adjusted funnel plot for efficacy on function (ADCS-ADL)


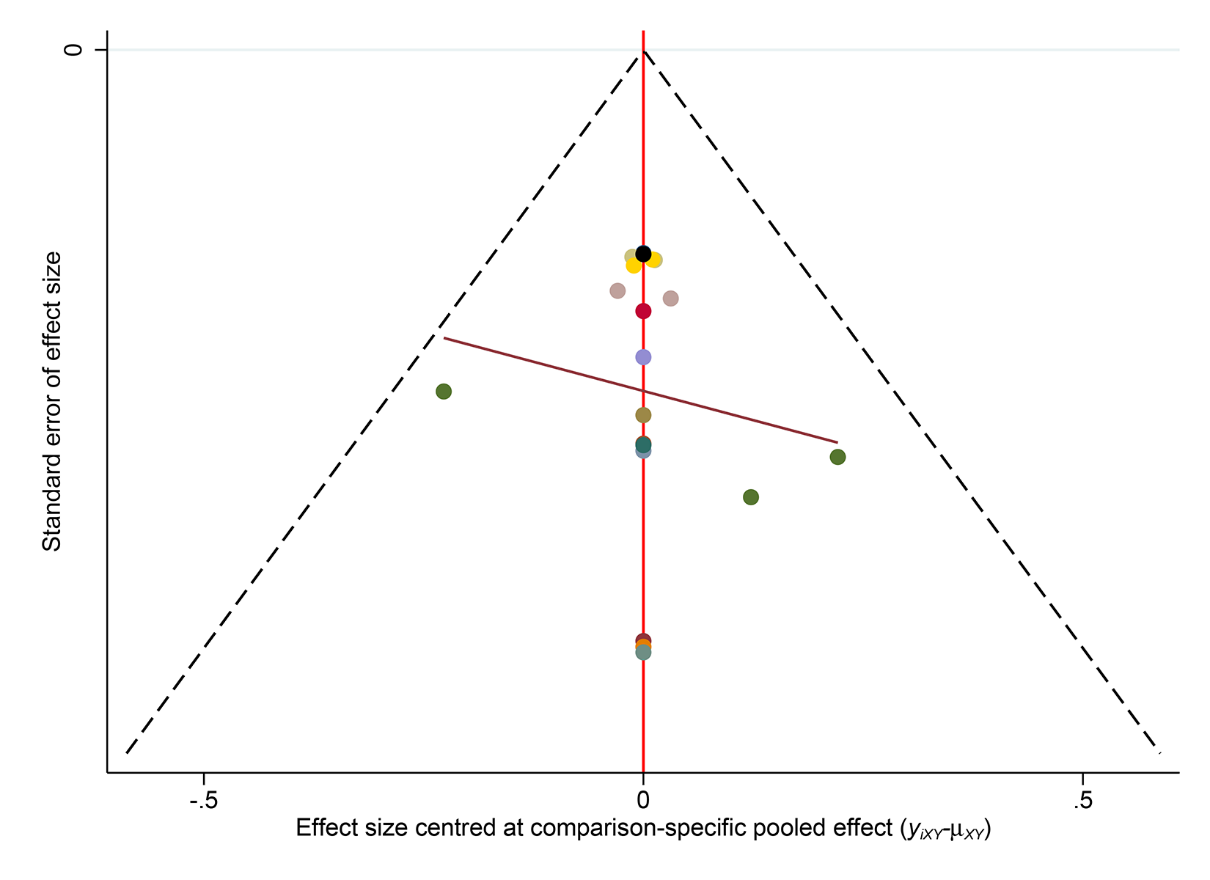


Comparison-adjusted funnel plot for efficacy on behavior (NPI)
